# Cheminformatics approach to exploring and modeling trait-associated metabolite profiles

**DOI:** 10.1186/s13321-019-0366-3

**Published:** 2019-06-24

**Authors:** Jeremy R. Ash, Melaine A. Kuenemann, Daniel Rotroff, Alison Motsinger-Reif, Denis Fourches

**Affiliations:** 1 Department of Chemistry, North Carolina State University, Raleigh, NC USA; 2 Department of Statistics, North Carolina State University, Raleigh, NC USA; 3 Bioinformatics Research Center, North Carolina State University, Raleigh, NC USA

**Keywords:** Metabolomics, Data mining, Cheminformatics, Molecular fragmentation, Statistics, Visualization, Chemical structure

## Abstract

Developing predictive and transparent approaches to the analysis of metabolite profiles across patient cohorts is of critical importance for understanding the events that trigger or modulate traits of interest (e.g., disease progression, drug metabolism, chemical risk assessment). However, metabolites’ chemical structures are still rarely used in the statistical modeling workflows that establish these trait-metabolite relationships. Herein, we present a novel cheminformatics-based approach capable of identifying predictive, interpretable, and reproducible trait-metabolite relationships. As a *proof*-*of*-*concept*, we utilize a previously published case study consisting of metabolite profiles from non-small-cell lung cancer (NSCLC) adenocarcinoma patients and healthy controls. By characterizing each structurally annotated metabolite using both computed molecular descriptors and patient metabolite concentration profiles, we show that these complementary features enhance the identification and understanding of key metabolites associated with cancer. Ultimately, we built multi-metabolite classification models for assessing patients’ cancer status using specific groups of metabolites identified based on high structural similarity through chemical clustering. We subsequently performed a metabolic pathway enrichment analysis to identify potential mechanistic relationships between metabolites and NSCLC adenocarcinoma. This cheminformatics-inspired approach relies on the metabolites’ structural features and chemical properties to provide critical information about metabolite-trait associations. This method could ultimately facilitate biological understanding and advance research based on metabolomics data, especially with respect to the identification of novel biomarkers.

**Electronic supplementary material:**

The online version of this article (10.1186/s13321-019-0366-3) contains supplementary material, which is available to authorized users.

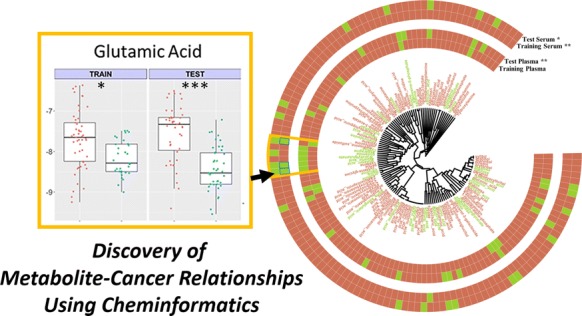

## Introduction

The metabolome is an individual’s phenotype at the molecular level [1–4]. Profiling metabolites (i.e., small molecule with molecular weight < 1500 Da) present in a given sample (e.g., serum, plasma, urine) enables in-depth investigations into various biochemical perturbations with internal (e.g., disease, drug metabolites, microbiome) and external (e.g., exposome, drugs) origins. A major advantage of metabolomic profiling over other *omics* methodologies is its high sensitivity to modulations of biological pathways that play a mechanistic role in these biochemical events.

The potential for certain metabolites to be discovered as disease biomarkers has resulted in a rapidly expanding body of metabolomics studies. For instance, metabolomics has been used to search for biomarkers for colon cancer [5, 6], multiple sclerosis [7], and Alzheimer’s disease [8–10]. Drug discovery efforts routinely use metabolomics to study the efficacy, toxicity, and pharmacokinetic/pharmacodynamic properties of drug candidates and their metabolites [11]. Furthermore, the field of pharmacometabolomics has emerged as a useful field to investigate the role of metabolites in drug response [12–14]. Metabolomics is utilized by medicinal chemists to investigate the in vivo mechanism of action of lead compounds and to more efficiently screen chemicals for their ability to cause adverse side effects.

While chemical structure is at the centerpiece of the metabolites’ structure elucidation stage in any metabolomics study [15–17], it is very often underutilized in the downstream trait association analysis. As underscored by recent papers [4, 18], the ability to conduct *in*-*depth* analysis of metabolomics datasets could be significantly improved through careful consideration of metabolites’ chemical structure. This is exactly what cheminformatics approaches have been developed for: to rapidly, quantitatively, and systematically characterize the structural features of chemicals via the standardized calculation of molecular descriptors [19]. Therefore, one can envision further representation of metabolites by computing quantitative molecular descriptors to characterize their chemical structures. In that regard, a recent analysis [20] comparing the similarity of drugs’ chemical structures with endogenous human metabolites found that 90% of marketed drugs have a *medium*-*to*-*high* similarity (Tanimoto > 0.5) to their most structurally similar human metabolite. Also recently, new algorithms have been developed to efficiently search metabolic networks using chemical fingerprints, demonstrating that metabolites in shared metabolic pathways have similar chemical structure [21]. The MetamapR network visualization tool [22, 23] has demonstrated that grouping metabolites by chemical classes can be used to generate hypotheses regarding the cellular processes related to an observed phenotype. The same research group has recently deployed ChemRICH [24], a tool for grouping metabolites by chemical similarity instead of biological annotation for enrichment analysis. However, to our knowledge, these methods have not been incorporated yet into a predictive modeling workflow. Overall, the chemical structures of metabolites are information-rich but have not yet been the centerpiece for a method to analyze and reliably model metabolomics datasets to establish more interpretable trait-metabolite relationships.

While there are numerous ways of determining enzymatic relationships between metabolites (e.g., by pathway or reaction pair databases [25–29]), these approaches are considerably limited by the lack of annotation of metabolic pathways, particularly for understudied organisms [22]. Detecting modules within metabolite profile correlation networks may capture some biochemical relationships between metabolites; however, this is complicated by the fact that neighbors in metabolic pathways do not always have high correlation [30] and confounding variations can be caused by other factors such as the transcriptional regulation of enzymes [31].

Multi-metabolite models (i.e., models that take as input multiple metabolite concentrations and predict a trait of interest) can improve upon the prediction performance of single metabolite models. However, single metabolite models are still mostly used in biomarker discovery, because multi-metabolite models often suffer in interpretability [32, 33]. In biochemical reactions, enzymes catalyze the conversion between chemically similar compounds, so binning metabolites according to their structural similarity for multi-metabolite models is likely to group metabolites that are biochemically linked, and that share the same trait-metabolite relationships [22, 23, 34]. This is intuitively appealing, since the biological effect of interest often operates at the level of biochemical pathways [35]. This approach may improve upon the predictivity of single metabolite models, while maintaining their desired interpretability, as the resulting models can still suggest pathways mechanistically linked to the trait of interest. The metabolites discovered by this approach and their associated pathways could then be investigated with complementary methods (e.g., targeted metabolomics, isotope labeling) [35]. The biochemical relatedness of the metabolites within these models could provide new interpretations of metabolomics data and potentially lead to trait-metabolite associations that would have otherwise been missed using alternative approaches.

Herein, we present a cheminformatics method [36] that leverages multi-metabolite modeling approach in conjunction with a chemical-informed clustering. We applied this approach to an adenocarcinoma lung cancer case study. Our main goal was to identify groups of structurally related metabolites linked to pathways with mechanistic and/or influential roles in lung cancer. We hypothesized that structure-based clustering of metabolites could help establishing more predictive, interpretable, and reproducible multi-metabolite classifiers for patient cancer status compared to alternative approaches.

## Results and discussion

### Subject sample collection and metabolomics profiling

As a *proof*-*of*-*concept* for our method, we considered a data set that was originally collected and analyzed by Fahrmann et al. [37]. This study identified blood biomarkers for adenocarcinoma lung cancer. Multi-metabolite models were shown to be highly predictive of cancer status in an independently conducted case study. Therefore, we wanted to determine if using metabolite chemical structure to form new multi-metabolite models could further improve the prediction performances, while enhancing the model’s interpretability.

The data set was accessed from the Metabolomics Workbench public repository (www.metabolomicsworkbench.org [38]) under study numbers ST000385 and ST000386. Subjects diagnosed with NSCLC stage I-IV adenocarcinoma were recruited by the UC Davis Medical Center and Cancer Center Clinics. Additional file 1: Table S1 shows the distribution of the patients’ characteristic variables (which were frequency matched by design). The patient characteristic distributions matched the data reported in Fahrmann et al. [37] except that one plasma cancer case was missing in the training set provided by the Metabolomics Workbench. The ADC1 (*training*) set contained 51 plasma and 49 serum samples collected from NSCLC stage I–IV adenocarcinoma patients. Plasma and serum samples were also collected from 31 healthy controls. The ADC2 (*test*) set consisted of 43 NSCLC stage I-IV adenocarcinoma patients and 43 healthy control plasma and serum samples. ADC2 was an *independently conducted* case study, meaning that the data was collected on different patients, and it was collected and analyzed at different times. This presented us with the opportunity to assess how well our predictive models would generalize to this independently collected, external test set. Importantly, the untargeted metabolomics analysis was performed by the same laboratory (WCMC Metabolomics Core at the University of California, Davis). Gas chromatography time-of-flight (GCTOF) mass spectrometry untargeted metabolomics analysis was performed using plasma and serum samples collected from each patient. Metabolites were structurally annotated by matching their mass spectra to the Fiehn library of 1200 authentic metabolite mass spectra [39]. Further details on the subject cohort, sample collection, metabolomics profiling can be found in Fahrmann et al. [37].

### Differences between training and test sets

**Fig. 1:**
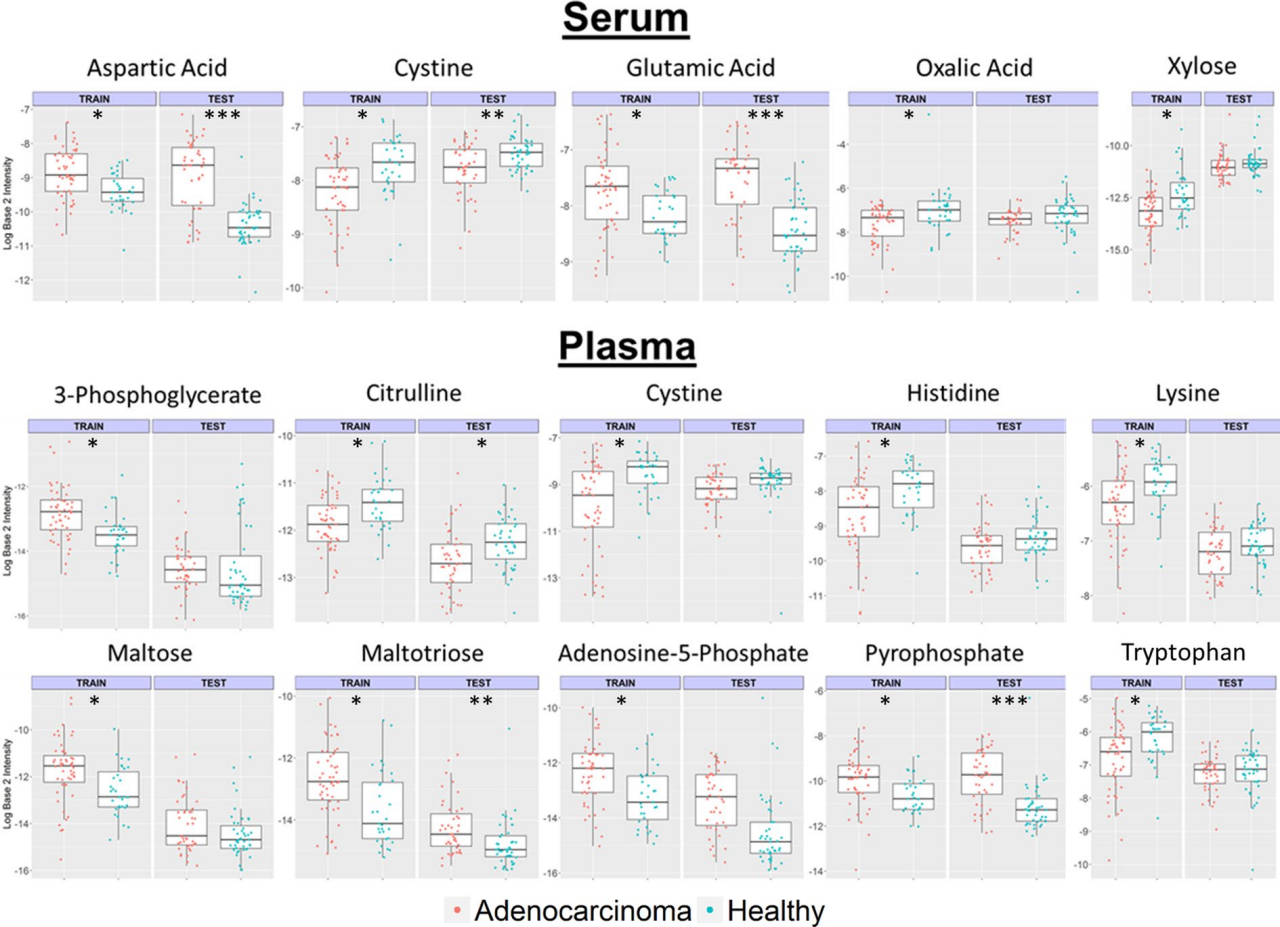
Distribution of intensities for metabolites significantly associated with cancer status in the training set. ADC1 (training) and ADC2 (test) set boxplots shown for healthy (blue) and adenocarcinoma (red) patients. Significant plasma and serum metabolites in ADC1 were determined by a paired *t* test. *(FDR < .075), ** (FDR < .01), *** (FDR < .001). Many of the metabolites that are significant in ADC1 are also significant in ADC2. Some show more significant differences in the ADC2

**Fig. 2:**
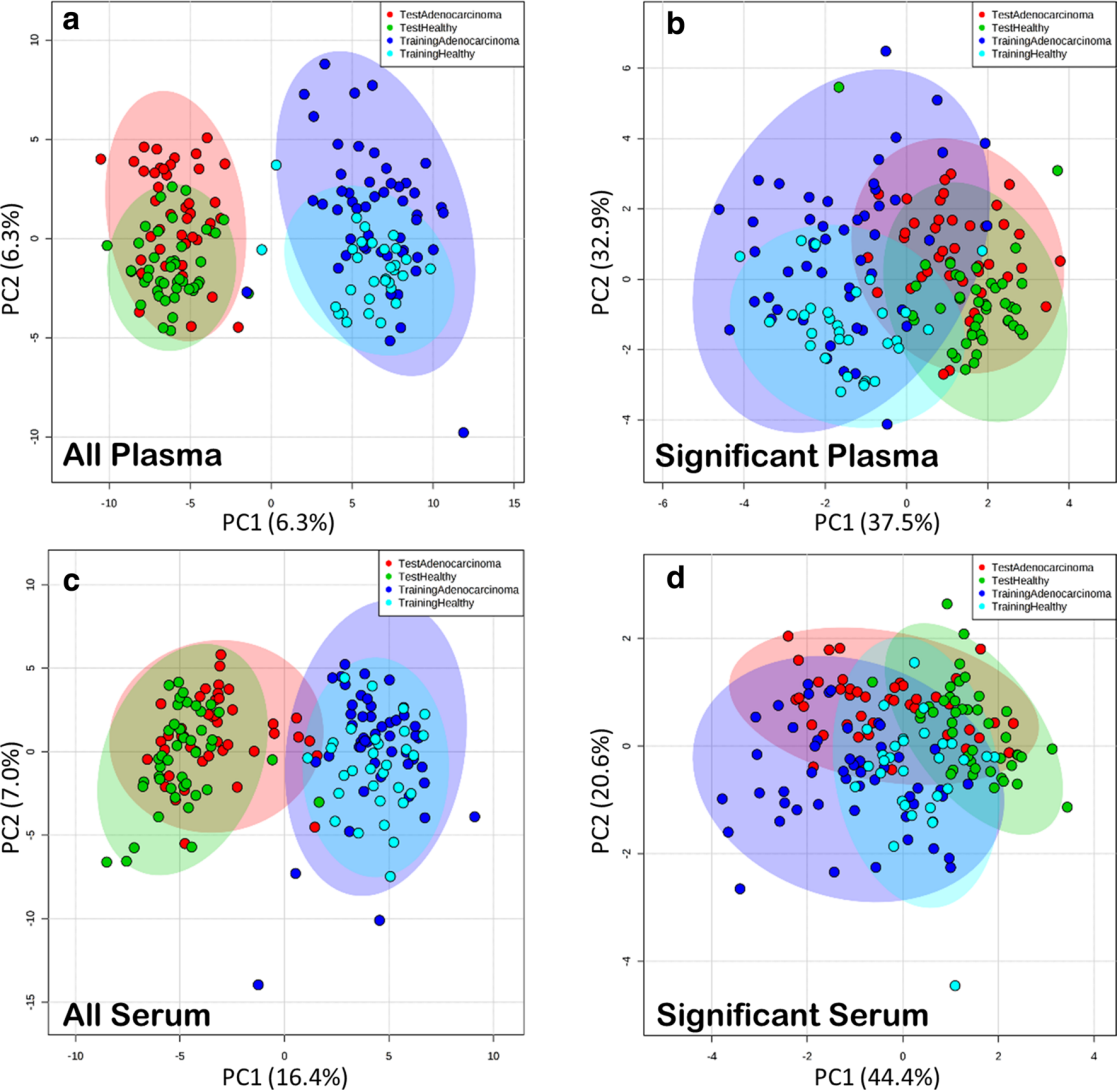
PCA of all metabolite and significantly different metabolite profiles. **a** All plasma, **b** significant plasma, **c** all serum, and **d** significant serum metabolite profiles

In total, 130 metabolites were retained based on the actual detection and availability of structural annotations in plasma and serum samples from both ADC1 and ADC2 sets (see Methods). Volcano plots identified metabolites with large fold changes in terms of mean relative abundance between cancer and control patients that were statistically significant (FDR < 0.075, Additional file 1: Figure S1). Surprisingly, metabolites with large shifts in distribution were not consistent across data sets (ADC1/ADC2) or tissues (plasma/serum). The disagreement between ADC1 and ADC2 suggested some level of heterogeneity between the ADC1 and ADC2 samples. Figure 1 illustrates how some metabolites (e.g., xylose, maltose, maltotriose) in ADC2 showed large shifts in intensities relative to ADC1 for both cancer and control patients. To further explore these differences, we conducted a PCA of the entire metabolite profiles of both ADC1 and ADC2 patients (Fig. 2a, b). We found large differences between the ADC1 and ADC2 patients in the same cancer status group, with a separation much larger than that between cancer and control patients. Both plasma and serum had much more variation within health state group (plasma BSS/TSS: .025, serum BSS/TSS: .023) than between health state. When metabolite profiles were filtered so that only the significant metabolites (i.e. those found with significant difference in mean intensities between cancer and control) for plasma or serum were utilized (Fig. 2c, d), variance explained by the health state group was higher, for serum in particular (plasma BSS/TSS: .082, serum BSS/TSS: .16), though the percentage of total variation explained by cancer status was still low. This indicated that models constructed using the entire set of ADC1 metabolites would be vulnerable to overfitting and likely demonstrate poor predictivity on the external ADC2 set.

Heatmaps for the 25 metabolites with the most significantly different mean log intensities between ADC1 and ADC2 prior to total quantity normalization further illustrate the differences between these data sets (Additional file 1: Figures S2 and S3). A few of these metabolites were significantly associated with cancer status and selected for our classification models (e.g., xylose and cystine in serum, maltose in plasma). However, later we will show that classifiers trained on these ADC1 metabolite profiles resulted in poor prediction performance for the ADC2 test set. As illustrated in Fig. 1, most of the metabolites with these large shifts in mean do not have the statistical ADC1-determined significance reproduced in ADC2. Many metabolites shown in Additional file 1: Figures S2 and S3 were not chosen for the classification models, but still had dramatic shifts in mean. These metabolites create substantial differences in bias for the ADC1 and ADC2 metabolites once total quantity normalization is performed. This could partially explain the lack of reproducibility of statistical significance of metabolites as shown in Fig. 1.

For an untargeted metabolomics analysis, total quantity normalization has been shown to be an effective strategy for controlling for many sources of analytical variation (e.g., extraction or derivatization process) in the absence of an accepted reference metabolite [40]. However, our results confirmed that this normalization strategy does not control for all variation between studies (which may be attributed to a number of causes, such as sample heterogeneity or methodological variation) [41]. Also, when there are large changes in metabolite intensities associated with a phenotype of interest, the normalization procedure can introduce bias that might lead to the detection of spurious associations in other metabolites [42]. To ensure the results are useful in clinical practice, researchers often conform to the clinical norm of total quantity normalization. Later, our classifier prediction performance results will suggest that the considerable noise that remains after total quantity normalization could be better controlled for by the careful construction of multi-metabolite classifiers.

### High structural similarity for metabolites significantly associated with cancer

The cheminformatics approach described in this study is based on characterizing metabolites’ structural properties by computing molecular fingerprints and then clustering those metabolites based on chemical similarity. We used the MACCS 166 bit fingerprint [43], a standard class of structural keys used in the cheminformatics community, to represent and characterize metabolites’ chemical structures instead of the 881 bit PubChem fingerprints used by MetaMapR and ChemRICH [22–24]. Both types of fingerprints are interpretable, with bits simply indicating presence/absence of chemical substructures. The MACCS fingerprint was selected because in a number of studies on the use of molecular fingerprints for de novo reconstruction of metabolic networks [44, 45], more complex fingerprints (e.g., PubChem and the extended CDK fingerprint [46]) were shown to only have marginally improved prediction performances. Practically, we noticed little change in the clustering results when the ECFP6 [47] fingerprints were used to encode metabolite structures (results not shown). MACCS keys were thus selected due to their simplicity and interpretability.

**Fig. 3:**
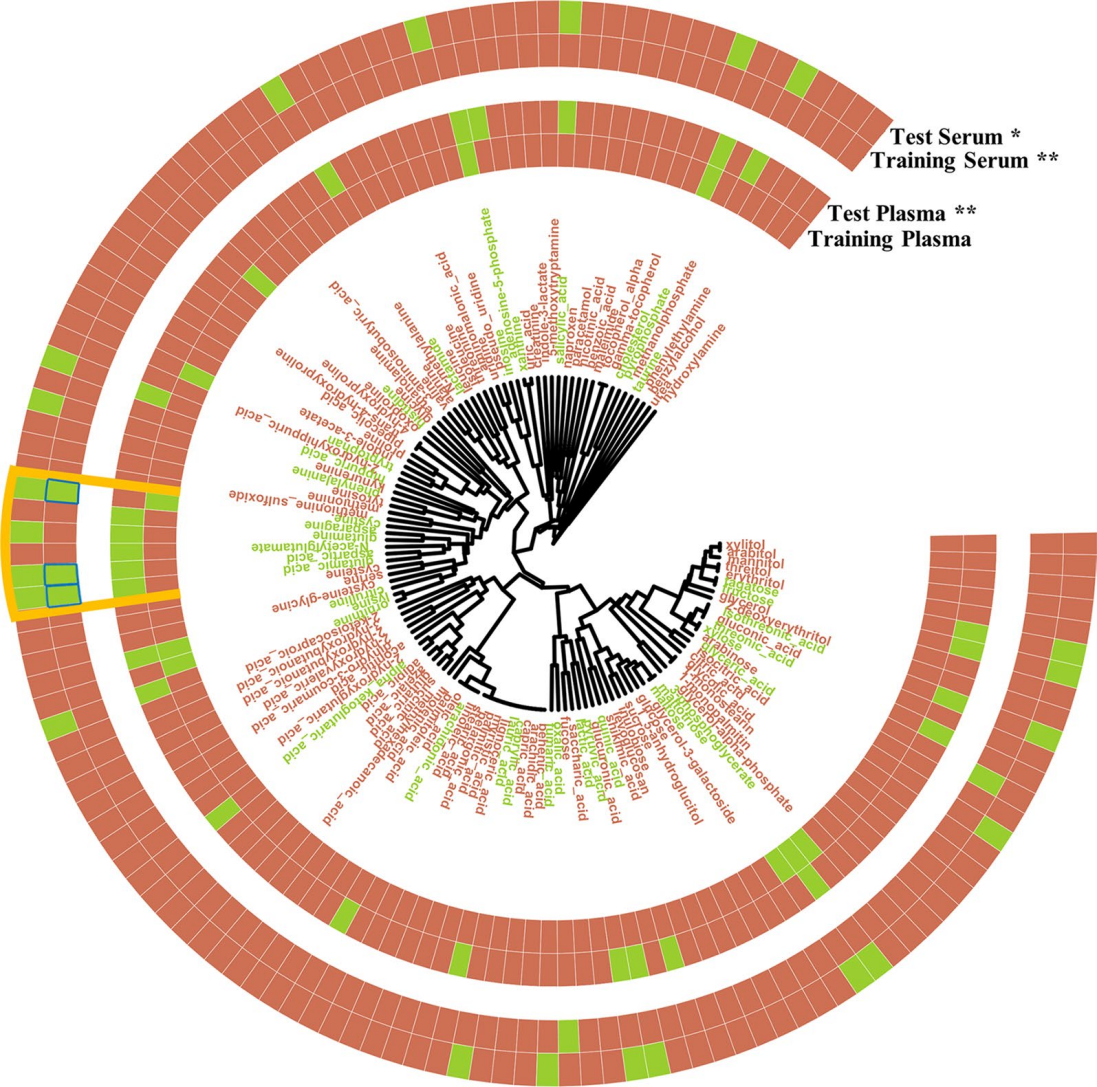
Integrated circular dendrogram generated using MACCS fingerprint with average linkage and Soergel distance. A cell next to a metabolite name is colored green if the metabolite has a significant difference in mean relative abundance between for cancer versus control patients in one of the data sets (ADC1/ADC2, serum/plasma) after correction for multiple testing. Metabolites names are colored green if they were significant in at least one data set. Fisher exact test for greater probability of significance for metabolites within the highlighted cluster (orange) than those without *(FDR < .05), ** (FDR < .01), *** (FDR < .001). The metabolites highlighted in blue were selected by our multi-metabolite procedure to form the best classifier *without* using information about the test set

To examine whether the metabolites significantly associated with health status also had similar chemical structures, we conducted a hierarchical clustering of all the metabolites based on this fingerprint. Interestingly, Fig. 3 highlights a cluster containing a high proportion of significant metabolites across each data set. In other words, we identified a set of structurally similar metabolites whose significant differences are reproducible across samples (plasma and serum) and data sets (ADC1 and ADC2). This provides multiple lines of evidence that these associations are biologically meaningful and not an artifact of the sample preparation or methodology.

**Fig. 4:**
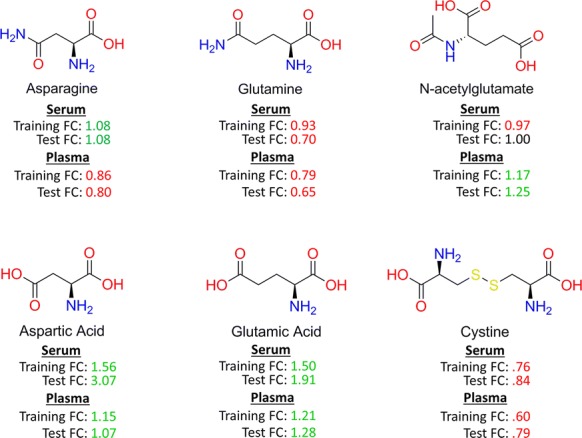
Metabolite structures from the cluster containing a large proportion of significant metabolites. Mean metabolite abundance fold change for cancer versus healthy patients in Serum and Plasma ADC1 and ADC2 data sets

**Fig. 5:**
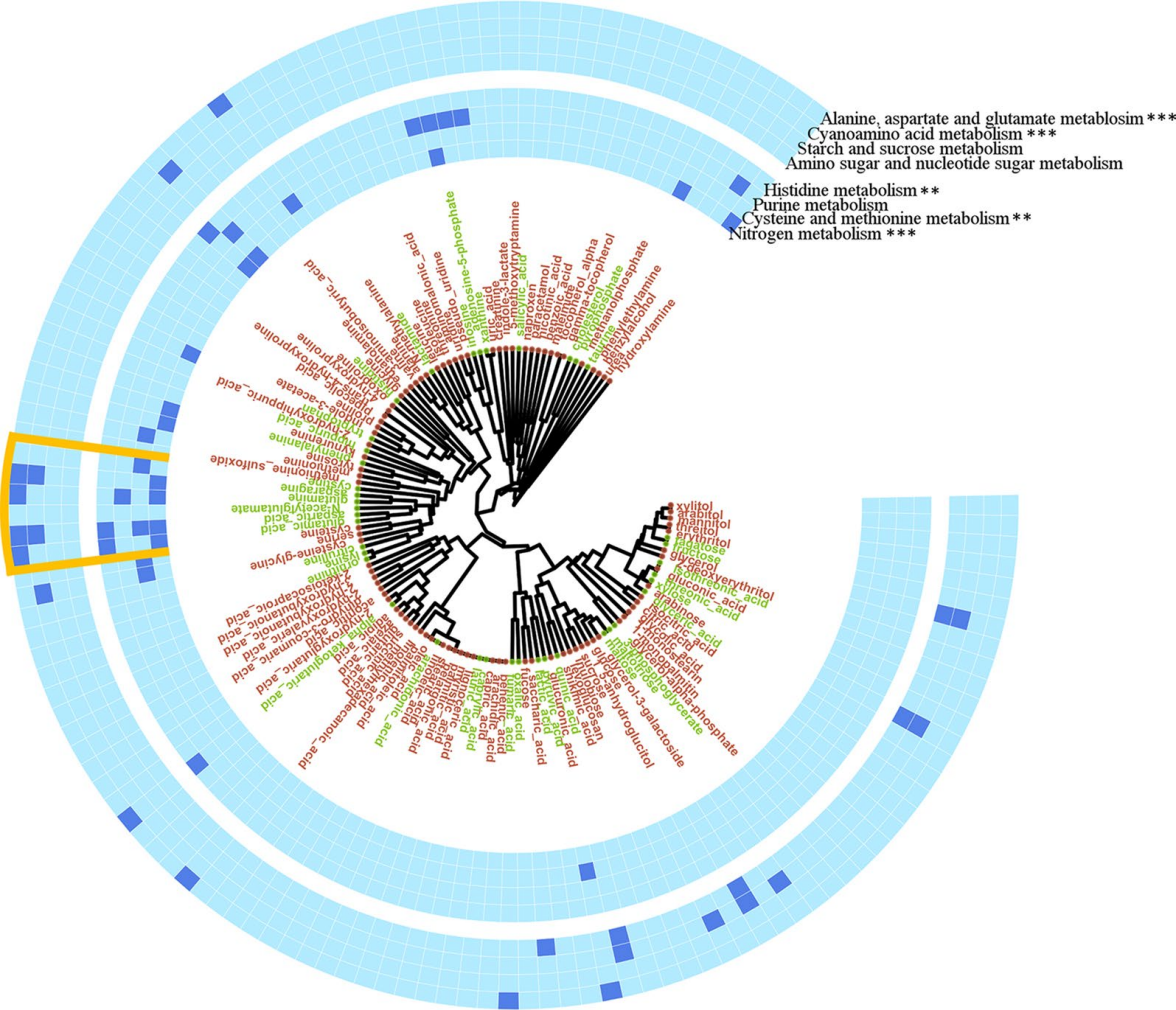
Integrated circular dendrogram generated using MACCS fingerprint with average linkage and Soergel distance. Cells next to metabolites names are colored dark blue if they belong to a pathway significantly enriched (hypergeometric test; FDR < .05) for metabolites found to be significant in the differential analysis for ADC1 serum (top band) or ADC1 plasma (bottom band). Metabolites names are colored green if they were significant in at least one data set. Fisher exact test for greater probability of pathway membership for metabolites within the highlighted cluster (orange) than those without *(FDR < .05), ** (FDR < .01), *** (FDR < .001)

For all data sets except ADC1 plasma, there were more significant metabolites in the chemically similar cluster than would be expected by chance (FDR < .05). This cluster contained glutamic acid, aspartic acid, glutamine, asparagine, N-acetylglutamate, and cystine (Fig. 4). All of these metabolite profiles except cystine were found significant in the plasma ADC2 set (Fig. 1). Importantly, aspartic acid, glutamic acid, and cystine were significant in both ADC1 and ADC2 serum sets. These metabolites were clustered together because of their high structural similarity: as amino acids or close derivatives, they share several key functional groups (e.g., carboxylic acid, amine, amide) and every metabolite except cystine has a primary, aliphatic carbon chain including 4 or 5 carbons. Figure 5 shows the same metabolite dendrogram as Fig. 3, except that metabolites were labeled based on their associated membership in the actual biological pathways found to have significant differences between cancer and control patients according to our pathway enrichment analysis (FDR < .05). Again, we analyzed the same cluster containing a high proportion of significant metabolites across samples and data sets. For each pathway, we performed an enrichment analysis as was done for Fig. 3.

In serum and plasma, several significantly enriched metabolic pathways were found to have more metabolites in the cluster of interest than would be expected by chance. Two pathways in Serum (alanine, aspartate, and glutamate metabolism; cyanoamino acid metabolism) and three pathways in Plasma (histidine metabolism, purine metabolism, and nitrogen metabolism) showed enrichment for significant metabolites within that particular cluster of interest (FDR < .05). Interestingly, N-acetylglutamate was not a member of any significantly enriched pathway. In plasma, four metabolites (glutamic acid, aspartic acid, glutamine, and asparagine) belong to the significantly enriched nitrogen metabolism pathway (FDR = 0.0097). In this particular pathway, both asparagine and glutamine are metabolized to aspartic and glutamic acid, respectively, which is reflected in the concentration of these metabolites for cancer and healthy patients (Fig. 4). Glutamine and asparagine showed a large decrease in mean concentration for patients with cancer, while aspartic acid and glutamic acid showed a large increase. In serum, the alanine, aspartate and glutamate metabolism pathway also contained these four metabolites (FDR = 0.022).

Our “cluster enrichment analysis” found that several significantly affected pathways contained more metabolites in the chemically similar cluster than would be expected by chance. This suggested that compounds in pathways significantly affected by adenocarcinoma can be clustered by chemical similarity. In fact, multiple pairs of metabolites in our cluster of interest are interconverted by one chemical reaction (e.g., asparagine to aspartic acid).

The adenocarcinoma-associated decrease in glutamine and increase in glutamic acid is particularly interesting, as altered glutamine metabolism has been shown to play a critical role in the malignancy of lung cancer cells [37, 48]. This significant increase in glutamate levels has been observed in recent studies [49, 50], in which an increased reliance on glutamine instead of glucose for oxidative phosphorylation and NADPH generation has been suggested. The adenocarcinoma associated reduction in cystine has also been reproduced [49]. The SLC7A11 amino acid antiporter, which may also be up-regulated in adenocarcinoma [49], imports cystine and exports glutamate. A reduction of cystine could indicate an increase in its intracellular concentration as well as its reduction product, cysteine, which could regulate glutathione biosynthesis [49, 51].

It was particularly interesting that 4 out of 6 metabolites (glutamic acid, aspartic acid, cystine and glutamine) in the cluster of interest were found to be significant in both blood matrices. It has been previously noted that, although some metabolite concentrations may be higher on average in serum than plasma (resulting in higher sensitivity to detect significant low abundance metabolites), many of the metabolites with significant differences in both matrices were highly correlated [52, 53]. However, differences in the blood matrix and sample preparation are likely to result in some discrepancies between metabolite profiles [52, 53]. Fahrmann et al. [37] recommended using serum blood matrix for diagnosis of adenocarcinoma due to its higher sensitivity. Therefore, when we constructed the metabolite classifiers, we focused on optimizing the prediction performance of serum classifiers.

### Additional cheminformatics clustering of significant plasma and serum metabolites according to their chemical structure

Our analysis in the previous section suggested that, when there is a cluster of chemically similar metabolites significantly associated with adenocarcinoma, the relationship is potentially highly reproducible, and a mechanistic interpretation can be assigned to that cluster. Below, we tested whether this principle could be incorporated into a predictive modeling workflow.

In order to bin metabolites for the construction of multi-metabolite classifiers, the set of metabolites that were significantly associated with cancer status in either serum or plasma ADC1 were further clustered according to chemical structure similarity. The method for deciding the optimal number of clusters when using hierarchical clustering is illustrated in the Additional file 1: Figure S4, along with the heat maps for the corresponding distance matrix and cluster assignments.

For serum samples, only one cluster contained multiple significant metabolites (aspartic acid, glutamic acid and cystine) (Additional file 1: Figure S4). Clustering of plasma metabolites resulted in several clusters with multiple metabolites (Cluster 1: 3-phosphoglycerate, pyrophosphate; Cluster 2: citrulline, cystine, histidine, lysine; Cluster 3: maltose, maltotriose).

We also tested whether the same clusters of metabolites could be detected by the Spearman or partial correlations of patient metabolic profiles (which means absolutely no information about chemical structures). Hierarchical clustering was performed using either Spearman or partial correlation distances (1—absolute correlation) and the average linkage between clusters (Additional file 1: Figure S5). The cluster assignments differed from those obtained by chemical structure in serum—only glutamic acid and aspartic acid were clustered together and cystine was in a separate cluster. The cluster assignments also significantly differed for plasma, where only two clusters were identified in both the spearman and partial correlation networks (Cluster 1: histidine, cystine, tryptophan, lysine, citrulline; Cluster 2: maltotriose, maltose, adenosine-5-phosphate, pyrophosphate, 3-phosphoglycerate).

We also tested whether the similar clusters of metabolites could be detected by MetaMapp or ChemRICH approaches (see Method section). Additional file 1: Figure S6 shows the MetaMapp networks inferred for metabolites significant in serum and plasma. ChemRICH did not find any significantly enriched clusters when adjusted p values were provided for serum or plasma metabolites. Instead, we provided unadjusted p values. Additional file 1: Figure S7 shows the significantly enriched clusters in serum and plasma. The cluster assignments found by both methods differed from our chemical structure clustering approach. See the Additional file 1: Results and Discussion for further discussion.

### Serum classifiers trained on ADC1 and validated with ADC2

Serum logistic regression classification models were constructed utilizing only metabolites that were found to be significant in the serum ADC1 training set. Table 1 shows the LOOCV prediction performances afforded by the single- and multi-metabolite classifiers. Cystine and Oxalic Acid were the best performing single-metabolite models with both models affording 70% prediction accuracy to separate cancer patients versus healthy controls. When significant serum metabolites were clustered to form multi-metabolite models, only one cluster contained multiple metabolites (aspartic acid, glutamic acid and cystine) (Additional file 1: Figure S4). Importantly, the multi-metabolite approach based on these three selected metabolites performed considerably better than any single metabolite approach with an overall prediction accuracy of 76.3%. This model afforded a good sensitivity (77.6%), while maintaining high specificity (74.2%).

**Table 1:** Performance measures for selected serum models predicting cancer status

	ADC1 (training) LOOCV				ADC2 (test) external validation			
	Accuracy (%)	Sensitivity (%)	Specificity (%)	AUC	Accuracy (%)	Sensitivity (%)	Specificity (%)	AUC
Single metabolite classifiers								
Aspartic Acid	62.5	40.8	96.8	0.698	79.1	62.8	95.3	0.862
Cystine	70.0*	75.5	61.3	0.685	55.8*	76.7	34.9	0.677
Glutamic Acid	62.5	42.9	93.5	0.687	76.7	65.1	88.4	0.846
Oxalic Acid	70.0*	83.7	48.4	0.65	57.0*	88.4	25.6	0.649
Multi-metabolite classifiers—clustered metabolites								
Cluster 1^a^ SVM^b^	76.3*	77.6	74.2	0.751	84.9*	72.1	97.7	0.856

Asterisk represents best model accuracies according to LOOCV. Best model accuracies according to external validation accuracy are underlined

^a^Aspartic acid, cystine, glutamic acid

^b^Support vector machines

Validation with an external test set is essential to determining the efficacy of a predictive model [54]. In order to assess the utility of these classifiers for an independently conducted case study, the models were trained using all ADC1 serum samples and their external prediction performances were assessed on the ADC2 set. This enabled us to assess how well our models generalized to an independent study in the presence of considerable batch effects (Figs. 1, 2).

The optimal serum single metabolite models that would have been selected according to LOOCV accuracy on ADC1 were cystine and oxalic acid (70%). However, these single metabolite classifiers afforded very poor prediction accuracy on the ADC2 test set (55.8 and 57%, respectively). Importantly, if the best performing model was selected according to LOOCV accuracy on the ADC1 training set, the SVM multi-metabolite model using a cluster of metabolites (aspartic acid, glutamic acid and cystine) would have been selected (76.3%). This model consisted of all of the metabolites in the previously highlighted cluster of interest that were significant in serum ADC1. The classifier obtained better prediction accuracy on the ADC2 test set than any other serum classifier (84.9%). This model had very high specificity (97.7%), while maintaining high sensitivity (72.1%). This is desirable, as these biomarkers were intended to complement other diagnostic tools that have a high false positive rate, like low dose computed tomography [37]. This result is significant not only because this model demonstrated the best prediction performances for both ADC1 and ADC2, but also because the internal LOOCV procedure employed for the ADC1 set selected the classifier with the best prediction performance for ADC2, indicating that this multi-metabolite ensemble method has good prediction performance in independent case studies. The results obtained for all models (and not only the best performers) are given in Additional file 1: Tables S3 and S4.

The same procedures used to train the multi-metabolite classifiers on clustered metabolites was used to train multi-metabolite classifiers on all metabolite and significant metabolite profiles (Additional file 1: Table S3). On ADC1, the best performing approaches obtained higher LOOCV accuracies (81.3% and 83.8% respectively), but these did not generalize well to ADC2 (50.0% and 59.3%). This was likely due to the fact that these models included noise variables – metabolites like xylose that showed significant separation between cancer control in ADC1, but not ADC2.

The serum metabolites within our best multi-metabolite classifier were linked by multiple pathways (e.g., alanine, aspartate and glutamate metabolism, cysteine and methionine metabolism). The serum metabolites in our model provide several lines of evidence for sharing a common biological mechanism in NSCLC adenocarcinoma. Furthermore, these metabolites clustered together, while all others clustered into singletons, demonstrating that this method can group biologically meaningful metabolites, distinguishing them from the noise and leading to improvements in prediction performance. Taken together, these results support our conclusion that our cheminformatics-based approach to the construction of multi-metabolite classifiers provided us with a more predictive model than more traditional single metabolite approaches, while maintaining the interpretability of single metabolite models.

In their original study, Fahrmann et al. [37] built single metabolite classifiers on metabolites significant associated with adenocarcinoma and then built multi-metabolite classifiers by iteratively including them into ensembles. The single metabolite classifier with the highest accuracy was added first, then the classifier with the next highest accuracy was added, and so on. The multi-metabolite ensembles classified samples by majority vote, and the prediction performance was evaluated at each iteration. By performing chemical structure clustering on the significant metabolites, our multi-metabolite classifier approach was able to better separate signal variables from the noise. This resulted in improved prediction performance compared to multi-metabolite classifier that would have been selected based on LOOCV accuracy in the original study (ADC1 72.5%, ADC2 64.0%). Further advantages of our method are that the metabolites in our best multi-metabolites classifiers have known chemical structures and are biochemically linked because they are chemically similar.

### Plasma classifiers trained on ADC1 and validated with ADC2

Similarly, plasma classification models were constructed utilizing the plasma metabolites found significant in the ADC1 training set. Table 2 shows the LOOCV prediction performances obtained on the ADC1 set for single- and multi-metabolite classifiers. Maltose was the best performing single metabolite model (74.4% accuracy). However, this model demonstrated poor predictive performance on the ADC2 data set (57% accuracy). Chemical structure clustering of the plasma metabolites resulted in three clusters with more than one metabolite (Additional file 1: Figure S4). The optimal multi-metabolite classifier was the SVM model trained on the 3-phosphoglycerate and pyrophosphate cluster (80.5% LOOCV accuracy) and this model also led to relatively high prediction accuracy on the ADC2 external set (70.9% accuracy). The results obtained for all models (and not only the best performers) are given in Additional file 1: Tables S4 and S5.

**Table 2:** Performance measures for selected plasma models predicting cancer status

	ADC 1 (training) LOOCV				ADC2 (test) external validation			
	Accuracy (%)	Sensitivity (%)	Specificity (%)	AUC	Accuracy (%)	Sensitivity (%)	Specificity (%)	AUC
Single metabolite classifiers								
3-phosphoglycerate	70.7	60.8	87.1	0.734	51.2	34.9	67.4	0.578
Maltose	74.4*	82.4	61.3	0.701	57.0*	62.8	51.2	0.607
Pyrophosphate	69.5	66.7	74.2	0.703	76.7	67.4	86.0	0.811
Multi-metabolite classifiers—clustered metabolites								
Cluster 1^a^ SVM^b^	80.5*	86.3	71.0	0.713	70.9*	72.1	69.8	0.675

Asterisk represents best model accuracies according to LOOCV accuracy. Best model accuracies according to external validation accuracy are underlined

^a^3-Phosphoglycerate, pyrophosphate

^b^Support vector machines

This modeling method also improved on the multi-metabolite classifiers built on all metabolites (ADC1 79.3%, ADC2 69.8%), and significant metabolites (ADC1 78.0%, ADC2 69.8%) (Additional file 1: Table S5). Again, this supports our conclusion that our multi-metabolite approach results in reproducible and robust prediction performances. The prediction accuracies were comparable to that obtained by the five metabolite ensemble reported in the Fahrmann et al. [37] study (79.5% ADC1, 73.3% ADC2), but again the ensemble metabolites in our model are biochemically linked. Pyrophosphate and 3-phosphoglycerate were not grouped by any annotated pathway, but are biochemically linked. Phosphoglycerate kinase catalyzes the transfer of phosphate from 1,3-bisphosphoglycerate to ADP, forming 3-phosphoglycerate and ATP, which can then be hydrolyzed to ADP and pyrophosphate. Recent studies have reproduced the adenocarcinoma associated reduction of 3-phosphoglycerate [49, 50].

Altogether, the metabolite classifier results suggested that, when the difference between the cancer and control patient metabolite profiles was substantial enough, the signal in the data could be detected by single metabolite classifiers in both the ADC1 and ADC2 set. This is true despite the noise introduced by unwanted sources of variation observed in Figs. 1 and 2. However, building multi-metabolite classifiers based on clusters of metabolites with high similarity in chemical structure was determined to be an effective strategy for further removing noise in the data and improving prediction performances.

## Materials and methods

### Data preprocessing

Unless otherwise noted, all data analysis was performed in R (v3.3.2, [55]). To ensure that models were being built with the same metabolite profiles in ADC1/ADC2 and Plasma/Serum, only retained for the analysis were the 130 metabolites that (1) had fully determined chemical structures and (2) were detected in at least one sample in each data set. Missing metabolite intensities were imputed using half of the minimum intensity observed for that metabolite. Intensities were total quantity normalized and log base 2 transformed to correct for non-homogeneity of variance. Importantly, the curated datasets as well as all scripts used for this study are provided in the Additional file 2 to ensure the reproducibility of this study.

### Differences between training and test sets

The differences between the ADC1 and ADC2 samples were analyzed by means of principal components analysis (PCA) and metabolite profile heat maps using MetaboAnalyst [28]. Metabolite profiles were auto-scaled prior to these analyses. We quantified the metabolite profile variation between patients in the same health state group using *within* group sum of squares (WSS) and the metabolite profile variation between health state groups using *between* group sum of squares (BSS). The proportion of total metabolite variation explained by cancer state can be quantified using the between group sum of squares and total sum of squares ratio (BSS/(BSS + WSS) = BSS/TSS).

### Pathway analysis

Pathway overrepresentation analysis was conducted using MetaboAnalyst [28, 56] v3.0. The KEGG [57] Homo sapiens pathway library was used for the pathway enrichment analysis. A hypergeometric test (FDR < .05) was used to determine the pathways that contained more metabolites significantly associated with cancer status than would be expected by chance.

### Differential analysis

A differential analysis on the ADC1 training set determined the existence of significant differences between case and control mean metabolite intensities according to the procedure reported in Fahrmann et al. [37] Metabolite profiles were “covariate adjusted”, meaning they were regressed on the gender and smoking history of each subject and the residuals were used for differential analysis. Univariate Monte Carlo permutation t-tests [58] were performed for each metabolite separately (100,000 permutations) using the package *deducer* [59]. A Benjamini–Hochberg correction for multiple testing [60] was employed with FDR significance threshold of 0.075. This FDR threshold found significant all of the structurally annotated metabolites reported by Fahrmann et al. [37] in both serum and plasma. To determine the reproducibility of the metabolites found to be significantly associated with adenocarcinoma in ADC1, differential analysis was performed in the same way on the ADC2 test set. For the ADC1 training set, significance was used as the criterion for the selection of metabolites for cancer status classifiers. This criterion was utilized because we expected significant metabolites to provide good classification of cancer status that is reproducible in independent studies. Volcano plots demonstrating the association analysis results were created by Metabolomics Workbench [38].

### Cheminformatics clustering based on metabolites’ chemical structures

The names of structurally annotated metabolites were provided by the Metabolomics Workbench. We automatically retrieved the chemical structures for all 130 metabolites using the PubChem API [61]. Importantly, all structures were standardized according to our previously published chemical curation protocols [62–64]. Then, metabolite chemical structures were characterized using MACCS fingerprints [43] computed using the RDKit node [65] in Knime [66]. A Pearson correlation coefficient cutoff of 0.9 was used to filter out the highly correlated bits in the fingerprints. Hierarchical clustering of metabolites based on their chemical structure encoded as MACCS fingerprints was performed according to Soergel distances (also known as Tanimoto distances) and the average linkage. The *ggtree* package [67] was used to create circular dendrograms, and clustering of the significant metabolites for the construction of multi-metabolite models proceeded in the same way.

As part of the hierarchical clustering procedure, the number of clusters (*k*) was selected in order to achieve a reasonable partitioning of the metabolites. We selected the *k* value that resulted in the highest average silhouette width (ASW) [68] for cluster assignments. By maximizing the ASW, we aimed to find the most “natural” number of clusters in the data, in which cluster members are most similar to each other, and distant from members belonging to other clusters. More formally, let *a*(*i*) be the average distance between metabolite *i* and all other members of its cluster, and *b*(*i*) be the smallest average distance between metabolite *i* and the members of any other cluster. Then the silhouette for metabolite *i*, *s*(*i*), is:

$$s\left( i \right) = \frac{b\left( i \right) - a\left( i \right)}{{\hbox{max} \left\{ {a\left( i \right), b\left( i \right)} \right\}}}$$

Overall, the ASW is the average of the silhouette values for all *i* metabolites.

### Alternate metabolite clustering methods

Alternate methods of grouping metabolites for multi-metabolite classifiers were also considered. To determine if the same cluster assignments could be obtained by the correlation of the covariate adjusted metabolite profiles alone, hierarchical clustering was performed using a correlation based distance (1 – absolute correlation). Spearman correlation was calculated to avoid parametric assumption of Pearson correlations. Partial correlations were computed by the package *parcor* [69]. Since there were more metabolites than samples, we used partial least squares (PLS) regression to regularize the estimation of the regression coefficients before computing the partial correlations [69]. We used 10-fold cross validation to select the number of PLS components (with a maximum of 30).

We also considered two alternate methodologies that utilize chemical structure to cluster metabolites—MetaMapp [22] and ChemRICH [24]. MetaMapp was used to infer a network between plasma and serum metabolites. One set of edges were drawn between metabolites that were similar in chemical structure, based on a 0.7 threshold of Tanimoto similarity of their PubChem fingerprints. Another set of edges were drawn between metabolites that can be interconverted by a single reaction. Cytoscape’s “organic layout” was used to find a natural clustering in the network based on node degree and clustering coefficient.

ChemRICH groups metabolites together based on their chemical ontologies in the Medical Subject Headings (MeSH) database. If a metabolite is not annotated, it is labeled with the ontology of compounds with highly similar PubChem fingerprints. If metabolites can still not be annotated, ChemRICH has a mechanism for detecting novel groups of metabolites based on chemical structure similarity. ChemRICH then tests for significant enrichment of clusters using a Kolmogorov–Smirnov test.

### Training and validation of classifiers

Single-metabolite classification models and multi-metabolite models were trained on ADC1 plasma and serum samples separately. The analysis was stratified this way in order to determine which blood matrix would provide a better diagnostic tool for NSCLC adenocarcinoma. These models took as input the processed metabolite concentration profiles and classified each patient as adenocarcinoma or healthy. For single metabolite classifiers, logistic regression was used to estimate the predicted probability of cancer status given a covariate adjusted metabolite profile. A receiver operating characteristic (ROC) curve was then used to select the probability threshold affording the highest accuracy. Multi-metabolite classifiers were trained using four machine learning modeling methods: support vector machines [70, 71] (SVM), partial least squares linear discriminant analysis [72, 73] (PLS), random forests [74, 75] (RF), and extreme gradient boosted trees [76–78] (xgbTree). Models were trained using the R package *caret* [79]. To compare our clustering approach to more standard approaches, models were trained on all metabolites, significant metabolites, and each cluster of chemically similar significant metabolites.

Models were internally validated using leave-one-out cross-validation (LOOCV). A receiver operating characteristic (ROC) curve was then used to select the probability threshold affording the highest LOOCV accuracy. A grid search was performed on the machine learning model tuning parameters. The set of parameters resulting in the highest LOOCV accuracy were selected for each model.

While LOOCV was useful in the selection of the best performing metabolite classifiers, it was also necessary to externally validate their predictive power on an independent case study (ADC2). The models and probability thresholds selected according to LOOCV were used for classification on the external ADC2 set. At last, the best performing classifiers were determined according to both their internal and external classification accuracy.

## Conclusion

In this study, we demonstrated that clustering metabolites based on their structural similarity enabled us to identify modules of metabolites whose significant association with NSCLC adenocarcinoma status was detected in the presence of unwanted sources of variation. We also showed that these metabolites are linked by metabolic pathways potentially dysregulated by NSCLC adenocarcinoma.

This chemocentric analysis of metabolite profiles could facilitate the discovery of novel biomarkers and inferences regarding the health state or medical treatment outcomes for patients. Ultimately, the best performing classifier of patient cancer status using this transparent strategy was a serum multi-metabolite classifier (very high 84.9% overall accuracy), we thus demonstrate an improvement over alternative approaches. Metabolomics has proved to have widespread applicability to the search of clinical biomarkers, and predictive performance is of critical importance for any diagnostic biomarker. This approach could aid in identifying improved biomarkers in a number of metabolomics applications.

Importantly, because the grouped metabolites identified by these means were biochemically linked, the biological interpretability of the resulting model was maintained. As modern metabolomics platforms are increasingly able to detect and correctly assign chemical structures to a large number of metabolites, this standardized and automated procedure could have a broad applicability to investigators using metabolite profiles to establish reliable trait-metabolite relationships and predict phenotypic data.

One limitation of our approach is that metabolites that were detected by the untargeted metabolomics analysis, but did not have reliable structural annotations, could not be included in the modeling workflow. In the future, we plan to explore new ways to include those metabolites, especially by assigning them to the cluster(s) with most similar mass spectra.

This approach can also be used to identify structural features of metabolites that determine their mechanistic relationships with the trait of interest. In the future, we plan to build models that identify additional relationships between the trait and metabolite chemical descriptors. These models could be used to predict whether metabolites that were not detected by an untargeted study could still have a trait-metabolite association. This could provide leads for targeted validation to confirm these changes in metabolite concentrations and expand the analysis to other metabolites in related pathways. This approach could address existing challenges in untargeted metabolomics, such as the inferences about affected pathways that are often limited by the identification of only a few statistically significant metabolites.

## Additional files

**Additional file 1.** Supplementary results and figures.

**Additional file 2.** The scripts and additional data necessary to recreate our analyses.

## Data Availability

The datasets investigated in this study are freely available via the Metabolomics public repository (www.metabolomicsworkbench.org) under study numbers ST000385 and ST000386. All the scripts and additional data necessary to recreate our analyses are available in the Additional file 2 and at https://github.com/jrash/metabochem.

## Abbreviations

ADC: adenocarcinoma
ADP: adenosine diphosphate
ASW: average silhouette width
ATP: adenosine triphosphate
BSS: between group sum of squares
FDR: false discovery rate
GCTOF: gas chromatography time-of-flight
LOOCV: leave-one-out cross-validation
MACCS: molecular access system structural keys
MeSH: medical subject headings
NSCLC: non-small-cell lung cancer
PLS: partial least squares
PK/PD: pharmacokinetic/pharmacodynamic
RF: random forests
ROC: receiver operating characteristic
SVM: support vector machines
WSS: within group sum of squares
