# Supplementary material for: Cheminformatics approach to exploring and modeling trait-associated metabolite profiles
Source: J Cheminform. 2019 Jun 24;11:43. doi: 10.1186/s13321-019-0366-3 (PMC6591908; doi:10.1186/s13321-019-0366-3)
Supplement: Supplementary file 1 — Additional file 1. Supplementary results and figures. [file 13321_2019_366_MOESM1_ESM.docx]

**Cheminformatics Approach to Exploring and Modeling Trait-Associated Metabolite Profiles**

Jeremy R. Ash^1,2,3^, Melaine A. Kuenemann^1,3^, Daniel Rotroff^2,3^, Alison Motsinger-Reif^2,3^, and Denis Fourches^1,3,^*

*^1^ Department of Chemistry, North Carolina State University, Raleigh, NC, USA.*

*^2^ Department of Statistics, North Carolina State University, Raleigh, NC, USA.*

*^3^ Bioinformatics Research Center, North Carolina State University, Raleigh, NC, USA*

***To whom correspondence should be addressed. Email: [dfourch@ncsu.edu](mailto:dfourch@ncsu.edu)

**1 Supplementary Figures**


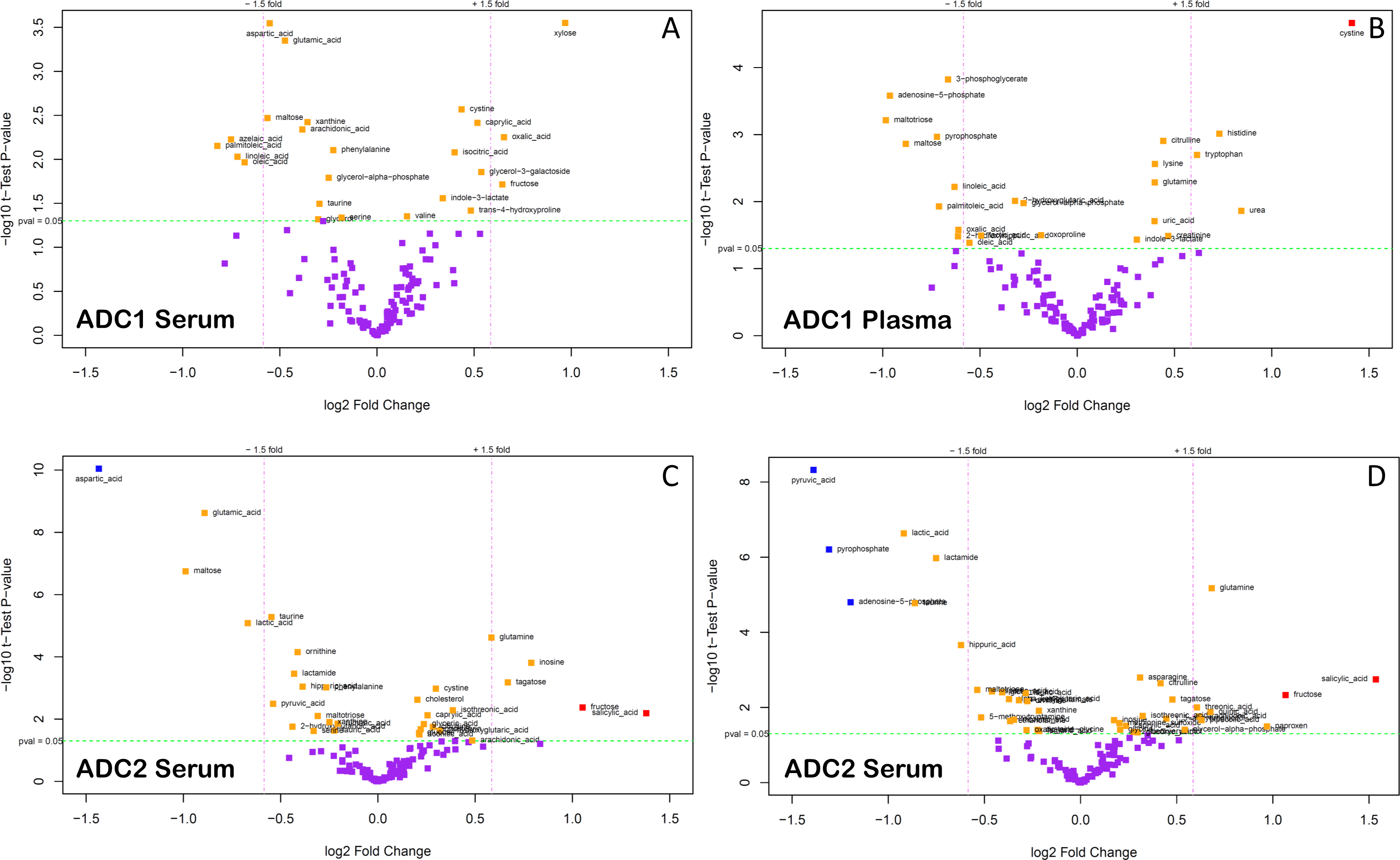


**Figure S1. Volcano plot showing significant difference in control versus cancer metabolite profiles.** Significant metabolites in (A) ADC1 (training) Serum, (B) ADC1 Plasma, (C) ADC2 (test) Serum, (D) ADC2 Plasma data sets. X-axis: log 2 fold change of control versus cancer metabolite relative abundance. Y-axis: raw p-values from paired t-tests for each metabolite. Significant metabolites (orange: raw p-value < .05) are labeled. Significant metabolites with log 2 fold change > 1 (red) and log 2 fold change < 1 (blue).


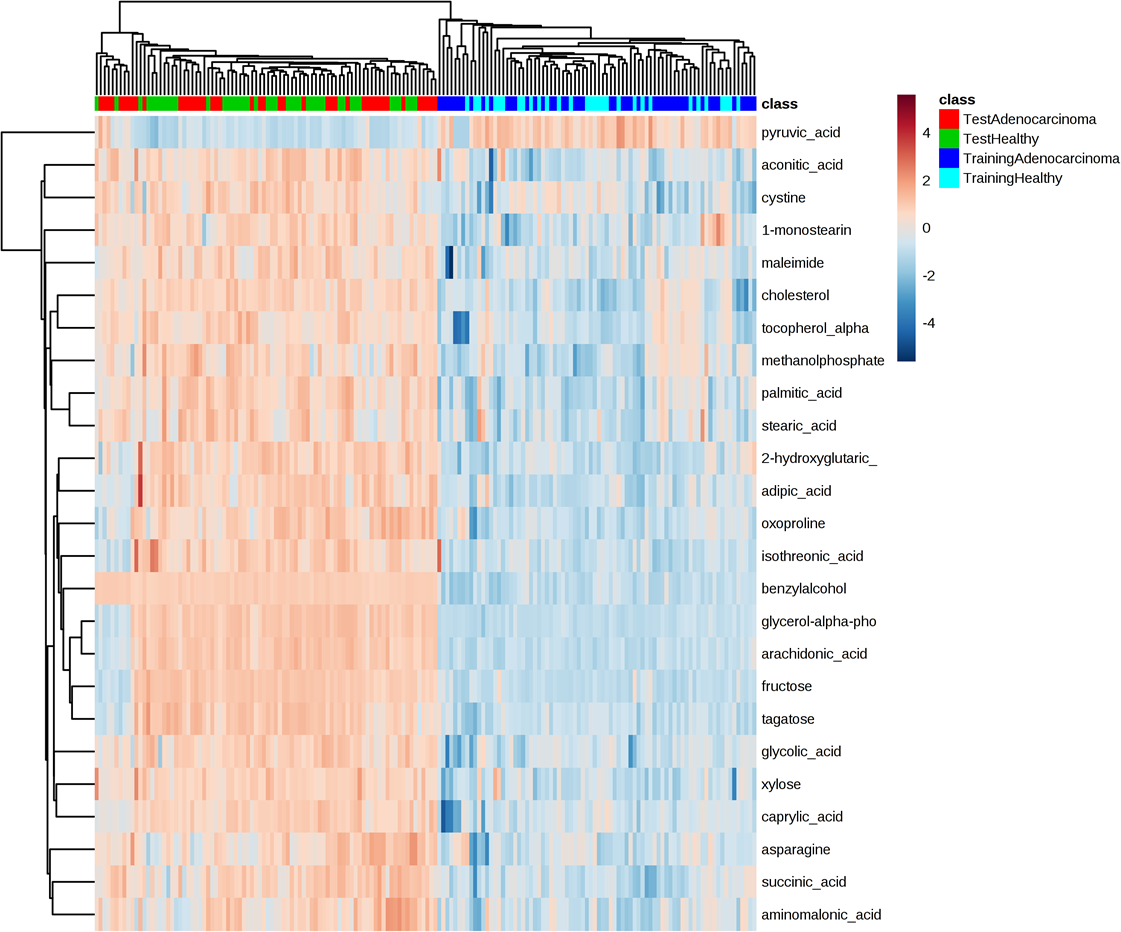


**Figure S2.** **25 serum metabolites with most significantly different mean log base 2 intensities (univariate T-test) between the ADC1 set and the ADC2 set prior to total quantity normalization.** Many metabolites that were not determined to be significantly associated with health state show either dramatic increases or decreases in mean intensity in ADC1 relative to ADC2.


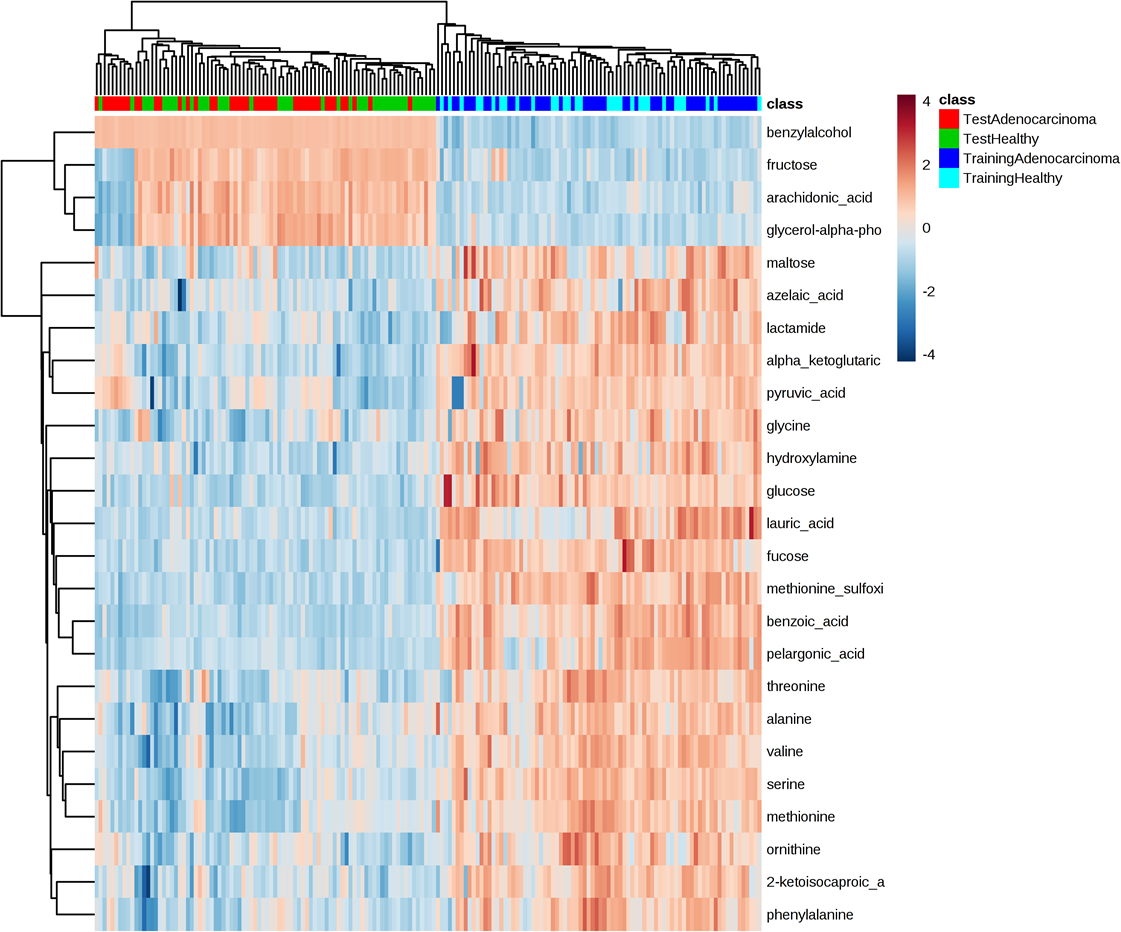


**Figure S3.** **25 plasma metabolites with most significantly different log base 2 intensities (univariate T-test) between the ADC1 set and the ADC2 set prior to total quantity normalization.** Many metabolites that were not determined to be significantly associated with health state show either dramatic increases or decreases in mean intensity in ADC1 relative to ADC2.


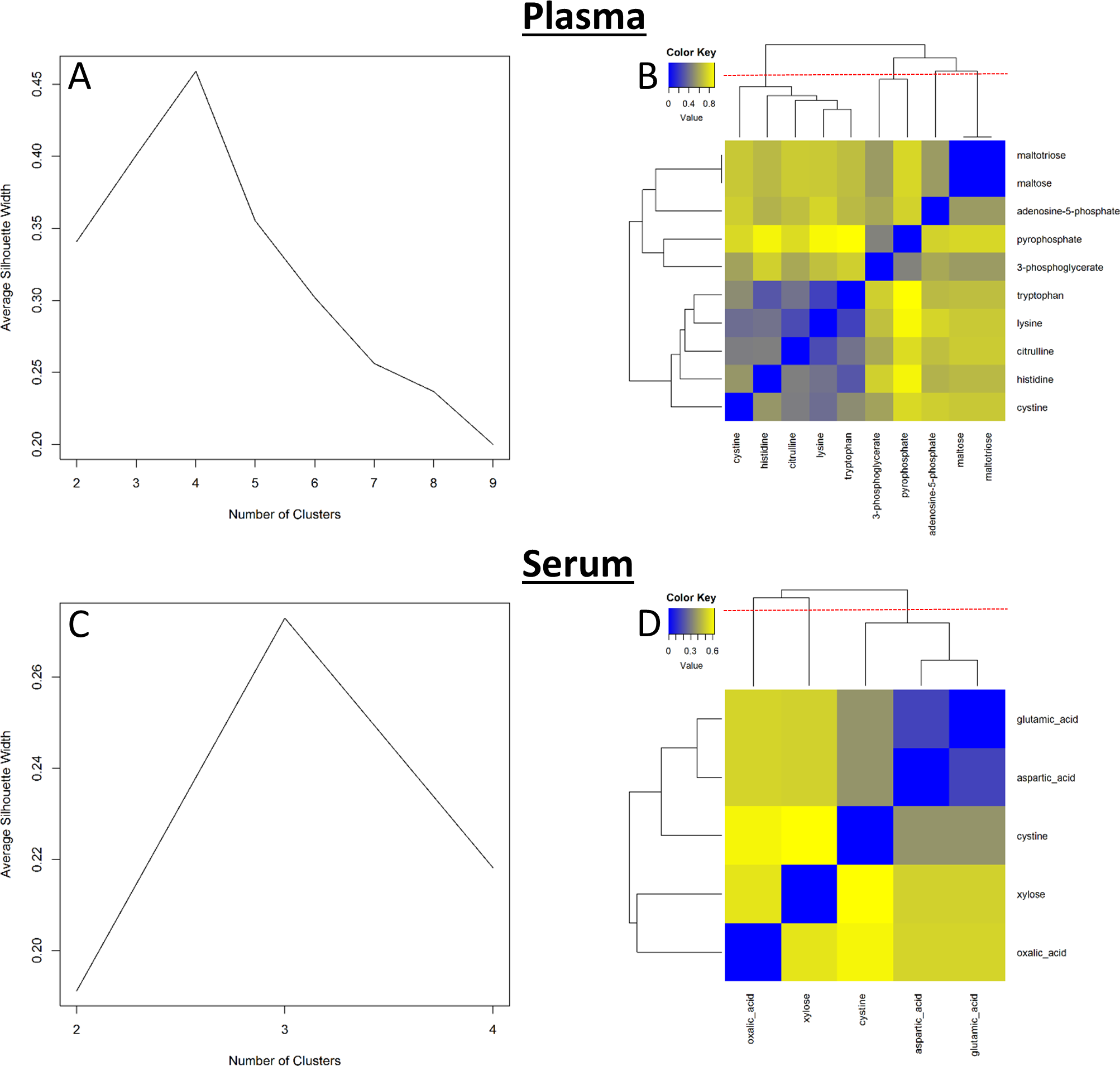


**Figure S4.** **Hierarchical clustering of chemical structures for metabolites significantly associated with cancer status**. (**A** and **C**) Average silhouette width (ASW) for all possible cuts in the cluster dendrogram. The cut with the maximum ASW was selected. Tanimoto distance between significant metabolite MACCS fingerprints in Plasma (**B**) and Serum (**D**).


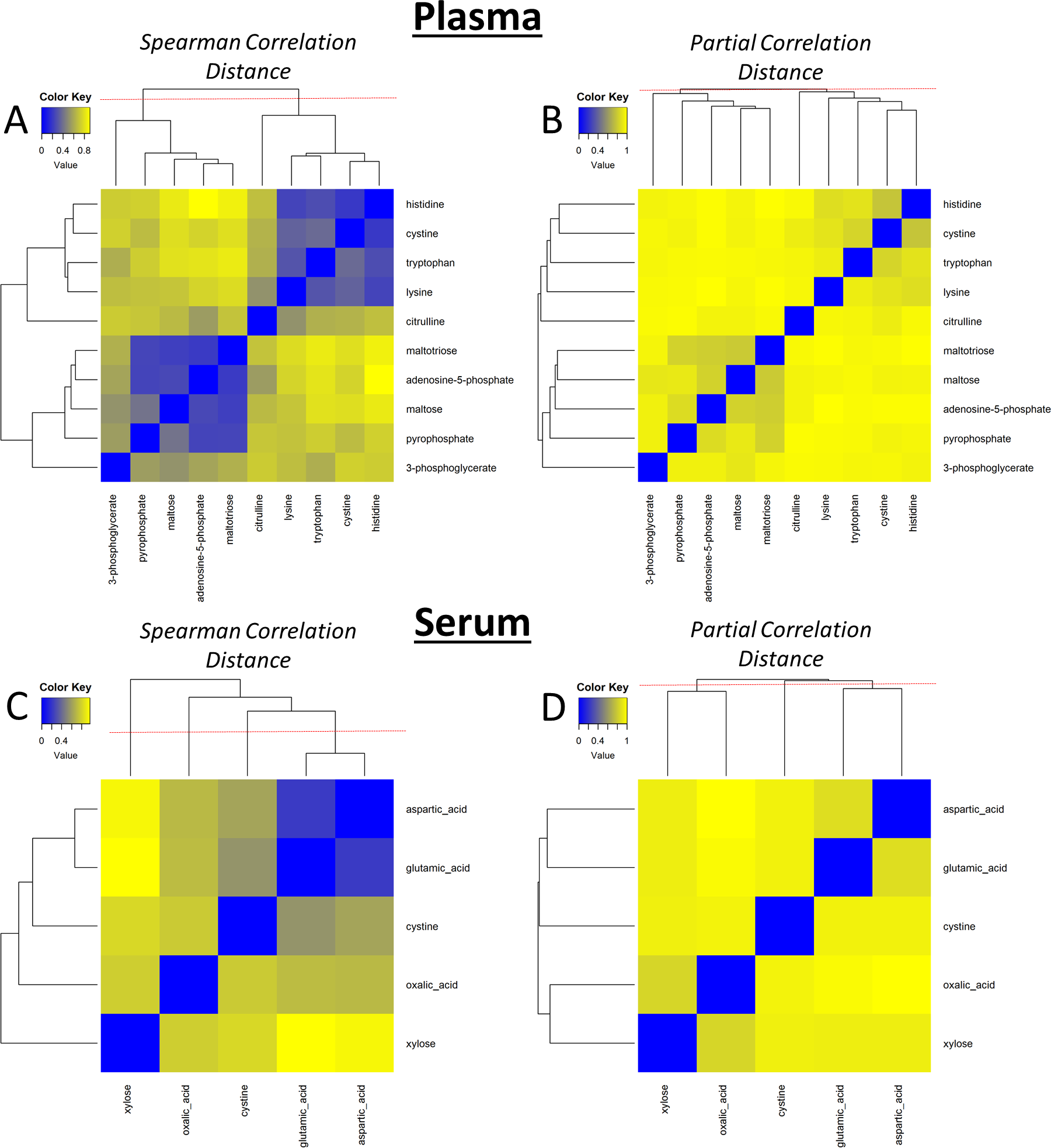


**Figure S5.** **Hierarchical clustering of patient profiles for metabolites significantly associated with cancer status.** Correlation based differences were computed between patient metabolite relative abundance profiles (1 – absolute correlation) using Spearman (**A**, **C**) and Partial (**B**, **D**) correlations.


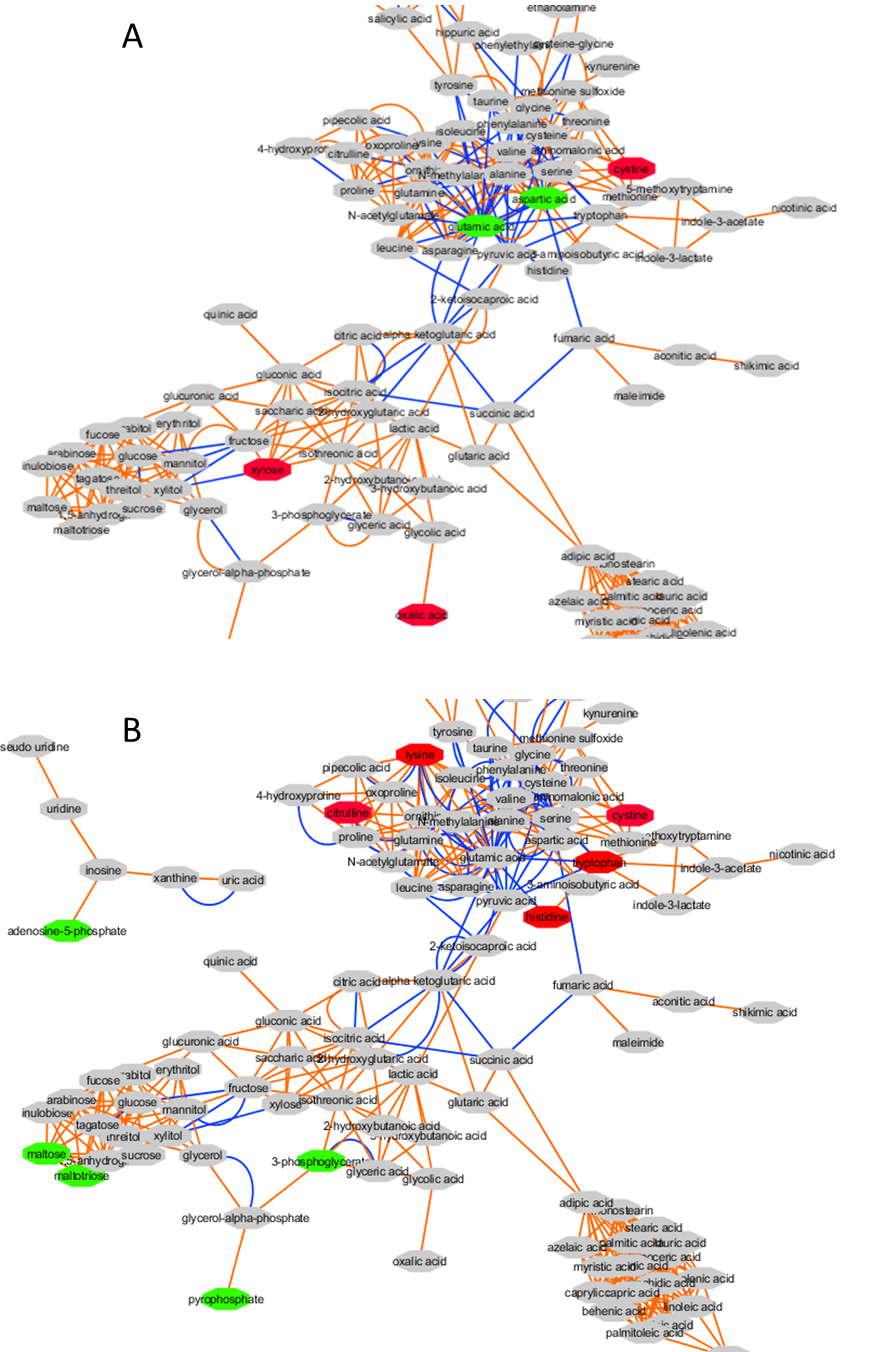


**Figure S6.** **MetaMapp network visualization of significant metabolites in serum (A) and plasma (B)**. Metabolites are connected with an orange edge if they are similar in chemical structure determined by a threshold on the Tanimoto similarity of their PubChem fingerprints. Metabolites are connected with a blue edge they can be interconverted by a single reaction in the KEGG reactant pair database.


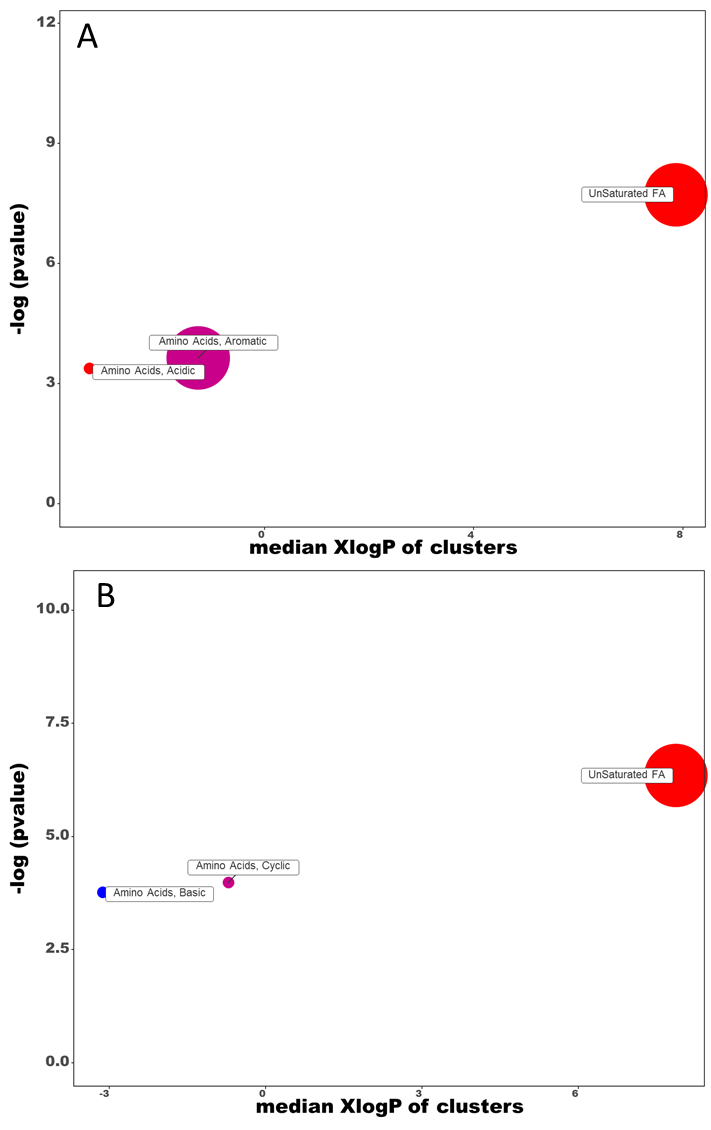


**Figure S7.** **Significantly enriched chemical clusters identified be ChemRICH in serum (A) and plasma (B).** A Kolmogorov–Smirnov test for set enrichment was performed using the unadjusted p-values from the serum and plasma metabolites differential analyses. An FDR threshold of .2 was used to determine significance. Significant serum clusters: unsaturated fatty acids (arachidonic acid, linoleic acid, linolenic acid, oleic acid, palmitoleic acid), aromatic amino acids (indole-3-acetate, indole-3-lactate, phenylalanine, tryptophan, tyrosine), and acidic amino acids (aspartic acid, glutamic acid, N-acetylglutamate). Significant plasma clusters: unsaturated fatty acids (arachidonic acid, linoleic acid, linolenic acid, oleic acid, palmitoleic acid), cyclic amino acids (4-hydroxyproline, histidine, oxoproline, proline), basic amino acids (asparagine, glutamine, lysine, ornithine)

**Plasma Serum**

|  | ***ADC1***  ***(Training)*** | ***ADC2***  ***(Test)*** | ***ADC1***  ***(Training)*** | ***ADC2***  ***(Test)*** |
| --- | --- | --- | --- | --- |
| **Total sample size** | 82 | 86 | 80 | 86 |
| Cancer cases | 51 | 43 | 49 | 43 |
| Healthy controls | 31 | 43 | 31 | 43 |
| **Gender (male/female)** | 52/30 | 46/40 | 51/29 | 46/40 |
| Cancer cases | 34/17 | 24/19 | 31/18 | 24/19 |
| Healthy controls | 18/13 | 22/21 | 20/11 | 22/21 |
| **Smoking Status (current/former)** | 24/58 | 31/55 | 24/56 | 31/55 |
| Cancer cases | 14/37 | 15/28 | 14/35 | 15/28 |
| Healthy controls | 10/21 | 16/27 | 10/21 | 16/27 |

**Supplementary Table S1.  Patient characteristics of the two cohorts.** Several of the factor variables (i.e., health state, smoking status, and gender) were unbalanced for some datasets.

***Single Metabolite Classifiers***

**ADC1 (Training) LOOCV ADC2 (Test) External Validation**

|  | Accuracy (%) | Sensitivity (%) | Specificity (%) | AUC | Accuracy (%) | Sensitivity (%) | Specificity (%) | AUC |
| --- | --- | --- | --- | --- | --- | --- | --- | --- |
| Aspartic Acid | 62.5 | 40.8 | 96.8 | 0.698 | 79.1 | 62.8 | 95.3 | 0.862 |
| Cystine | **70.0** | 75.5 | 61.3 | 0.685 | **55.8** | 76.7 | 34.9 | 0.677 |
| Glutamic Acid | 62.5 | 42.9 | 93.5 | 0.687 | 76.7 | 65.1 | 88.4 | 0.846 |
| Oxalic Acid | **70.0** | 83.7 | 48.4 | 0.650 | **57.0** | 88.4 | 25.6 | 0.649 |
| Xylose | 68.8 | 67.3 | 71.0 | 0.714 | 60.5 | 67.4 | 53.5 | 0.612 |

**Supplementary Table S2.  Performance measures for serum single metabolite models.** Best model accuracies according to LOOCV are bolded. Best model accuracies according to external validation accuracy are underlined.

***Multi-Metabolite Classifiers – All Metabolites***

ADC 1 (Training) LOOCV ADC2 (Test) External Validation

|  | Accuracy (%) | Sensitivity (%) | Specificity (%) | AUC | Accuracy (%) | Sensitivity (%) | Specificity (%) | AUC |
| --- | --- | --- | --- | --- | --- | --- | --- | --- |
| XgbTree | 73.8 | 87.8 | 51.6 | 0.700 | 61.6 | 83.7 | 39.5 | 0.784 |
| SVM | **81.3** | 91.8 | 64.5 | 0.795 | **50.0** | 93.0 | 7.00 | 0.718 |
| RF | 66.3 | 100.0 | 12.9 | 0.638 | 51.2 | 97.7 | 4.70 | 0.835 |
| PLS | 78.8 | 89.8 | 61.3 | 0.756 | 68.6 | 58.1 | 79.1 | 0.732 |

***Multi-Metabolite Classifiers – Significant Metabolites***

|  | Accuracy (%) | Sensitivity (%) | Specificity (%) | AUC | Accuracy (%) | Sensitivity (%) | Specificity (%) | AUC |
| --- | --- | --- | --- | --- | --- | --- | --- | --- |
| XgbTree | **83.8** | 83.7 | 83.9 | 0.812 | **59.3** | 18.6 | 100.0 | 0.807 |
| SVM | 81.3 | 87.8 | 71.0 | 0.854 | 69.8 | 39.5 | 100.0 | 0.865 |
| RF | 76.3 | 89.8 | 54.8 | 0.818 | 75.6 | 55.8 | 95.3 | 0.862 |
| PLS | 78.8 | 93.9 | 54.8 | 0.840 | 66.3 | 37.2 | 95.3 | 0.846 |

***Multi-Metabolite Classifiers – Clustered Metabolites***

|  | Accuracy (%) | Sensitivity (%) | Specificity (%) | AUC | Accuracy (%) | Sensitivity (%) | Specificity (%) | AUC |
| --- | --- | --- | --- | --- | --- | --- | --- | --- |
| Cluster 1^a^ XgbTree | 72.5 | 83.7 | 54.8 | 0.729 | 62.8 | 55.8 | 69.8 | 0.713 |
| Cluster 1 SVM | **76.3** | 77.6 | 74.2 | 0.751 | **84.9** | 72.1 | 97.7 | 0.856 |
| Cluster 1 RF | 61.3 | 100.0 | 0.00 | 0.594 | 50.0 | 100.0 | 0.00 | 0.766 |
| Cluster 1 PLS | 70.0 | 67.3 | 74.2 | 0.715 | 82.6 | 67.4 | 97.7 | 0.862 |

^a^ Aspartic Acid, Cystine, Glutamic Acid

**Supplementary Table S3.** **Performance measures for serum multi-metabolite classifiers.** Best model accuracies according to LOOCV are bolded. Best model accuracies according to external validation accuracy are underlined. Singleton clusters were not modeled.

***Single Metabolite Classifiers***

ADC 1 (Training) LOOCV ADC2 (Test) External Validation

|  | Accuracy (%) | Sensitivity (%) | Specificity (%) | AUC | Accuracy (%) | Sensitivity (%) | Specificity (%) | AUC |
| --- | --- | --- | --- | --- | --- | --- | --- | --- |
| 3-phosphoglycerate | 70.7 | 60.8 | 87.1 | 0.734 | 51.2 | 34.9 | 67.4 | 0.578 |
| Adenosine-5-phosphate | 67.1 | 58.8 | 80.6 | 0.703 | 73.3 | 65.1 | 81.4 | 0.789 |
| Citrulline | 64.6 | 51.0 | 87.1 | 0.674 | 66.3 | 55.8 | 76.7 | 0.710 |
| Cystine | 64.6 | 47.1 | 93.5 | 0.701 | 67.4 | 44.2 | 90.7 | 0.693 |
| Histidine | 65.9 | 68.6 | 61.3 | 0.650 | 52.3 | 81.4 | 23.3 | 0.611 |
| Lysine | 65.9 | 58.8 | 77.4 | 0.664 | 60.5 | 58.1 | 62.8 | 0.61 |
| Maltose | **74.4** | 82.4 | 61.3 | 0.701 | **57.0** | 62.8 | 51.2 | 0.607 |
| Maltotriose | 73.2 | 84.3 | 54.8 | 0.685 | 59.3 | 95.3 | 23.3 | 0.708 |
| Pyrophosphate | 69.5 | 66.7 | 74.2 | 0.703 | 76.7 | 67.4 | 86.0 | 0.811 |
| Tryptophan | 67.1 | 74.5 | 54.8 | 0.660 | 58.1 | 83.7 | 32.6 | 0.570 |

**Supplementary Table S4.** **Performance measures for plasma single metabolite models.** Best model accuracies according to LOOCV are bolded. Best model accuracies according to external validation accuracy are underlined.

***Multi-Metabolite Classifiers – All Metabolites***

ADC 1 (Training) LOOCV ADC2 (Test) External Validation

|  | Accuracy (%) | Sensitivity (%) | Specificity (%) | AUC | Accuracy (%) | Sensitivity (%) | Specificity (%) | AUC |
| --- | --- | --- | --- | --- | --- | --- | --- | --- |
| XgbTree | 76.8 | 82.4 | 67.7 | 0.760 | 58.1 | 79.1 | 37.2 | 0.652 |
| SVM | **79.3** | 92.2 | 58.1 | 0.801 | **69.8** | 69.8 | 69.8 | 0.721 |
| RF | 69.5 | 88.2 | 38.7 | 0.712 | 50.0 | 95.3 | 4.70 | 0.789 |
| PLS | 76.8 | 92.2 | 51.6 | 0.769 | 59.3 | 30.2 | 88.4 | 0.728 |

***Multi-Metabolite Classifiers – Significant Metabolites***

|  | Accuracy (%) | Sensitivity (%) | Specificity (%) | AUC | Accuracy (%) | Sensitivity (%) | Specificity (%) | AUC |
| --- | --- | --- | --- | --- | --- | --- | --- | --- |
| XgbTree | 76.8 | 88.2 | 58.1 | 0.731 | 54.7 | 76.7 | 32.6 | 0.554 |
| SVM | **78.0** | 74.5 | 83.9 | 0.791 | **69.8** | 44.2 | 95.3 | 0.763 |
| RF | 73.2 | 78.4 | 64.5 | 0.736 | 66.3 | 69.8 | 62.8 | 0.738 |
| PLS | 76.8 | 74.5 | 80.6 | 0.796 | 70.9 | 46.5 | 95.3 | 0.792 |

***Multi-Metabolite Classifiers – Clustered Metabolites***

|  | Accuracy (%) | Sensitivity (%) | Specificity (%) | AUC | Accuracy (%) | Sensitivity (%) | Specificity (%) | AUC |
| --- | --- | --- | --- | --- | --- | --- | --- | --- |
| Cluster 1^a^ XgbTree | 79.3 | 86.3 | 67.7 | 0.752 | 70.9 | 65.1 | 76.7 | 0.708 |
| Cluster 1 SVM | **80.5** | 86.3 | 71.0 | 0.713 | **70.9** | 72.1 | 69.8 | 0.675 |
| Cluster 1 RF | 70.7 | 80.4 | 54.8 | 0.719 | 69.8 | 65.1 | 74.4 | 0.745 |
| Cluster 1 PLS | 73.2 | 94.1 | 38.7 | 0.748 | 69.8 | 67.4 | 72.1 | 0.751 |
| Cluster 2^b^ XgbTree | 69.5 | 86.3 | 41.9 | 0.634 | 52.3 | 95.3 | 9.3 | 0.538 |
| Cluster 2 SVM | 70.7 | 70.6 | 71.0 | 0.681 | 47.7 | 95.3 | 0.00 | 0.708 |
| Cluster 2 RF | 64.6 | 56.9 | 77.4 | 0.617 | 54.7 | 86.0 | 23.3 | 0.643 |
| Cluster 2 PLS | 69.5 | 78.4 | 54.8 | 0.684 | 47.7 | 93.0 | 2.30 | 0.713 |
| Cluster 3^c^ XgbTree | 79.3 | 92.2 | 58.1 | 0.694 | 39.5 | 65.1 | 14.0 | 0.599 |
| Cluster 3 SVM | 78.0 | 88.2 | 61.3 | 0.636 | 43.0 | 48.8 | 37.2 | 0.631 |
| Cluster 3 RF | 73.2 | 94.1 | 38.7 | 0.645 | 43.0 | 76.7 | 9.30 | 0.580 |
| Cluster 3 PLS | 72.0 | 82.4 | 54.8 | 0.698 | 58.1 | 23.3 | 93.0 | 0.678 |

^a^ 3-phosphoglycerate, Pyrophosphate

^b^ Citrulline, Cysteine, Histidine, Lysine

^c^ Maltose, Maltotriose

**Supplementary Table S5.** **Performance measures for plasma multi-metabolite classifiers.** Best model accuracies according to LOOCV are bolded. Best model accuracies according to external validation accuracy are underlined. Singleton clusters were not modeled.

**Supplementary Results and Discussion**

We compared our chemical clustering approach to two other methods that could be used to group metabolites based on chemical structure similarity: MetaMapp [25] and ChemRICH [21]. We wanted to determine if these methods could be used to derive similar multi-metabolite classifiers compared to our approach. All of the metabolites detected in serum and plasma were used to infer the MetaMapp networks (**Supplementary Figure 6**), as this is the typical procedure for detecting modules within these networks.

The grouping of serum metabolites that led to our best performing classifier might have been identified in the MetaMapp network. One method for building multi-metabolite classifiers based on these networks might be to group significant metabolites based on the apparent modularity in the network. Using this method, one may have chosen to group aspartic acid, glutamic acid, and cystine. However, one disadvantage of this network approach is that there is no objective method for determining clusters. It is unclear whether cystine should be grouped with aspartic acid and glutamic acid. There is a subgraph connecting all three metabolites in the chemical similarity network, but not in the reaction pair network. Forming two groups by separating cystine from aspartic acid and glutamic acid resulted in classifiers with worse prediction performances in both ADC1 and ADC2.

In plasma, one would likely not group together pyrophosphate and 3-phosphoclycerate as there is no edge connecting these two metabolites together. In fact, separating these metabolites results in classifiers with suboptimal prediction performance. This highlighted another disadvantage of this method: the chemical similarity network is constructed by using a hard .7 threshold on the Tanimoto similarity of compounds. By using the ASW to find a natural clustering of the significant metabolites, our approach selects a threshold on the Tanimoto similarity in a more data-driven, dataset-specific manner.

When the BH adjusted p-values were provided to ChemRICH, no chemical clusters were found to be significantly enriched. Instead, we provided ChemRICH with unadjusted p-values and found three significantly enriched clusters in serum and plasma (**Supplementary Figure 7**). Since unadjusted p-values were used, several of these significantly enriched clusters are likely false positives and would not lead to successful classification models. We could construct multi-metabolite classifiers by using the enriched clusters to group the significant metabolites (according to adjusted p-values). This procedure would group together aspartic acid and glutamic acid in serum, but would exclude cystine. ChemRICH also did not group together pyrophosphate and 3-phosphoglycerate. ChemRICH uses MeSH ontologies to group together metabolites and only groups together metabolites based on chemical structure when no ontology annotation can be obtained. This procedure has its advantages, as the clusters obtained by this method have clear annotations. However, our method, which solely uses chemical structure to group similar metabolites together, identified clusters that differed from the MeSH ontology annotations and led to classifiers with improved prediction performances. A functional relationship between the metabolites within our classifiers was able to be established once the most predictive models were identified. Overall, our results suggest that MetaMapp and ChemRICH could be used in the chemical structure clustering step of our workflow to construct highly predictive multi-metabolite classifiers. However, we believe that our clustering procedure has particular advantages that make it more suitable for a predictive model workflow.
