# Supplementary material for: Cheminformatics approach to exploring and modeling trait-associated metabolite profiles
Source: J Cheminform. 2019 Jun 24;11:43. doi: 10.1186/s13321-019-0366-3 (PMC6591908; doi:10.1186/s13321-019-0366-3)
Supplement: Supplementary file 2 — Additional file 2. The scripts and additional data necessary to recreate our analyses. [file 13321_2019_366_MOESM2_ESM.zip › metabochem-master/analyses/differential_analysis.pdf]

Significant Metabolites Analysis

Jeremy Ash

May 22, 2017

Contents

1 Training Set **1**
1.1 Patient characteristic table 3
1.2 T-Tests for Significant differences 4
1.3 Non-parameteric approach 10

2 Test Set **16**
2.1 Patient characteristic table 18
2.2 T-Tests for Significant differences 19
2.3 Non-parameteric approach 25

1 Training Set

```
rm(list = ls())
sdfset <- read.SDFset("common_test_training_molecule-v2.sdf")

# test for significance with only compounds that have chemical structures
f1 <- read.delim(file = "sample_factors_training.txt", header = T, row.names = 1)
f2 <- read.csv(file = "sample_metabolites_training_excol_fix.csv", header = T, row.names = 1)

# removing the observations with no factor levels
f1 <- na.omit(f1)
rownames(f1) <- f1$Sample.name

# create patient identifier column
f1$Subject_name <- gsub(" Plasma| Serum", "", f1$Subject_name, perl = T)

f1 <- f1[, -2]
colnames(f1) <- c("Patient","Organ", "Health_State", "Smoking_Status", "Gender")
head(f1)
```

```
##                   Patient  Organ   Health_State Smoking_Status Gender
## 130729dlvsa13_1       7 Plasma Adenocarcinoma        Current      F
## 130729dlvsa17_1       9 Plasma Adenocarcinoma        Current      F
## 130729dlvsa28_1      15 Plasma Adenocarcinoma        Current      F
## 130729dlvsa46_3      24 Plasma Adenocarcinoma        Current      F
## 130729dlvsa48_2      25 Plasma Adenocarcinoma        Current      F
## 130730dlvsa28_1      41 Plasma Adenocarcinoma        Current      F
```

```
f2 <- t(f2)

# save this processed data frame so you can try different processing
before.process <- f2
```

```r
# save.image("training_set.rda")

# two metabolites with missing values, use imputation, take half the minimum
# of that metabolite's value
# also, perform log base 2 transformation.  I cannot for the life of me
# figure out how they did their normalization
# lactic acid had some zero values? impute those too
f2 <- apply(f2, 2, function(x) {
  x[is.na(x)] <- .5*min(na.omit(x))
  x[x == 0] <- .5*min(na.omit(x[x != 0]))
  x
})

# # not necessary to do total quantity normalization
# # pvalues dont change see below
for(i in 1:nrow(f2)){
  f2[i, ] <- f2[i, ]/sum(f2[i, ])
}

# log transformation
f2 <- log(f2, base = 2)

summary(f2[, 1:6])
```

```
##  1,5-anhydroglucitol 1-monopalmitin   1-monostearin
##  Min.   :-13.520     Min.   :-16.80   Min.   :-16.92
##  1st Qu.: -7.594     1st Qu.:-14.22   1st Qu.:-15.08
##  Median : -7.167     Median :-13.83   Median :-14.62
##  Mean   : -7.446     Mean   :-13.82   Mean   :-14.54
##  3rd Qu.: -6.763     3rd Qu.:-13.45   3rd Qu.:-14.09
##  Max.   : -5.797     Max.   :-10.68   Max.   :-11.81
##  2,3,5-trihydroxypyrazine 2,3-dihydroxybutanoic_acid 2-aminoadipic_acid
##  Min.   :-16.61           Min.   :-16.04            Min.   :-17.70
##  1st Qu.:-15.02           1st Qu.:-14.18            1st Qu.:-14.68
##  Median :-14.63           Median :-13.64            Median :-14.26
##  Mean   :-14.64           Mean   :-13.70            Mean   :-14.32
##  3rd Qu.:-14.22           3rd Qu.:-13.23            3rd Qu.:-13.85
##  Max.   :-13.14           Max.   :-12.41            Max.   :-12.66
```

```r
# fix compound names so that they are the same
# format as the file provided by Melaine
ids <- sdfid(sdfset)
ids.new <- gsub(" |,","_",ids, perl = T)
colnames(f2) <- gsub(" |,","_", colnames(f2), perl = T)

# 130 compounds provided
sum(ids.new %in% colnames(f2))
```

```
## [1] 130
```

```r
dim(f2)
```

```
## [1] 180 176
```

```r
f2 <- f2[, colnames(f2) %in% ids.new]
orig.mets <- colnames(f2)[colnames(f2) %in% ids.new]
```

```r
rownames(f2) <- sub("X", "", row.names(f2))
d <- merge(f1, f2, by.x= "row.names", by.y = "row.names")

d$Organ <- factor(d$Organ)

#replacing mispelled adenocarcinoma and "Adenosquamous" with Adenocarcinoma
d$Health_State <- gsub("Adenocarcnoma|Adenosquamous", "Adenocarcinoma", d$Health_State)
row.names(d) <- d$Row.names
d$Row.names <- NULL
save.image("training_set_tq_nonormalize.rda")
```

1.1 Patient characteristic table

```r
table(d$Organ, d$Health_State)
```

```
##
##          Adenocarcinoma Healthy
##   Plasma             51      31
##   Serum              49      31
```

```r
table(d$Organ, d$Smoking_Status)
```

```
##
##          Current Former
##   Plasma      24     58
##   Serum       24     56
```

```r
table(d$Organ, d$Smoking_Status, d$Health_State)
```

```
## , ,  = Adenocarcinoma
##
##
##          Current Former
##   Plasma      14     37
##   Serum       14     35
##
## , ,  = Healthy
##
##
##          Current Former
##   Plasma      10     21
##   Serum       10     21
```

```r
table(d$Organ, d$Gender)
```

```
##
##           F  M
##   Plasma 52 30
##   Serum  51 29
```

```r
table(d$Organ, d$Gender, d$Health_State)
```

```
## , ,  = Adenocarcinoma
##
##
```

```
##           F  M
##   Plasma 34 17
##   Serum  31 18
##
## , ,  = Healthy
##
##
##           F  M
##   Plasma 18 13
##   Serum  20 11
```

1.2 T-Tests for Significant differences

```
vars = colnames(d)[6:ncol(d)]
varNum <- length(vars)
pkimodels <- vector("list", (varNum))
pkimodelspvals <- vector("list", (varNum))
pkimodelseffect <- vector("list", (varNum))
pkimodelsmean <- vector("list", (varNum))

#----controling for all factors, including organ

for (i in 1:(varNum)){
  lmfit <- lm(d[,i+5]~ Organ + Health_State + Smoking_Status + Gender, data = d)
  pkimodels[[i]] <- lmfit
  #pvalue for regressing each variable in df on AC50
  pkimodelspvals[[i]] <- Anova(lmfit, type = "III")$`Pr(>F)`[3]

  pkimodelseffect[[i]] <- log(mean(2^(d[d$Health_State == "Adenocarcinoma",i+5]))/mean(2^(d[d$Health_Sta
  # mins[[i]] <- min(d[,220])
}

pki_ps = unlist(pkimodelspvals)
pki_ps <- p.adjust(pki_ps, method = "BH")

pki_effect = unlist(pkimodelseffect)
plot(pki_effect, -log(pki_ps, base = 10))
```

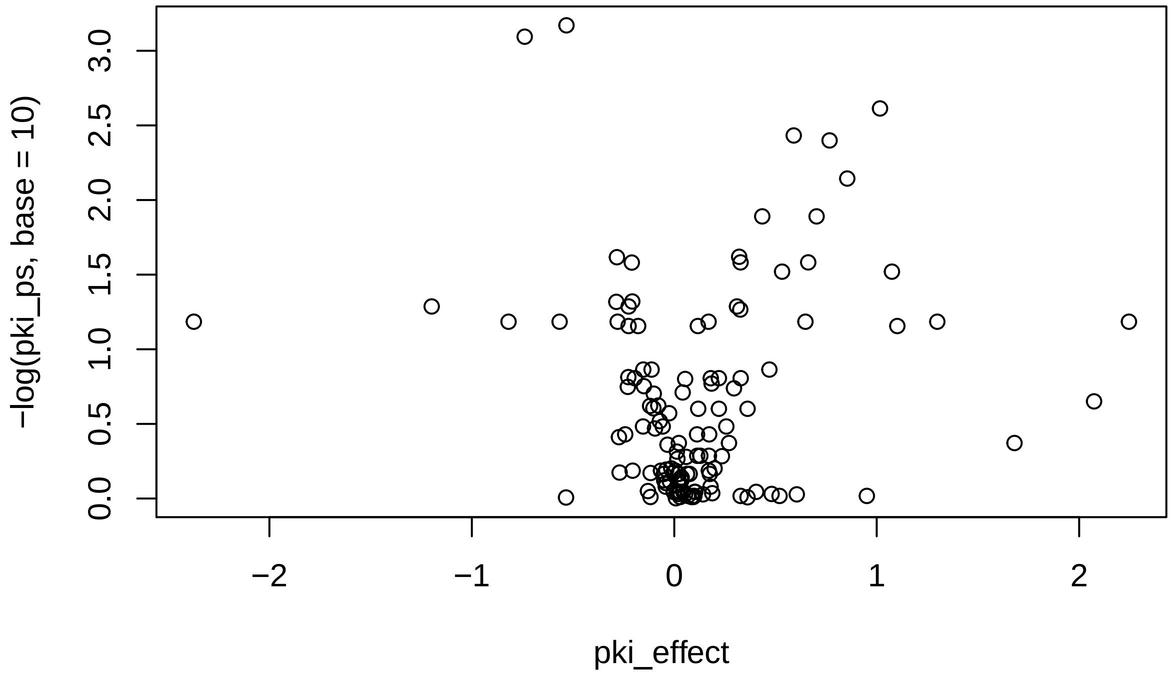


```
log2FoldChange <- pki_effect

univariate_res_control = data.frame(variables = vars, pvalues = pki_ps, log2FoldChange = log2FoldChange)
univariate_res_control$significance <- univariate_res_control$pvalues < .05
sig_no_block <- univariate_res_control$pvalues < .05
write.csv(univariate_res_control, "healthstate_anova_wsig_control_training.txt", row.names = F)

#-----Using patient as blocking factor

for (i in 1:(varNum)){
  lmfit <- lmer(d[,i+5]~ Organ + Health_State + Smoking_Status + Gender + (1|Patient), data = d)
  pkimodels[[i]] <- lmfit
  #pvalue for regressing each variable in df on AC50
  pkimodelspvals[[i]] <- Anova(lmfit, type = "III")$`Pr(>Chisq)`[3]

  pkimodelseffect[[i]] <- mean(2^(d[d$Health_State == "Adenocarcinoma",i+5]))/mean(2^(d[d$Health_State =
  # mins[[i]] <- min(d[,220])
}

pki_effect = unlist(pkimodelseffect)
log2FoldChange <- pki_effect

pki_ps_raw = unlist(pkimodelspvals)
plot(log(pki_effect, base = 2), -log(pki_ps_raw, base = 10))
```

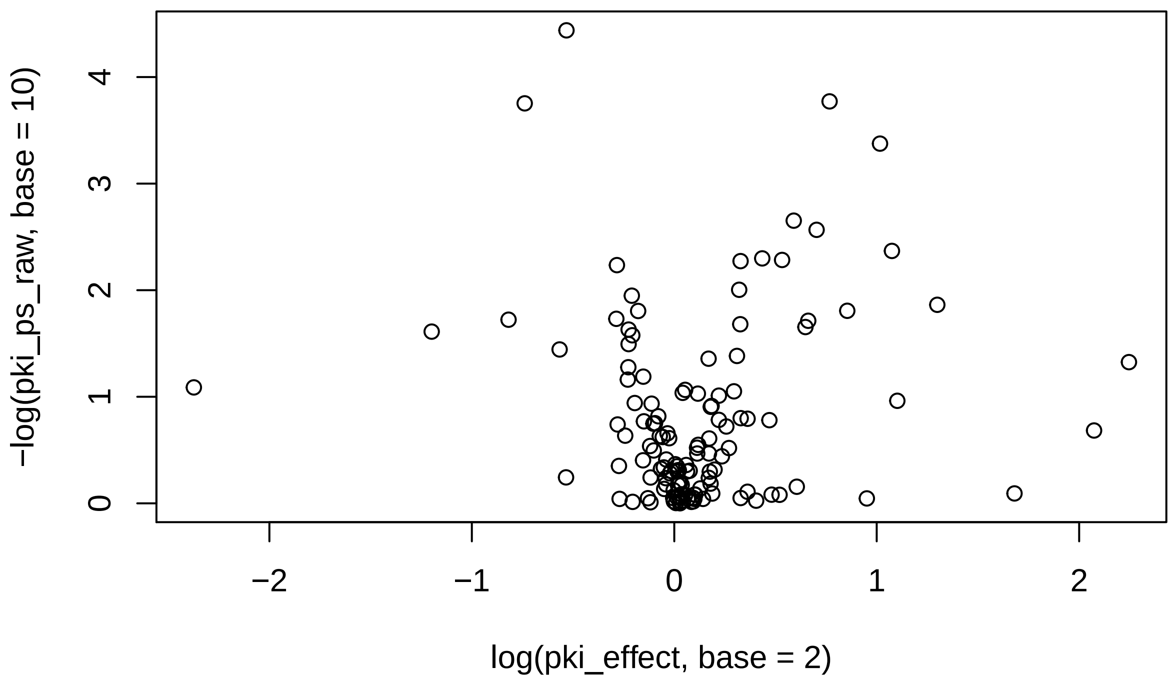


```r
pki_ps_adj <- p.adjust(pki_ps_raw, method = "BH")
plot(log(pki_effect, base = 2), -log(pki_ps_adj, base = 10))
```

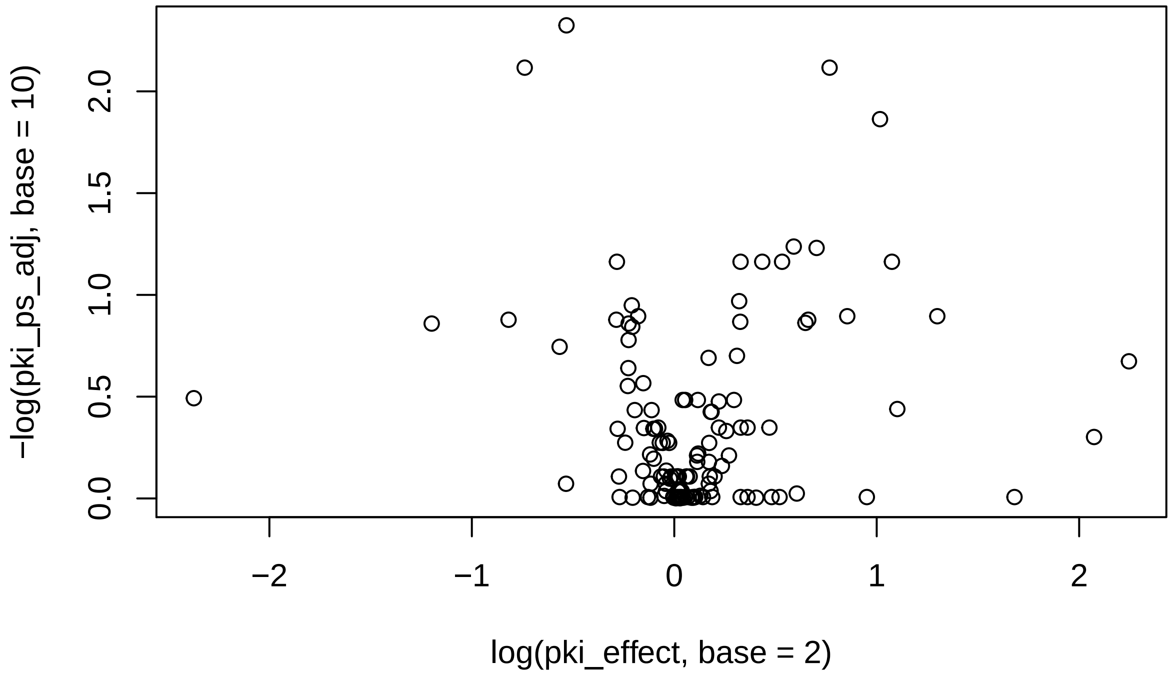


```
univariate_res_control = data.frame(variables = vars, pvalues = pki_ps_raw,
                                    adj_pvalues = pki_ps_adj,
                                    FoldChange = log2FoldChange)
univariate_res_control$significance_raw <- univariate_res_control$pvalues < .05
univariate_res_control$significance_adj <- univariate_res_control$adj_pvalues < .05
write.csv(univariate_res_control, "healthstate_anova_wsig_control_training_block.txt", row.names = F)


#----only analyze the samples collected from Serum
d_serum <- d[d$Organ == "Serum", ]
#----controling for all factors
for (i in 1:(varNum)){
  lmfit <- lm(d_serum[,i+5]~ Health_State + Smoking_Status + Gender, data = d_serum)
  pkimodels[[i]] <- lmfit
  #pvalue for regressing each variable in df on AC50
  pkimodelspvals[[i]] <- Anova(lmfit, type = "III")$`Pr(>F)`[3]

  pkimodelseffect[[i]] <-
    log(mean(2^(d_serum[d_serum$Health_State == "Adenocarcinoma",i+5]))/mean(2^(d_serum[d_serum$Health_S
  pkimodelsmean[[i]] <- mean(d_serum[d_serum$Health_State == "Adenocarcinoma",i+5])
}

pki_ps = unlist(pkimodelspvals)
pki_ps <- p.adjust(pki_ps, method = "BH")

pki_effect = unlist(pkimodelseffect)
```

```r
plot(pki_effect, -log(pki_ps, base = 10))
```

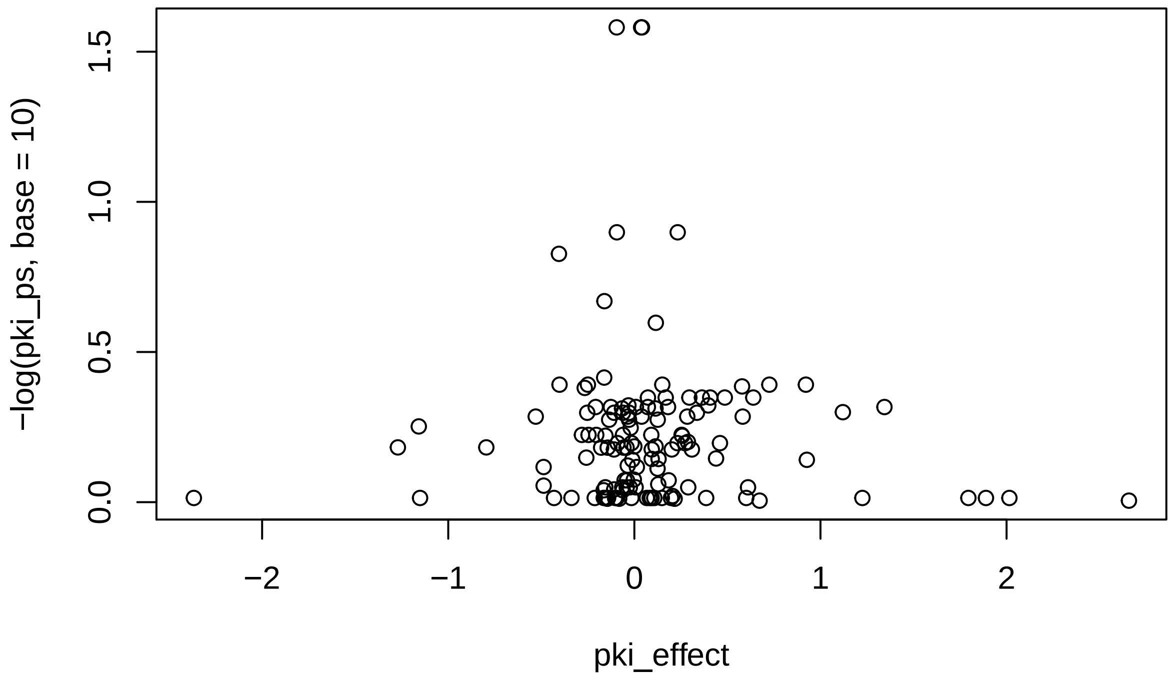


```r
log2FoldChange <- pki_effect
pkimodelsmean <- unlist(pkimodelsmean)

univariate_res_control = data.frame(variables = vars, pvalues = pki_ps,
                                    log2FoldChange = log2FoldChange,
                                    pkimodelsmean = pkimodelsmean)
univariate_res_control$significance <- univariate_res_control$pvalues < .05

sum(univariate_res_control$significance)
```

```
## [1] 3
```

```r
write.csv(univariate_res_control, "healthstate_anova_wsig_control_training_serum.txt", row.names = F)

#----only analyze the samples collected from plasma
d_plasma <- d[d$Organ == "Plasma", ]

#----controling for all factors
for (i in 1:(varNum)){
  lmfit <- lm(d_plasma[,i+5]~ Health_State + Smoking_Status + Gender, data = d_plasma)
  pkimodels[[i]] <- lmfit
  #pvalue for regressing each variable in df on AC50
  pkimodelspvals[[i]] <- Anova(lmfit, type = "III")$`Pr(>F)`[3]

  pkimodelseffect[[i]] <-
```

```
    log(mean(2^(d_plasma[d_plasma$Health_State == "Adenocarcinoma",i+5]))/
          mean(2^(d_plasma[d_plasma$Health_State == "Healthy",i+5])), base=2)
  pkimodelsmean[[i]] <- mean(d_plasma[d_plasma$Health_State == "Adenocarcinoma",i+5])

}

pki_ps = unlist(pkimodelspvals)
pki_ps <- p.adjust(pki_ps, method = "BH")

pki_effect = unlist(pkimodelseffect)
plot(pki_effect, -log(pki_ps, base = 10))
```

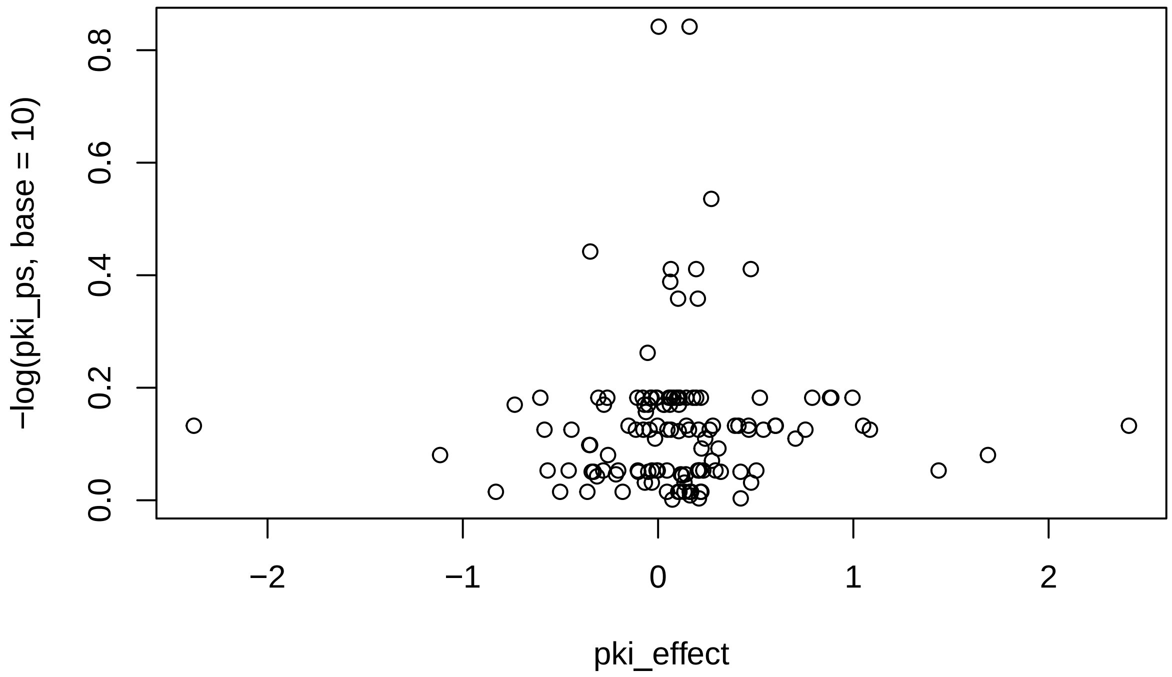


```
log2FoldChange <- pki_effect
pkimodelsmean <- unlist(pkimodelsmean)

univariate_res_control = data.frame(variables = vars, pvalues = pki_ps,
                                    log2FoldChange = log2FoldChange,
                                    pkimodelsmean = pkimodelsmean)
univariate_res_control$significance <- univariate_res_control$pvalues < .05
sum(univariate_res_control$significance)
```

```
## [1] 0
```

```
write.csv(univariate_res_control, "healthstate_anova_wsig_control_training_plasma.txt", row.names = F)
```

1.3 Non-parameteric approach

```
#----only analyze the samples collected from Serum
d_serum <- d[d$Organ == "Serum", ]
#----controling for all factors
for (i in 1:(varNum)){
  lmfit <- lm(d_serum[,i+5]~ Smoking_Status + Gender, data = d_serum)
  pkimodelspvals[[i]] <-   permTS(lmfit$residuals ~ Health_State, data = d_serum,
                                  alternative="two.sided", method="exact.mc",
                                  control=permControl(nmc=10^5))$p.value
  # Switched to reporting FC instead of logFC here so that can get right input for volcano plot
  # on metabolomics workbench
  pkimodelseffect[[i]] <-
    mean(2^(d_serum[d_serum$Health_State == "Adenocarcinoma",i+5]))/mean(2^(d_serum[d_serum$Health_Stat
  pkimodelsmean[[i]] <- mean(d_plasma[d_plasma$Health_State == "Adenocarcinoma",i+5])
}

pki_effect = unlist(pkimodelseffect)
FoldChange <- pki_effect
pkimodelsmean <- unlist(pkimodelsmean)

pki_ps_raw = unlist(pkimodelspvals)
plot(log(pki_effect, base = 2), -log(pki_ps_raw, base = 10))
```

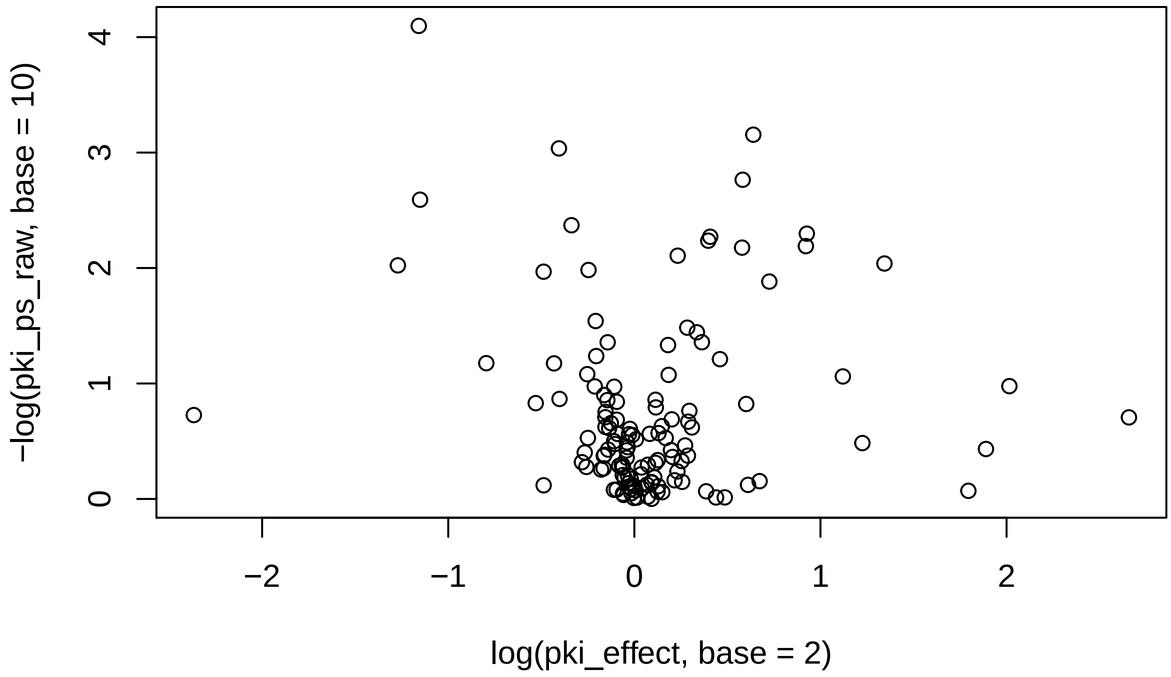


```
pki_ps_adj <- p.adjust(pki_ps_raw, method = "BH")
plot(log(pki_effect, base = 2), -log(pki_ps_adj, base = 10))
```

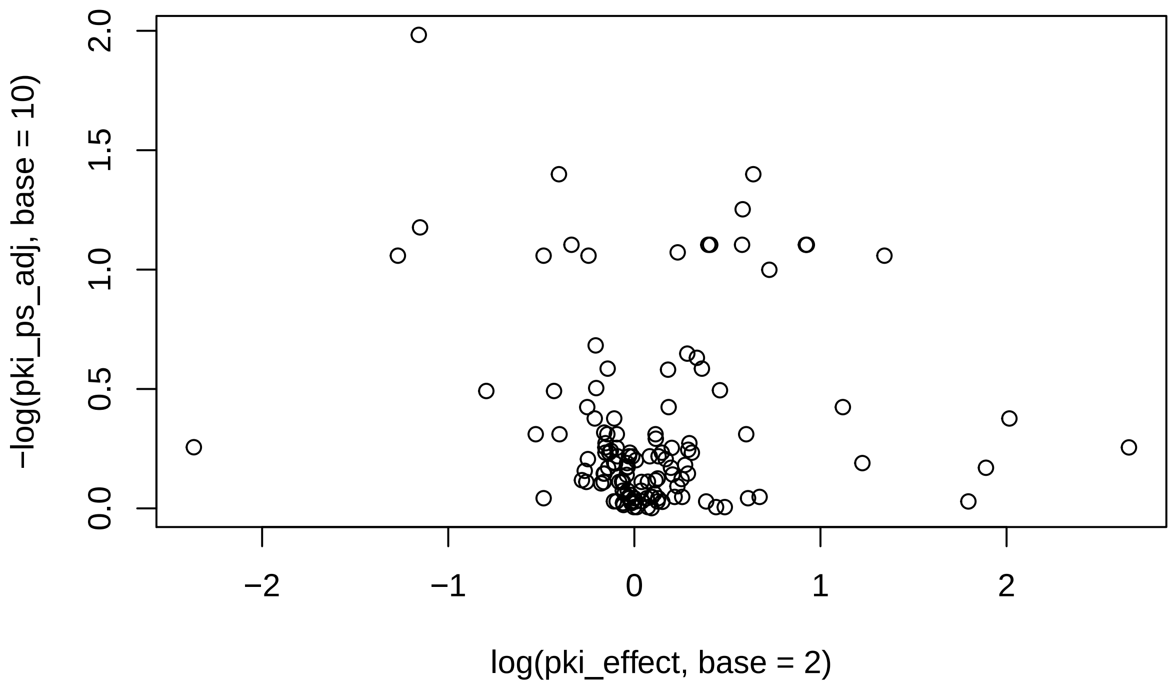


```
univariate_res_control = data.frame(variables = vars, pvalues = pki_ps_raw,
                                    adj_pvalues = pki_ps_adj,
                                    FoldChange = FoldChange,
                                    pkimodelsmean = pkimodelsmean)
univariate_res_control$significance_raw <- univariate_res_control$pvalues < .05
univariate_res_control$significance_adj <- univariate_res_control$adj_pvalues < .05
write.csv(univariate_res_control, "healthstate_anova_wsig_control_training_serum_nonpara.txt", row.names

#----only analyze the samples collected from Plasma
d_plasma <- d[d$Organ == "Plasma", ]

#----controling for all factors
for (i in 1:(varNum)){
  lmfit <- lm(d_plasma[,i+5]~ Smoking_Status + Gender, data = d_plasma)
  pkimodelspvals[[i]] <-   permTS(lmfit$residuals ~ Health_State, data = d_plasma,
                                  alternative="two.sided", method="exact.mc",
                                  control=permControl(nmc=10^5))$p.value

  pkimodelseffect[[i]] <-
    mean(2^(d_plasma[d_plasma$Health_State == "Adenocarcinoma",i+5]))/
          mean(2^(d_plasma[d_plasma$Health_State == "Healthy",i+5]))
  pkimodelsmean[[i]] <- mean(d_plasma[d_plasma$Health_State == "Adenocarcinoma",i+5])

}

pki_effect = unlist(pkimodelseffect)
```

```r
FoldChange <- pki_effect
pkimodelsmean <- unlist(pkimodelsmean)

pki_ps_raw = unlist(pkimodelspvals)
plot(log(pki_effect, base = 2), -log(pki_ps_raw, base = 10))
```

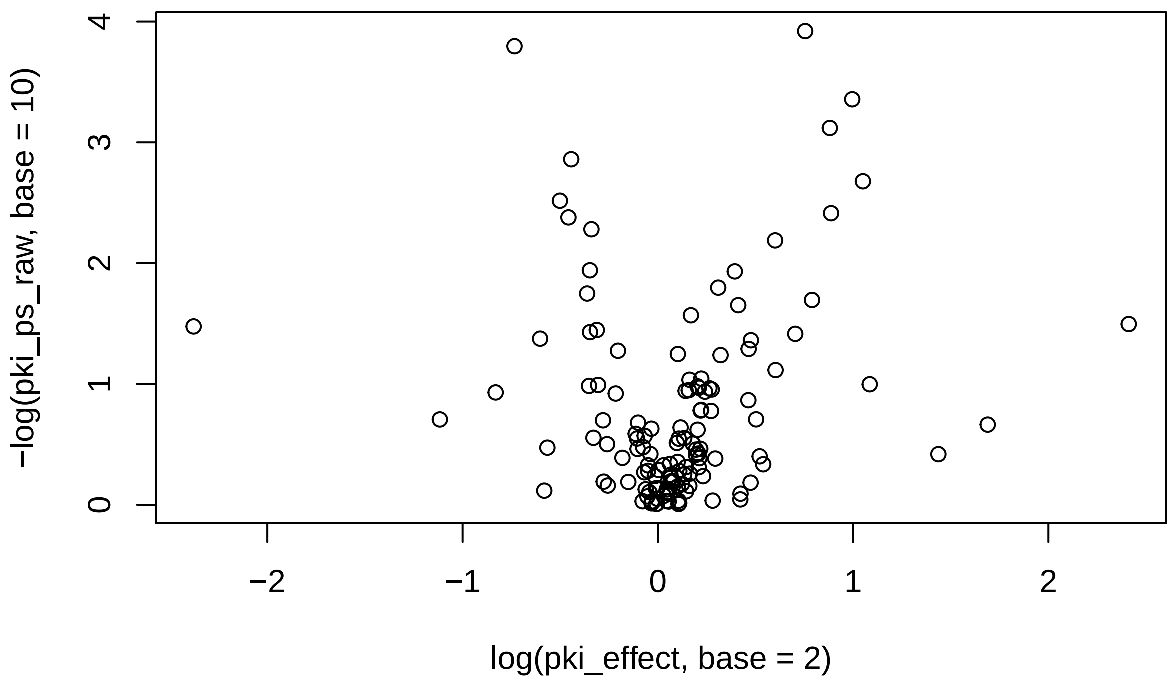


```r
pki_ps_adj <- p.adjust(pki_ps_raw, method = "BH")
plot(log(pki_effect, base = 2), -log(pki_ps_adj, base = 10))
```

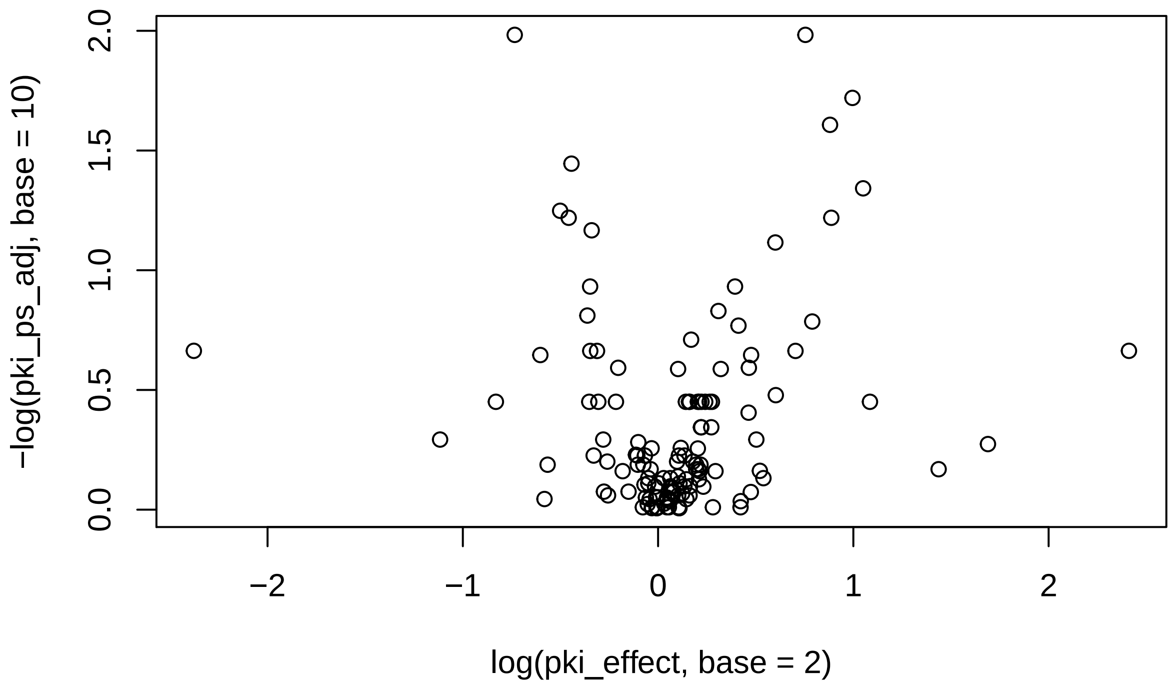


```
univariate_res_control = data.frame(variables = vars, pvalues = pki_ps_raw,
                                    adj_pvalues = pki_ps_adj,
                                    FoldChange = FoldChange,
                                    pkimodelsmean = pkimodelsmean)
univariate_res_control$significance_raw <- univariate_res_control$pvalues< .05
univariate_res_control$significance_adj <- univariate_res_control$adj_pvalues < .05
write.csv(univariate_res_control, "healthstate_anova_wsig_control_training_plasma_nonpara.txt", row.name
```

```
sigs <- read.csv("healthstate_anova_wsig_control_training_serum_nonpara.txt")
sum(sigs$significance_raw)
```

```
## [1] 23
```

```
sigs_training <- sigs$variables[(sigs$pvalues < .05) == T]

sigs <- read.csv("healthstate_anova_wsig_control_training_plasma_nonpara.txt")
sum(sigs$significance_raw)
```

```
## [1] 25
```

```
sigs_training <- sigs$variables[(sigs$pvalues < .05) == T]

sigs <- read.csv("healthstate_anova_wsig_control_training_block.txt")
sum(sigs$significance_raw)
```

```
## [1] 29
```

```r
sigs_training <- sigs$variables[(sigs$pvalues < .05) == T]

boxplot(d_serum$xylose~d_serum$Health_State, main = "xylose")
stripchart(d_serum$xylose ~ d_serum$Health_State, vertical = TRUE,
           method = "jitter", add = TRUE, pch = 20, col = 'blue')
```

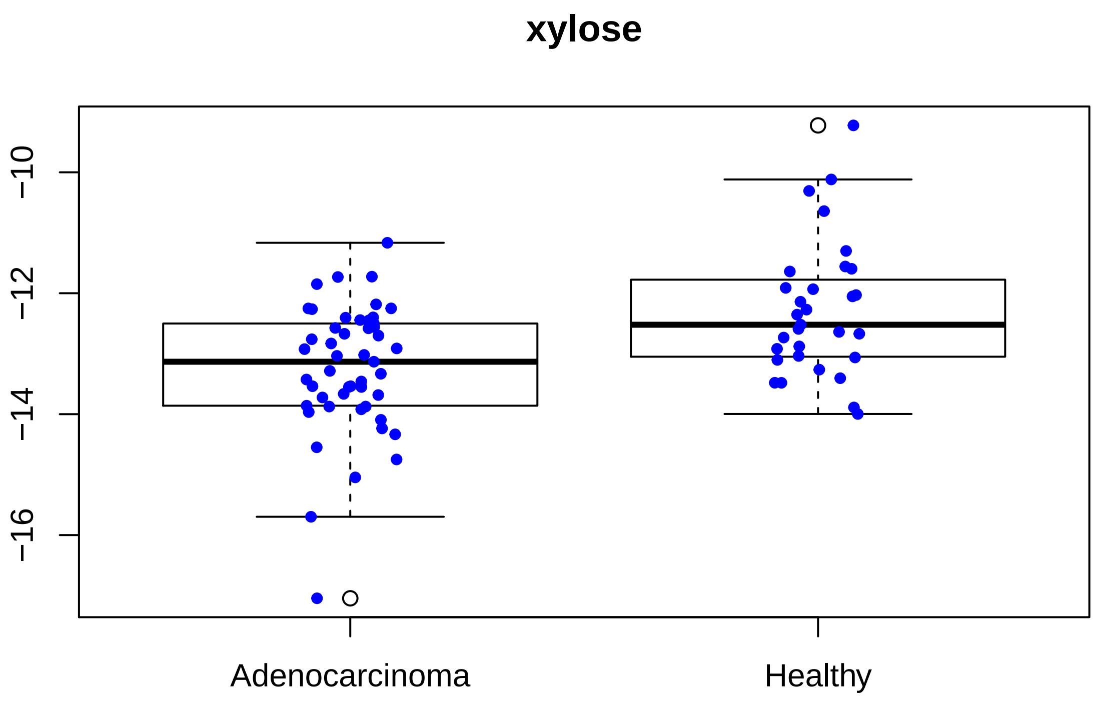


```r
boxplot(d_serum$glutamic_acid~d_serum$Health_State, main = "glutamate")
stripchart(d_serum$glutamic_acid~d_serum$Health_State, vertical = TRUE,
           method = "jitter", add = TRUE, pch = 20, col = 'blue')
```

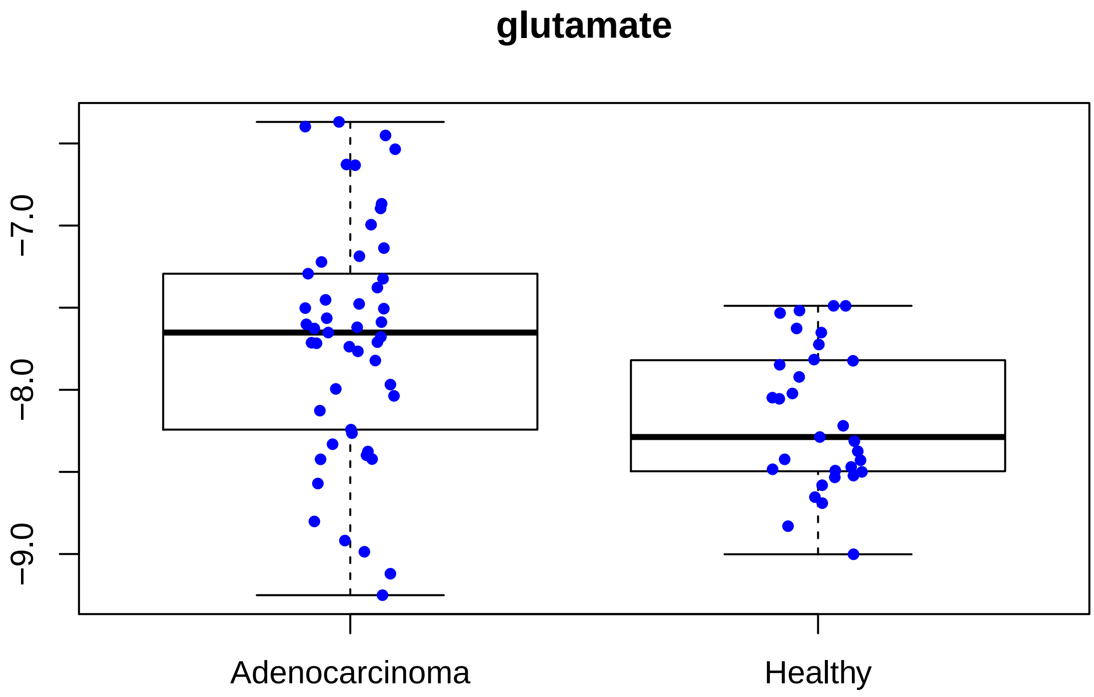


```r
boxplot(d_serum$aspartic_acid~d_serum$Health_State, main = "aspartate")
stripchart(d_serum$aspartic_acid~d_serum$Health_State, vertical = TRUE,
           method = "jitter", add = TRUE, pch = 20, col = 'blue')
```

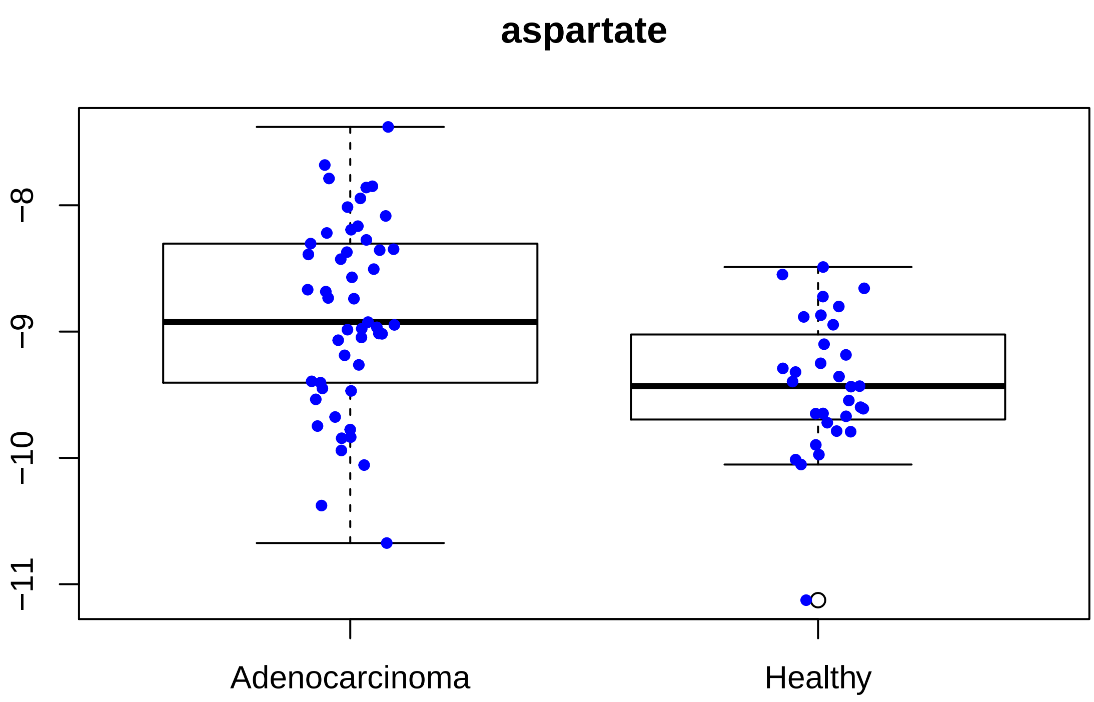

2 Test Set

```
rm(list = ls())
sdfset <- read.SDFset("common_test_training_molecule-v2.sdf")

# test for significance with only compounds that have chemical structures

f1 <- read.delim(file = "sample_factors_test.txt", header = T, row.names = 1)
f2 <- read.table(file = "sample_metabolites_test.txt", header = T, row.names = 1)
f1 <- na.omit(f1)
rownames(f1) <- f1$Sample_name
f1$Subject_name <- gsub("P_|S_", "", f1$Subject_name, perl = T)

f1 <- f1[, -2]
colnames(f1) <- c("Patient","Organ", "Health_State", "Smoking_Status", "Gender")
head(f1)
```

```
##                  Patient  Organ   Health_State Smoking_Status Gender
## 140225dlvsa44_1      20 Plasma Adenocarcinoma        Current      F
## 140226dlvsa30_1      36 Plasma Adenocarcinoma        Current      F
## 140226dlvsa36_1      39 Plasma Adenocarcinoma        Current      F
## 140227dlvsa36_1      61 Plasma Adenocarcinoma        Current      F
## 140227dlvsa47_1      66 Plasma Adenocarcinoma        Current      F
## 140228dlvsa08_1      71 Plasma Adenocarcinoma        Current      F
```

```r
f2 <- t(f2)

# save this processed data frame so you can try different processing
before.process <- f2

# two metabolites with missing values, use imputation, take half the minimum
# of that metabolite's value
# also, perform log base 2 transformation.  I cannot for the life of me
# figure out how they did their normalization
# lactic acid had some zero values? impute those too
f2 <- apply(f2, 2, function(x) {
  x[is.na(x)] <- .5*min(na.omit(x))
  x[x == 0] <- .5*min(na.omit(x[x != 0]))
  x
})

# # not necessary to do total quantity normalization
# # pvalues dont change see below
for(i in 1:nrow(f2)){
  f2[i, ] <- f2[i, ]/sum(f2[i, ])
}

# log transformation
f2 <- log(f2, base = 2)

summary(f2[, 1:6])
```

```
##  1_5-anhydroglucitol  1-monoolein     1-monopalmitin   1-monostearin
##  Min.   :-12.073     Min.   :-14.72   Min.   :-17.35   Min.   :-16.12
##  1st Qu.: -8.378     1st Qu.:-13.19   1st Qu.:-14.91   1st Qu.:-14.71
##  Median : -7.885     Median :-12.48   Median :-14.45   Median :-14.24
##  Mean   : -7.871     Mean   :-12.28   Mean   :-14.38   Mean   :-14.25
##  3rd Qu.: -7.166     3rd Qu.:-11.62   3rd Qu.:-13.83   3rd Qu.:-13.81
##  Max.   : -5.653     Max.   : -9.22   Max.   :-12.61   Max.   :-11.74
##  2_3_5-trihydroxypyrazine 2_3-dihydroxybutanoic_acid
##  Min.   :-18.61           Min.   :-16.84
##  1st Qu.:-15.77           1st Qu.:-15.02
##  Median :-15.41           Median :-14.55
##  Mean   :-15.47           Mean   :-14.48
##  3rd Qu.:-15.07           3rd Qu.:-13.99
##  Max.   :-14.18           Max.   :-11.24
```

```r
# fix compound names so that they are the same
# format as the file provided by Melaine
ids <- sdfid(sdfset)
ids.new <- gsub(" |,","_",ids, perl = T)
colnames(f2) <- gsub(" |,","_", colnames(f2), perl = T)

# 130 compounds provided
sum(ids.new %in% colnames(f2))
```

```
## [1] 130
```

```r
dim(f2)
```

```
## [1] 192 152
```

```r
f2 <- f2[, colnames(f2) %in% ids.new]
orig.mets <- colnames(f2)[colnames(f2) %in% ids.new]

rownames(f2) <- sub("X", "", row.names(f2))
d <- merge(f1, f2, by.x= "row.names", by.y = "row.names")

d$Organ <- factor(d$Organ)

#replacing mispelled adenocarcinoma and "Adenosquamous" with Adenocarcinoma
d$Health_State <- gsub("Adenocarcnoma|Adenosquamous", "Adenocarcinoma", d$Health_State)
row.names(d) <- d$Row.names
d$Row.names <- NULL
save.image("test_set_tq_nonormalize.rda")
```

2.1 Patient characteristic table

```r
table(d$Organ, d$Health_State)
```

```
##
##          Adenocarcinoma Healthy
##   Plasma             43      43
##   Serum              43      43
```

```r
table(d$Organ, d$Smoking_Status)
```

```
##
##          Current Former
##   Plasma      31     55
##   Serum       31     55
```

```r
table(d$Organ, d$Smoking_Status, d$Health_State)
```

```
## , ,  = Adenocarcinoma
##
##
##          Current Former
##   Plasma      15     28
##   Serum       15     28
##
## , ,  = Healthy
##
##
##          Current Former
##   Plasma      16     27
##   Serum       16     27
```

```r
table(d$Organ, d$Gender)
```

```
##
##           F  M
##   Plasma 46 40
```

```
##   Serum  46 40
```

```
table(d$Organ, d$Gender, d$Health_State)
```

```
## , ,  = Adenocarcinoma
##
##
##           F  M
##   Plasma 24 19
##   Serum  24 19
##
## , ,  = Healthy
##
##
##           F  M
##   Plasma 22 21
##   Serum  22 21
```

2.2 T-Tests for Significant differences

```
vars = colnames(d)[6:ncol(d)]
varNum <- length(vars)
pkimodels <- vector("list", (varNum))
pkimodelspvals <- vector("list", (varNum))
pkimodelseffect <- vector("list", (varNum))
pkimodelsmean <- vector("list", (varNum))

#----controling for all factors, including organ

for (i in 1:(varNum)){
  lmfit <- lm(d[,i+5]~ Organ + Health_State + Smoking_Status + Gender, data = d)
  pkimodels[[i]] <- lmfit
  #pvalue for regressing each variable in df on AC50
  pkimodelspvals[[i]] <- Anova(lmfit, type = "III")$`Pr(>F)`[3]

  pkimodelseffect[[i]] <- log(mean(2^(d[d$Health_State == "Adenocarcinoma",i+5]))/mean(2^(d[d$Health_Sta
  # mins[[i]] <- min(d[,220])
}

pki_ps = unlist(pkimodelspvals)
pki_ps <- p.adjust(pki_ps, method = "BH")

pki_effect = unlist(pkimodelseffect)
plot(pki_effect, -log(pki_ps, base = 10))
```

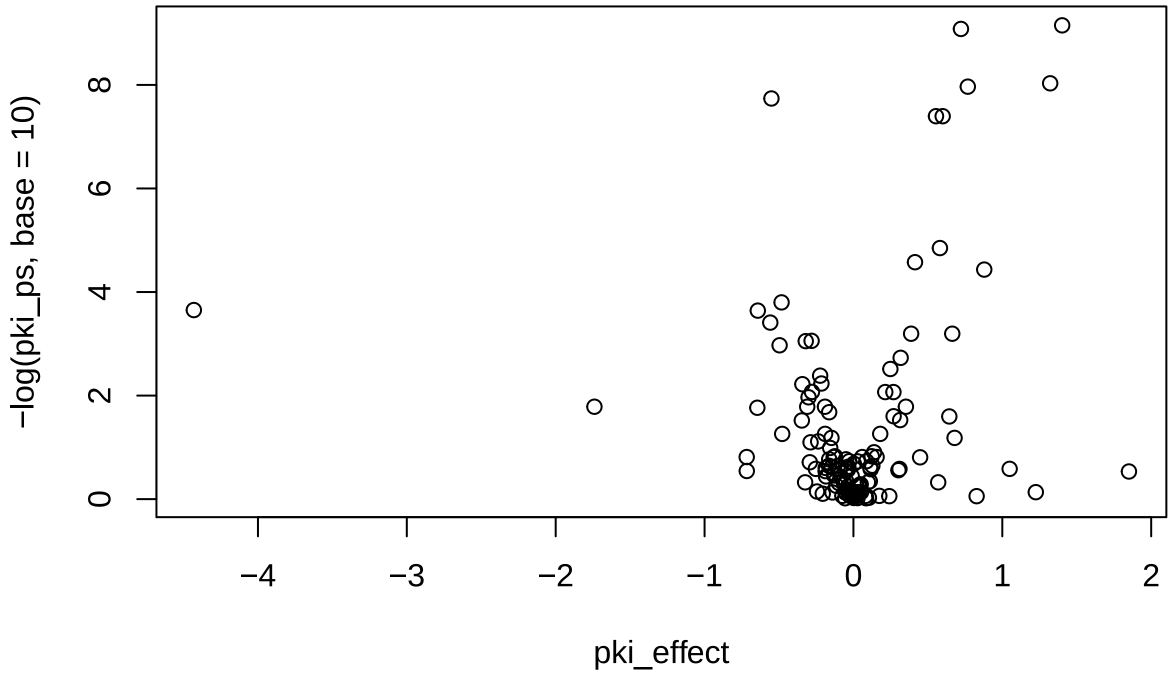


```
log2FoldChange <- pki_effect

univariate_res_control = data.frame(variables = vars, pvalues = pki_ps, log2FoldChange = log2FoldChange)
univariate_res_control$significance <- univariate_res_control$pvalues < .05
sig_no_block <- univariate_res_control$pvalues < .05
write.csv(univariate_res_control, "healthstate_anova_wsig_control_test.txt", row.names = F)

#-----Using patient as blocking factor

for (i in 1:(varNum)){
  lmfit <- lmer(d[,i+5]~ Organ + Health_State + Smoking_Status + Gender + (1|Patient), data = d)
  pkimodels[[i]] <- lmfit
  #pvalue for regressing each variable in df on AC50
  pkimodelspvals[[i]] <- Anova(lmfit, type = "III")$`Pr(>Chisq)`[3]

  pkimodelseffect[[i]] <- mean(2^(d[d$Health_State == "Adenocarcinoma",i+5]))/mean(2^(d[d$Health_State =
  # mins[[i]] <- min(d[,220])
}

pki_effect = unlist(pkimodelseffect)
log2FoldChange <- pki_effect

pki_ps_raw = unlist(pkimodelspvals)
plot(log(pki_effect, base = 2), -log(pki_ps_raw, base = 10))
```

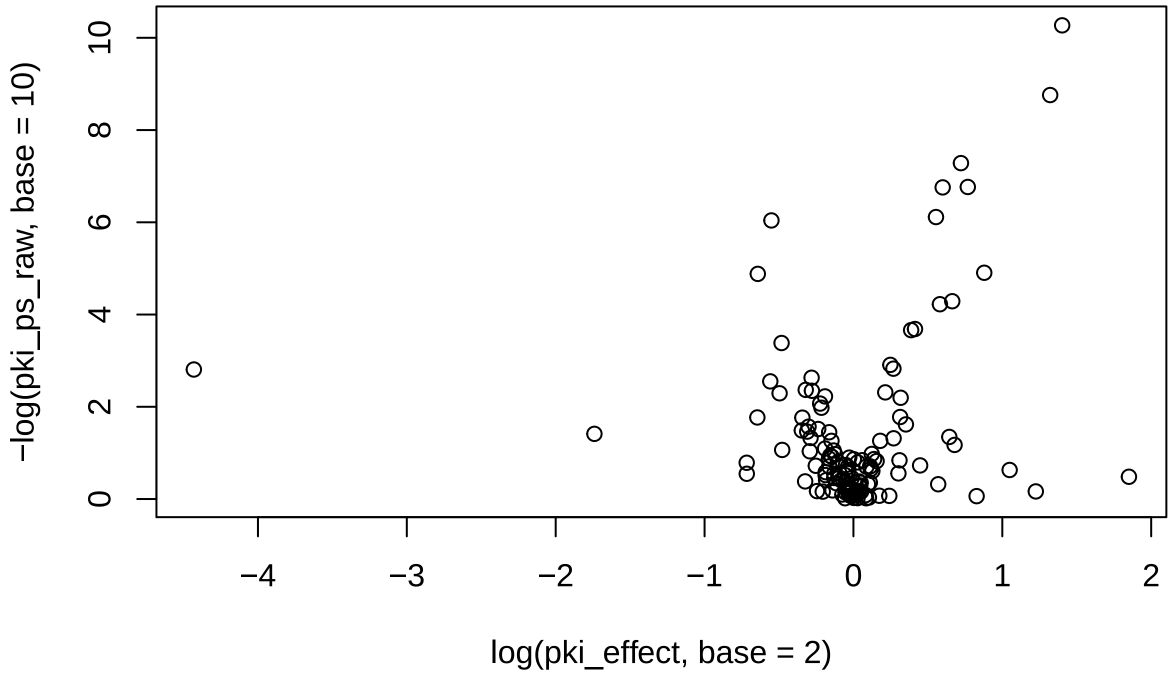


```
pki_ps_adj <- p.adjust(pki_ps_raw, method = "BH")
plot(log(pki_effect, base = 2), -log(pki_ps_adj, base = 10))
```

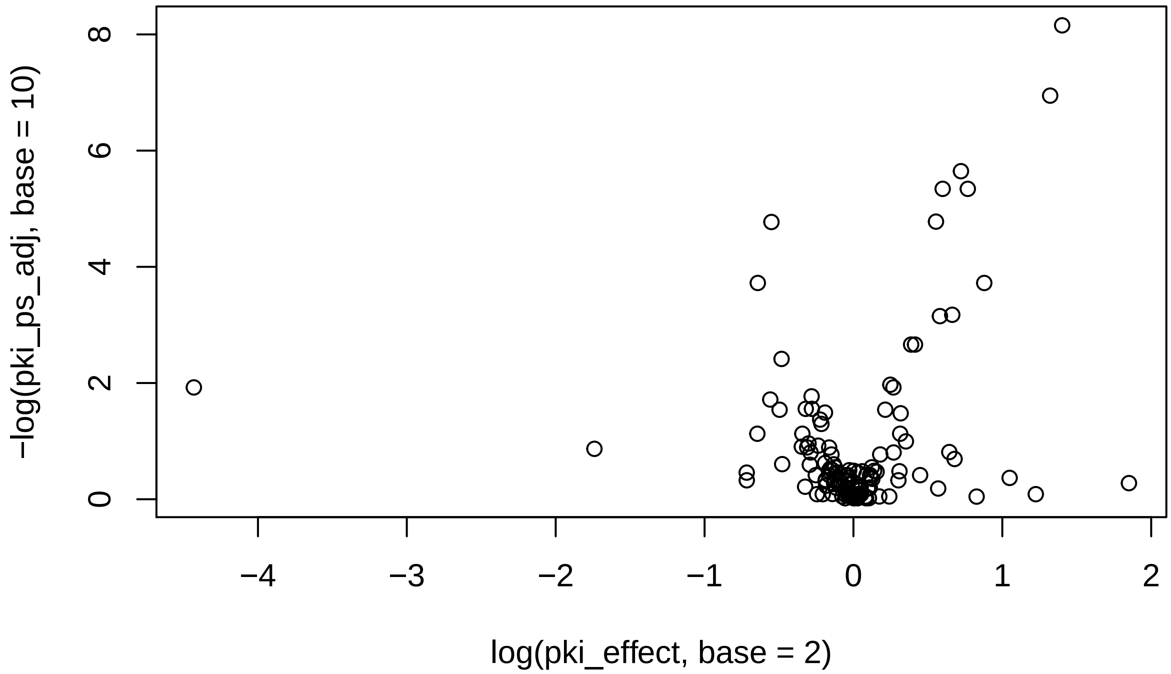


```r
univariate_res_control = data.frame(variables = vars, pvalues = pki_ps_raw,
                                    adj_pvalues = pki_ps_adj,
                                    FoldChange = log2FoldChange)
univariate_res_control$significance_raw <- univariate_res_control$pvalues < .05
univariate_res_control$significance_adj <- univariate_res_control$adj_pvalues < .05
write.csv(univariate_res_control, "healthstate_anova_wsig_control_test_block.txt", row.names = F)


#----only analyze the samples collected from Serum
d_serum <- d[d$Organ == "Serum", ]
#----controling for all factors
for (i in 1:(varNum)){
  lmfit <- lm(d_serum[,i+5]~ Health_State + Smoking_Status + Gender, data = d_serum)
  pkimodels[[i]] <- lmfit
  #pvalue for regressing each variable in df on AC50
  pkimodelspvals[[i]] <- Anova(lmfit, type = "III")$`Pr(>F)`[3]

  pkimodelseffect[[i]] <-
    log(mean(2^(d_serum[d_serum$Health_State == "Adenocarcinoma",i+5]))/mean(2^(d_serum[d_serum$Health_
  pkimodelsmean[[i]] <- mean(d_serum[d_serum$Health_State == "Adenocarcinoma",i+5])
}

pki_ps = unlist(pkimodelspvals)
pki_ps <- p.adjust(pki_ps, method = "BH")

pki_effect = unlist(pkimodelseffect)
```

```r
plot(pki_effect, -log(pki_ps, base = 10))
```

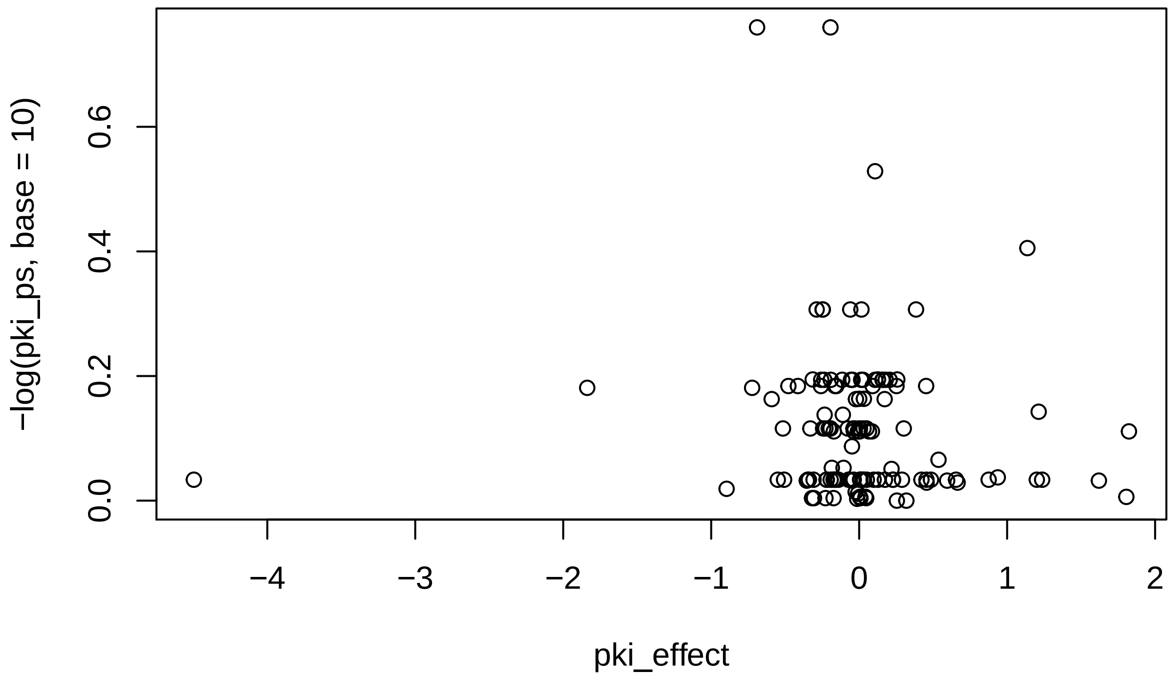


```r
log2FoldChange <- pki_effect
pkimodelsmean <- unlist(pkimodelsmean)

univariate_res_control = data.frame(variables = vars, pvalues = pki_ps,
                                    log2FoldChange = log2FoldChange,
                                    pkimodelsmean = pkimodelsmean)
univariate_res_control$significance <- univariate_res_control$pvalues < .05

# # use FDR of .2 instead
# pki_ps[which(univariate_res_control$pvalues < .2)]
# univariate_res_control$significance <- univariate_res_control$pvalues < .2
sum(univariate_res_control$significance)
```

```
## [1] 0
```

```r
write.csv(univariate_res_control, "healthstate_anova_wsig_control_test_serum.txt", row.names = F)

#----only analyze the samples collected from plasma
d_plasma <- d[d$Organ == "Plasma", ]

#----controling for all factors
for (i in 1:(varNum)){
  lmfit <- lm(d_plasma[,i+5]~ Health_State + Smoking_Status + Gender, data = d_plasma)
  pkimodels[[i]] <- lmfit
  #pvalue for regressing each variable in df on AC50
```

```
  pkimodelspvals[[i]] <- Anova(lmfit, type = "III")$`Pr(>F)`[3]

  pkimodelseffect[[i]] <-
    log(mean(2^(d_plasma[d_plasma$Health_State == "Adenocarcinoma",i+5]))/
          mean(2^(d_plasma[d_plasma$Health_State == "Healthy",i+5])), base=2)
  pkimodelsmean[[i]] <- mean(d_plasma[d_plasma$Health_State == "Adenocarcinoma",i+5])

}

pki_ps = unlist(pkimodelspvals)
pki_ps <- p.adjust(pki_ps, method = "BH")

pki_effect = unlist(pkimodelseffect)
plot(pki_effect, -log(pki_ps, base = 10))
```

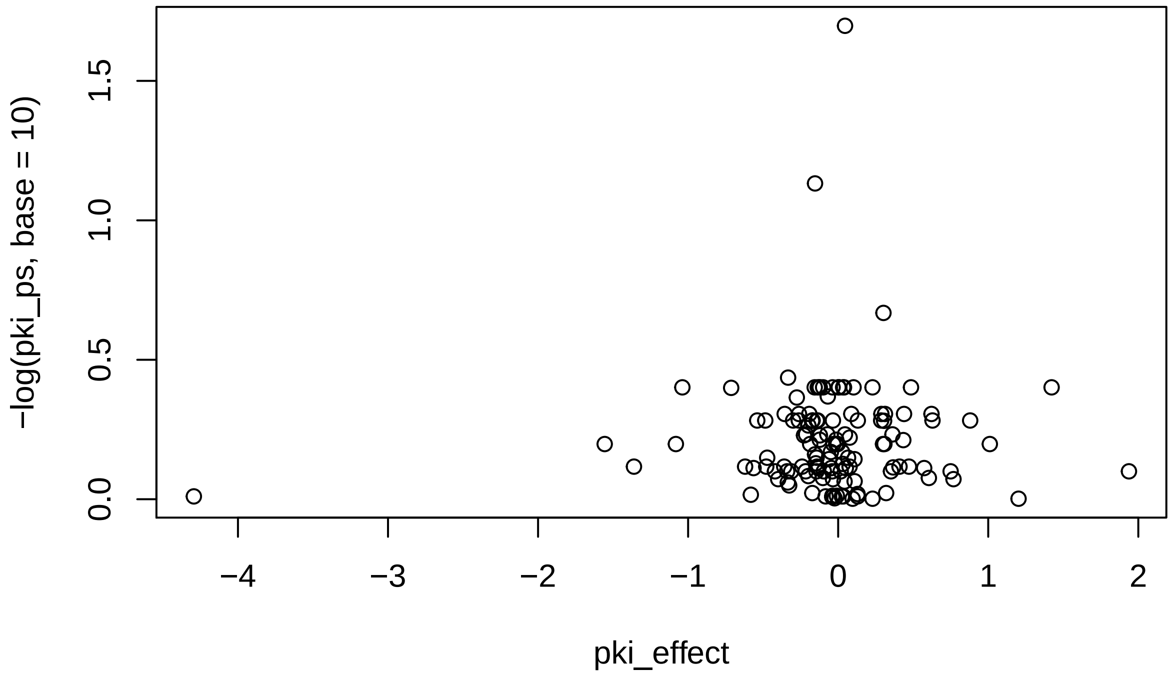


```
log2FoldChange <- pki_effect
pkimodelsmean <- unlist(pkimodelsmean)

univariate_res_control = data.frame(variables = vars, pvalues = pki_ps,
                                    log2FoldChange = log2FoldChange,
                                    pkimodelsmean = pkimodelsmean)
univariate_res_control$significance <- univariate_res_control$pvalues < .05
sum(univariate_res_control$significance)
```

```
## [1] 1
```

```r
write.csv(univariate_res_control, "healthstate_anova_wsig_control_test_plasma.txt", row.names = F)
```

2.3 Non-parameteric approach

```r
#----only analyze the samples collected from Serum
d_serum <- d[d$Organ == "Serum", ]
#----controling for all factors
for (i in 1:(varNum)){
  lmfit <- lm(d_serum[,i+5]~ Smoking_Status + Gender, data = d_serum)
  pkimodelspvals[[i]] <-   permTS(lmfit$residuals ~ Health_State, data = d_serum,
                                  alternative="two.sided", method="exact.mc",
                                  control=permControl(nmc=10^5))$p.value
  # Switched to reporting FC instead of logFC here so that can get right input for volcano plot
  # on metabolomics workbench
  pkimodelseffect[[i]] <-
    mean(2^(d_serum[d_serum$Health_State == "Adenocarcinoma",i+5]))/mean(2^(d_serum[d_serum$Health_Stat
  pkimodelsmean[[i]] <- mean(d_plasma[d_plasma$Health_State == "Adenocarcinoma",i+5])
}

pki_effect = unlist(pkimodelseffect)
FoldChange <- pki_effect
pkimodelsmean <- unlist(pkimodelsmean)

pki_ps_raw = unlist(pkimodelspvals)
plot(log(pki_effect, base = 2), -log(pki_ps_raw, base = 10))
```

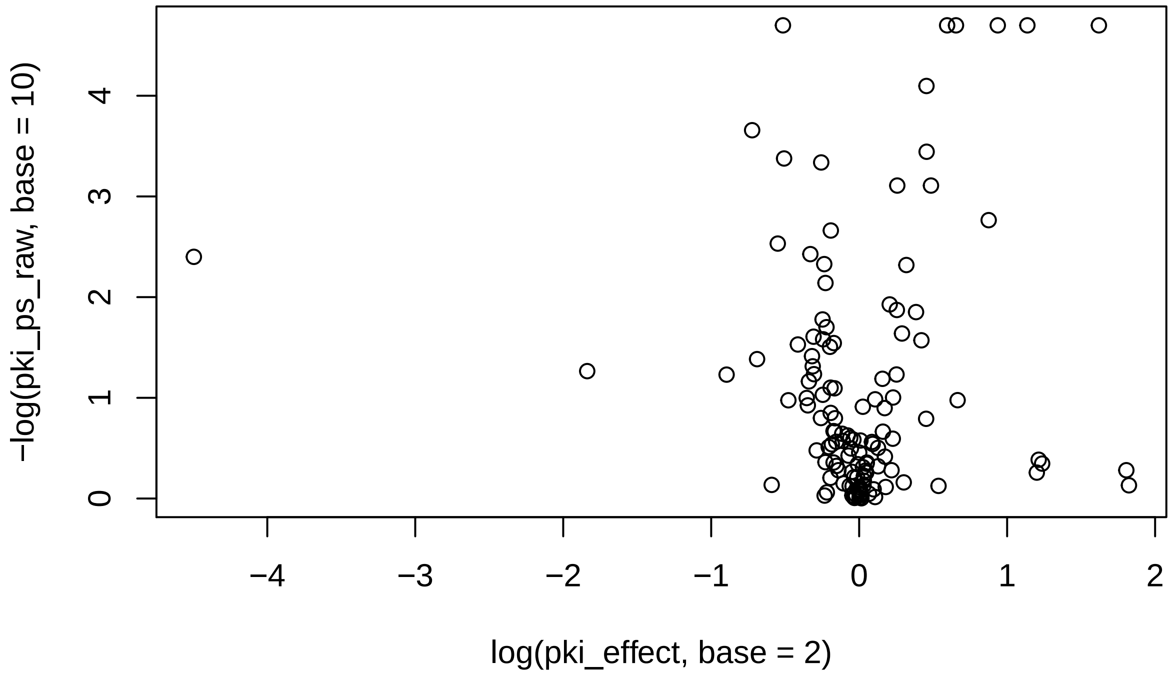


```
pki_ps_adj <- p.adjust(pki_ps_raw, method = "BH")
plot(log(pki_effect, base = 2), -log(pki_ps_adj, base = 10))
```

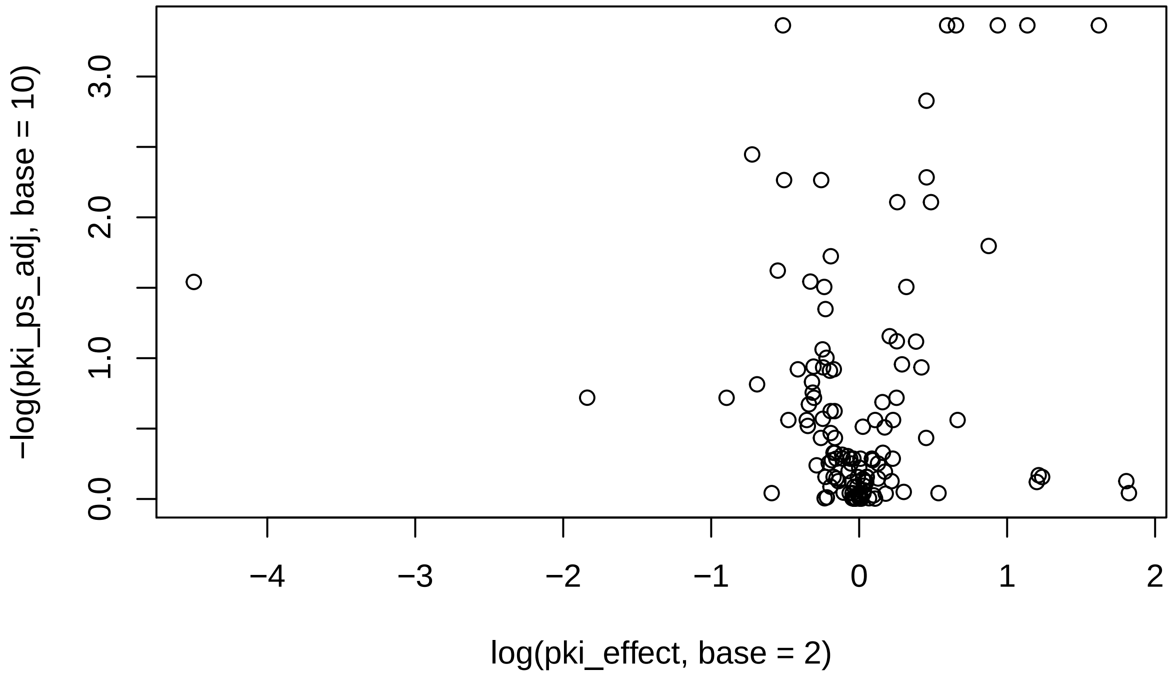


```r
univariate_res_control = data.frame(variables = vars, pvalues = pki_ps_raw,
                                    adj_pvalues = pki_ps_adj,
                                    FoldChange = FoldChange,
                                    pkimodelsmean = pkimodelsmean)
univariate_res_control$significance_raw <- univariate_res_control$pvalues < .05
univariate_res_control$significance_adj <- univariate_res_control$adj_pvalues < .05
write.csv(univariate_res_control, "healthstate_anova_wsig_control_test_serum_nonpara.txt", row.names = F

#----only analyze the samples collected from Plasma
d_plasma <- d[d$Organ == "Plasma", ]

#----controling for all factors
for (i in 1:(varNum)){
  lmfit <- lm(d_plasma[,i+5]~ Smoking_Status + Gender, data = d_plasma)
  pkimodelspvals[[i]] <-   permTS(lmfit$residuals ~ Health_State, data = d_plasma,
                                  alternative="two.sided", method="exact.mc",
                                  control=permControl(nmc=10^5))$p.value

  pkimodelseffect[[i]] <-
    mean(2^(d_plasma[d_plasma$Health_State == "Adenocarcinoma",i+5]))/
          mean(2^(d_plasma[d_plasma$Health_State == "Healthy",i+5]))
  pkimodelsmean[[i]] <- mean(d_plasma[d_plasma$Health_State == "Adenocarcinoma",i+5])

}

pki_effect = unlist(pkimodelseffect)
```

```r
FoldChange <- pki_effect
pkimodelsmean <- unlist(pkimodelsmean)

pki_ps_raw = unlist(pkimodelspvals)
plot(log(pki_effect, base = 2), -log(pki_ps_raw, base = 10))
```

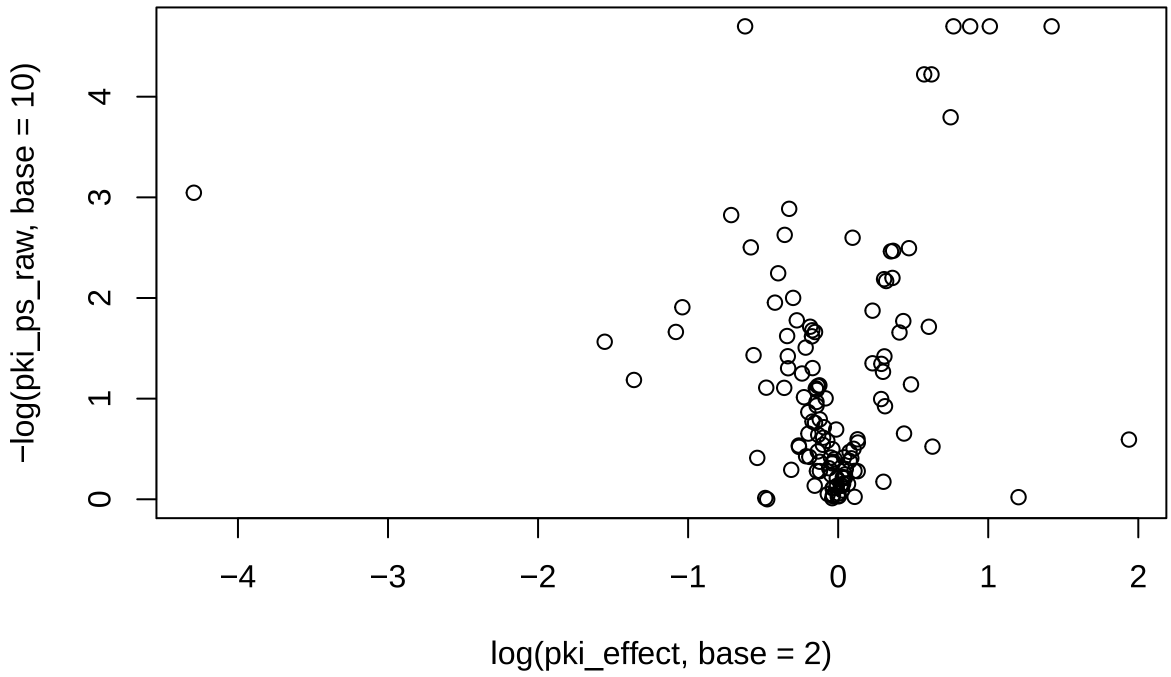


```r
pki_ps_adj <- p.adjust(pki_ps_raw, method = "BH")
plot(log(pki_effect, base = 2), -log(pki_ps_adj, base = 10))
```

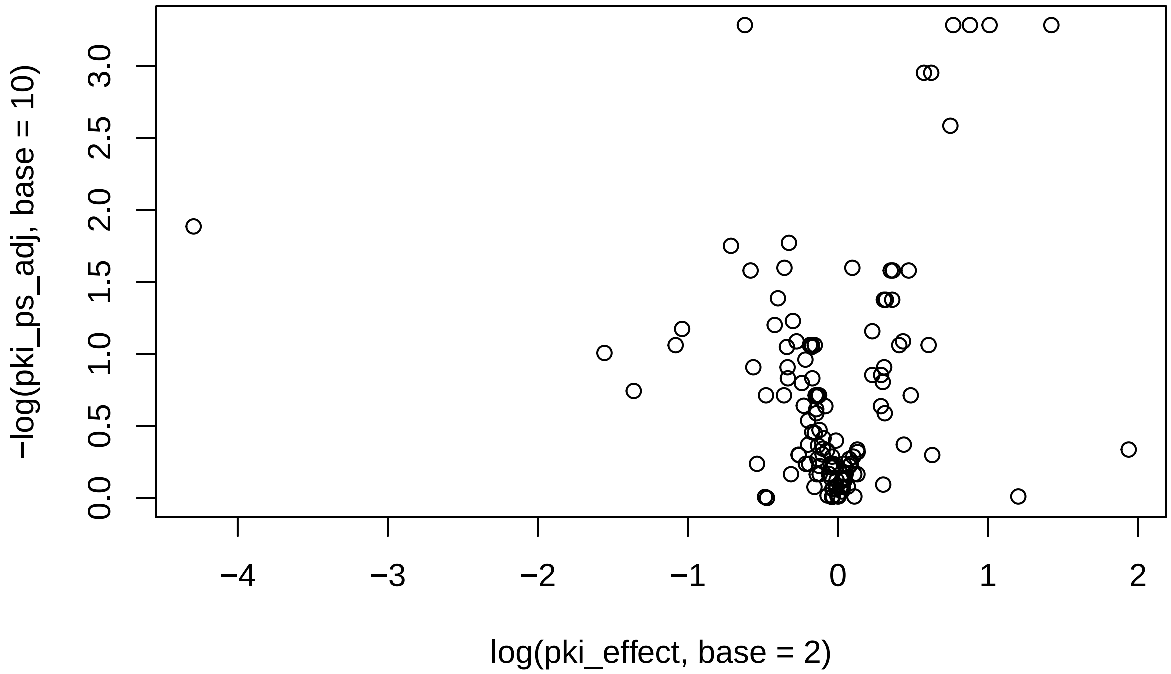


```
univariate_res_control = data.frame(variables = vars, pvalues = pki_ps_raw,
                                    adj_pvalues = pki_ps_adj,
                                    FoldChange = FoldChange,
                                    pkimodelsmean = pkimodelsmean)
univariate_res_control$significance_raw <- univariate_res_control$pvalues< .05
univariate_res_control$significance_adj <- univariate_res_control$adj_pvalues < .05
write.csv(univariate_res_control, "healthstate_anova_wsig_control_test_plasma_nonpara.txt", row.names =
```

```
sigs <- read.csv("healthstate_anova_wsig_control_test_serum_nonpara.txt")
sum(sigs$significance_raw)
```

```
## [1] 36
```

```
sigs_test <- sigs$variables[(sigs$pvalues < .05) == T]

sigs <- read.csv("healthstate_anova_wsig_control_test_plasma_nonpara.txt")
sum(sigs$significance_raw)
```

```
## [1] 44
```

```
sigs_test <- sigs$variables[(sigs$pvalues < .05) == T]

sigs <- read.csv("healthstate_anova_wsig_control_test_block.txt")
sum(sigs$significance_raw)
```

```
## [1] 40
```

```r
sigs_test <- sigs$variables[(sigs$pvalues < .05) == T]

boxplot(d_serum$xylose~d_serum$Health_State, main = "xylose")
stripchart(d_serum$xylose ~ d_serum$Health_State, vertical = TRUE,
           method = "jitter", add = TRUE, pch = 20, col = 'blue')
```

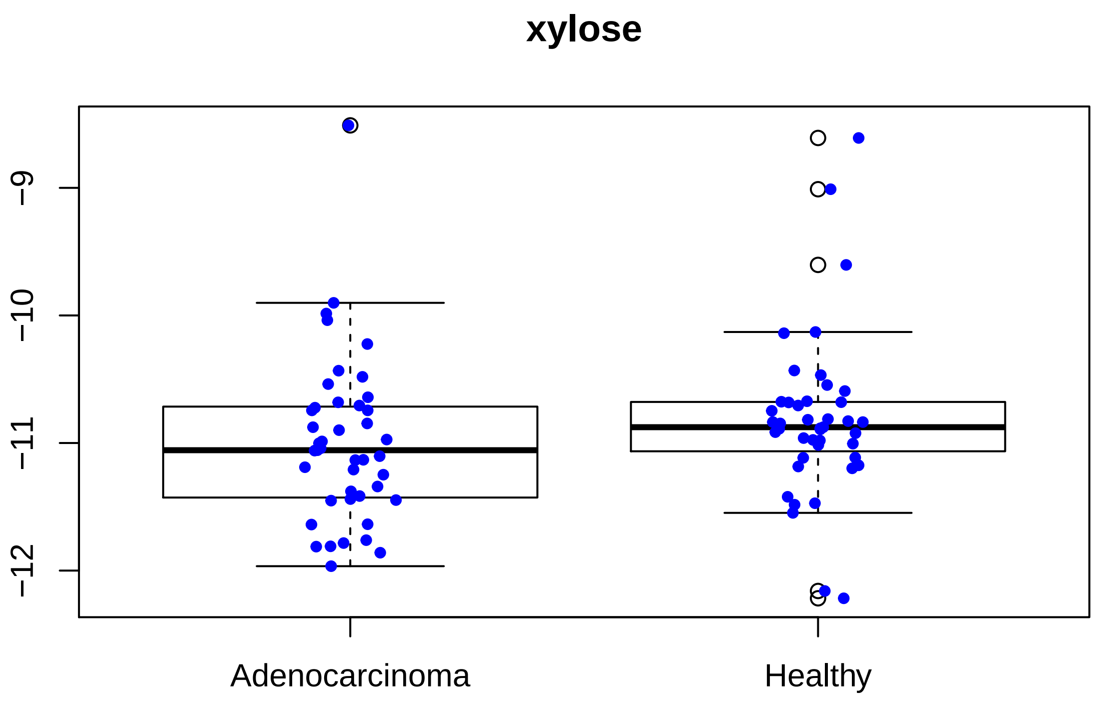


```r
boxplot(d_serum$glutamic_acid~d_serum$Health_State, main = "glutamate")
stripchart(d_serum$glutamic_acid~d_serum$Health_State, vertical = TRUE,
           method = "jitter", add = TRUE, pch = 20, col = 'blue')
```

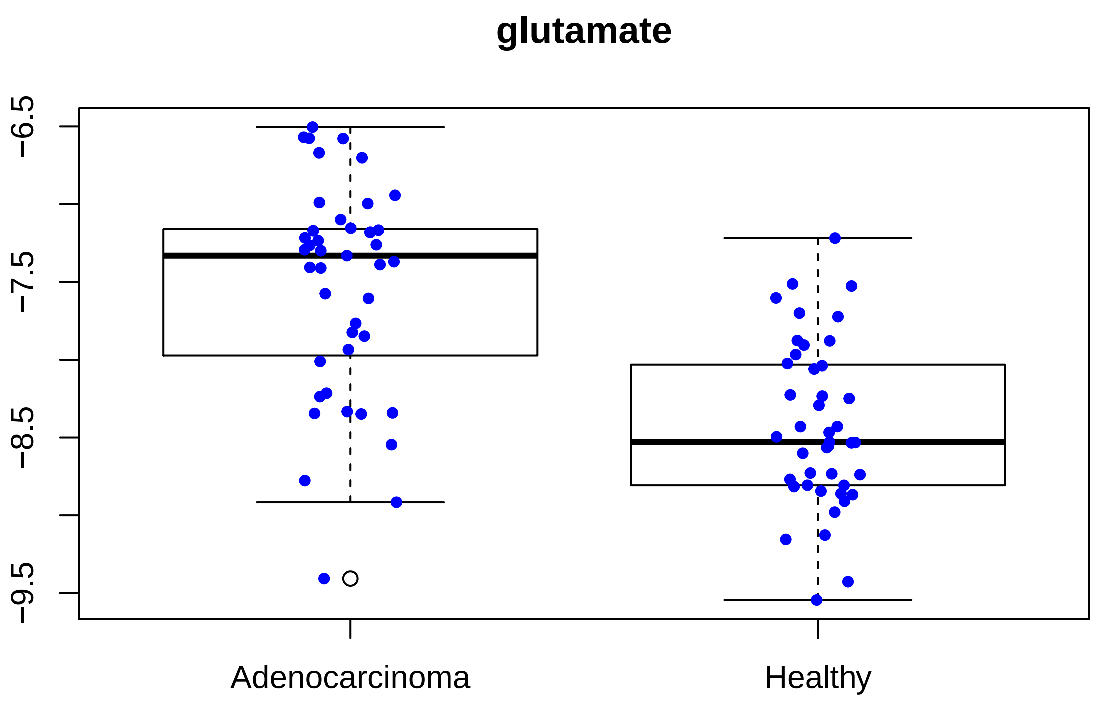


```r
boxplot(d_serum$aspartic_acid~d_serum$Health_State, main = "aspartate")
stripchart(d_serum$aspartic_acid~d_serum$Health_State, vertical = TRUE,
           method = "jitter", add = TRUE, pch = 20, col = 'blue')
```

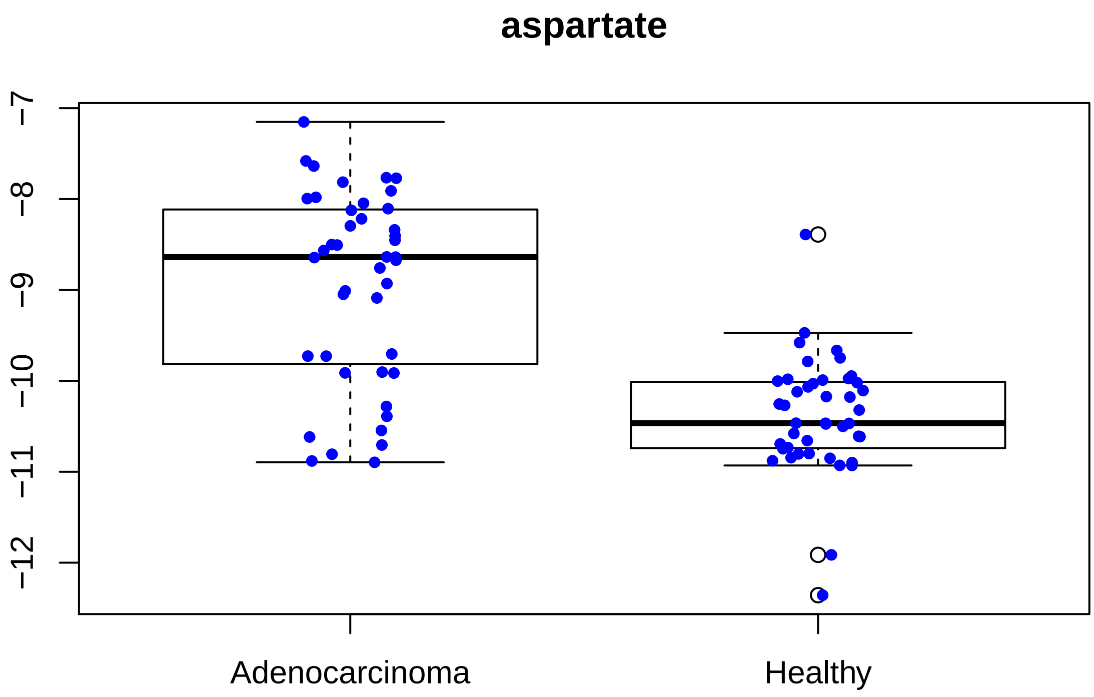
aspartate
−7
−8
−9
−10
−11
−12
Adenocarcinoma
Healthy
