# Supplementary material for: Cheminformatics approach to exploring and modeling trait-associated metabolite profiles
Source: J Cheminform. 2019 Jun 24;11:43. doi: 10.1186/s13321-019-0366-3 (PMC6591908; doi:10.1186/s13321-019-0366-3)
Supplement: Supplementary file 2 — Additional file 2. The scripts and additional data necessary to recreate our analyses. [file 13321_2019_366_MOESM2_ESM.zip › metabochem-master/analyses/metab_classifier_plasma.html]

Metabolomics ML results – Plasma


# Metabolomics ML results – Plasma

#### *Jeremy Ash*

#### *October 13, 2018*

## 0.1 All metabolites

```
load("ML_data.RDATA")
```

### 0.1.1 Lasso

```
all.acc <- matrix(nrow = 6, ncol = 8)
colnames(all.acc) <- c(rep("LOOCV", 4), rep("External", 4))
colnames(all.acc) <- paste(colnames(all.acc), rep(c("ACC", "SEN", "SPE", "AUC"), 2))
rownames(all.acc) <- c("Lasso", "Logit Boost", "SVM", "RF", "PLS", "xgbTree")

health_df_plasma_full <- health_df_plasma_full[, -c(2, 3)]
health_df_plasma_full.test <- health_df_plasma_full.test[, -c(2, 3)]

all.acc <- FitLasso(health_df_plasma_full, health_df_plasma_full.test, all.acc)
```

```
## Warning: Option grouped=FALSE enforced in cv.glmnet, since < 3 observations
## per fold
```

### 0.1.2 Other ML

```
data <- health_df_plasma_full
data$Health_State <- as.factor(ifelse(data$Health_State == 0, "neg", "pos"))

data.test <- health_df_plasma_full.test
data.test$Health_State <- as.factor(ifelse(data.test$Health_State == 0, "neg", "pos"))
```

```
all.acc <- FitMlmodels(data, data.test, all.acc)
```

```
all.acc %>%  kable() %>%  kable_styling()
```

|  | LOOCV ACC | LOOCV SEN | LOOCV SPE | LOOCV AUC | External ACC | External SEN | External SPE | External AUC |
| --- | --- | --- | --- | --- | --- | --- | --- | --- |
| Lasso | 0.7195122 | 0.5806452 | 0.8039216 | 0.7545857 | 0.5348837 | 0.8837209 | 0.1860465 | 0.6565711 |
| Logit Boost | 0.6829268 | 0.8039216 | 0.4838710 | 0.6729918 | 0.6279070 | 0.6976744 | 0.5581395 | 0.6765819 |
| SVM | 0.7926829 | 0.9215686 | 0.5806452 | 0.8013915 | 0.6976744 | 0.6976744 | 0.6976744 | 0.7209302 |
| RF | 0.6951220 | 0.8823529 | 0.3870968 | 0.7118912 | 0.5000000 | 0.9534884 | 0.0465116 | 0.7890752 |
| PLS | 0.7682927 | 0.9215686 | 0.5161290 | 0.7685009 | 0.5930233 | 0.3023256 | 0.8837209 | 0.7279611 |
| xgbTree | 0.7682927 | 0.8235294 | 0.6774194 | 0.7596458 | 0.5813953 | 0.7906977 | 0.3720930 | 0.6522445 |

```
write.csv(all.acc, file = "plasma_all_met_acc.csv")
```

## 0.2 Significant metabolites

### 0.2.1 Lasso

```
all.acc <- matrix(nrow = 6, ncol = 8)
colnames(all.acc) <- c(rep("LOOCV", 4), rep("External", 4))
colnames(all.acc) <- paste(colnames(all.acc), rep(c("ACC", "SEN", "SPE", "AUC"), 2))
rownames(all.acc) <- c("Lasso", "Logit Boost", "SVM", "RF", "PLS", "xgbTree")

health_df_plasma_sig <- health_df_plasma_full[, c(1, plasma.met.idx - 3)]
health_df_plasma_sig.test <- health_df_plasma_full.test[, c(1, plasma.met.idx - 3)]

all.acc <- FitLasso(health_df_plasma_sig, health_df_plasma_sig.test, all.acc)
```

```
## Warning: Option grouped=FALSE enforced in cv.glmnet, since < 3 observations
## per fold
```

### 0.2.2 Other ML

```
data <- health_df_plasma_sig
data$Health_State <- as.factor(ifelse(data$Health_State == 0, "neg", "pos"))

data.test <- health_df_plasma_sig.test
data.test$Health_State <- as.factor(ifelse(data.test$Health_State == 0, "neg", "pos"))
```

```
all.acc <- FitMlmodels(data, data.test, all.acc)
```

```
## maximum number of iterations reached 2.96534e-05 -2.956871e-05maximum number of iterations reached 0.001331744 -0.001316847note: only 9 unique complexity parameters in default grid. Truncating the grid to 9 .
```

```
all.acc %>%  kable() %>%  kable_styling()
```

|  | LOOCV ACC | LOOCV SEN | LOOCV SPE | LOOCV AUC | External ACC | External SEN | External SPE | External AUC |
| --- | --- | --- | --- | --- | --- | --- | --- | --- |
| Lasso | 0.6829268 | 0.4516129 | 0.8235294 | 0.7602783 | 0.5813953 | 0.7906977 | 0.3720930 | 0.6944294 |
| Logit Boost | 0.7073171 | 0.8823529 | 0.4193548 | 0.6736243 | 0.6279070 | 0.8139535 | 0.4418605 | 0.6430503 |
| SVM | 0.7804878 | 0.7450980 | 0.8387097 | 0.7906388 | 0.6976744 | 0.4418605 | 0.9534884 | 0.7631152 |
| RF | 0.7317073 | 0.7843137 | 0.6451613 | 0.7356104 | 0.6627907 | 0.6976744 | 0.6279070 | 0.7376961 |
| PLS | 0.7682927 | 0.7450980 | 0.8064516 | 0.7963314 | 0.7093023 | 0.4651163 | 0.9534884 | 0.7917793 |
| xgbTree | 0.7682927 | 0.8823529 | 0.5806452 | 0.7305503 | 0.5465116 | 0.7674419 | 0.3255814 | 0.5540833 |

```
write.csv(all.acc, file = "plasma_sig_met_acc.csv")
```

## 0.3 Cluster 1 metabolites

### 0.3.1 Lasso

```
all.acc <- matrix(nrow = 6, ncol = 8)
colnames(all.acc) <- c(rep("LOOCV", 4), rep("External", 4))
colnames(all.acc) <- paste(colnames(all.acc), rep(c("ACC", "SEN", "SPE", "AUC"), 2))
rownames(all.acc) <- c("Lasso", "Logit Boost", "SVM", "RF", "PLS", "xgbTree")

health_df_plasma_clus1 <- health_df_plasma_sig[, c(1, 2, 10)]
health_df_plasma_clus1.test <- health_df_plasma_sig.test[, c(1, 2, 10)]
head(health_df_plasma_clus1)
```

```
##                 Health_State 3-phosphoglycerate pyrophosphate
## 130729dlvsa03_1            0          -14.03925    -11.322425
## 130729dlvsa05_1            0          -12.34514     -9.550447
## 130729dlvsa07_1            1          -13.71981     -9.478527
## 130729dlvsa09_1            1          -12.75529     -9.863338
## 130729dlvsa11_1            1          -12.92776     -9.091388
## 130729dlvsa13_1            1          -13.47954    -11.249555
```

```
all.acc <- FitLasso(health_df_plasma_clus1, health_df_plasma_clus1.test, all.acc)
```

```
## Warning: Option grouped=FALSE enforced in cv.glmnet, since < 3 observations
## per fold
```

### 0.3.2 Other ML

```
data <- health_df_plasma_clus1
data$Health_State <- as.factor(ifelse(data$Health_State == 0, "neg", "pos"))

data.test <- health_df_plasma_clus1.test
data.test$Health_State <- as.factor(ifelse(data.test$Health_State == 0, "neg", "pos"))
```

```
all.acc <- FitMlmodels(data, data.test, all.acc)
```

```
## note: only 1 unique complexity parameters in default grid. Truncating the grid to 1 .
```

```
all.acc %>%  kable() %>%  kable_styling()
```

|  | LOOCV ACC | LOOCV SEN | LOOCV SPE | LOOCV AUC | External ACC | External SEN | External SPE | External AUC |
| --- | --- | --- | --- | --- | --- | --- | --- | --- |
| Lasso | 0.7073171 | 0.5161290 | 0.8235294 | 0.7413030 | 0.5581395 | 0.7674419 | 0.3488372 | 0.6603569 |
| Logit Boost | 0.6951220 | 0.7843137 | 0.5483871 | 0.7188488 | 0.6162791 | 0.4418605 | 0.7906977 | 0.6879394 |
| SVM | 0.8048780 | 0.8627451 | 0.7096774 | 0.7134725 | 0.7093023 | 0.7209302 | 0.6976744 | 0.6749594 |
| RF | 0.7073171 | 0.8039216 | 0.5483871 | 0.7194813 | 0.6976744 | 0.6511628 | 0.7441860 | 0.7447269 |
| PLS | 0.7317073 | 0.9411765 | 0.3870968 | 0.7482606 | 0.6976744 | 0.6744186 | 0.7209302 | 0.7506760 |
| xgbTree | 0.7926829 | 0.8627451 | 0.6774194 | 0.7520557 | 0.7093023 | 0.6511628 | 0.7674419 | 0.7082207 |

```
write.csv(all.acc, file = "plasma_clus1_met_acc.csv")
```

## 0.4 Cluster 2 metabolites

### 0.4.1 Lasso

```
all.acc <- matrix(nrow = 6, ncol = 8)
colnames(all.acc) <- c(rep("LOOCV", 4), rep("External", 4))
colnames(all.acc) <- paste(colnames(all.acc), rep(c("ACC", "SEN", "SPE", "AUC"), 2))
rownames(all.acc) <- c("Lasso", "Logit Boost", "SVM", "RF", "PLS", "xgbTree")

health_df_plasma_clus2 <- health_df_plasma_sig[, c(1, 4, 5, 6, 7)]
health_df_plasma_clus2.test <- health_df_plasma_sig.test[, c(1, 4, 5, 6, 7)]
head(health_df_plasma_clus2)
```

```
##                 Health_State citrulline    cystine  histidine    lysine
## 130729dlvsa03_1            0  -11.15250  -8.058576  -7.113206 -5.231324
## 130729dlvsa05_1            0  -11.54347  -8.933416  -8.146579 -6.558496
## 130729dlvsa07_1            1  -12.31339  -8.703090  -8.213274 -6.022346
## 130729dlvsa09_1            1  -12.09048  -8.879627  -7.975002 -5.991853
## 130729dlvsa11_1            1  -11.80416 -11.657022  -9.265489 -6.462022
## 130729dlvsa13_1            1  -11.46748 -10.422579 -10.068437 -6.930933
```

```
all.acc <- FitLasso(health_df_plasma_clus2, health_df_plasma_clus2.test, all.acc)
```

```
## Warning: Option grouped=FALSE enforced in cv.glmnet, since < 3 observations
## per fold
```

### 0.4.2 Other ML

```
data <- health_df_plasma_clus2
data$Health_State <- as.factor(ifelse(data$Health_State == 0, "neg", "pos"))

data.test <- health_df_plasma_clus2.test
data.test$Health_State <- as.factor(ifelse(data.test$Health_State == 0, "neg", "pos"))
```

```
all.acc <- FitMlmodels(data, data.test, all.acc)
```

```
## maximum number of iterations reached 0.0007375663 -0.000731812maximum number of iterations reached 0.001685036 -0.001652211note: only 3 unique complexity parameters in default grid. Truncating the grid to 3 .
```

```
all.acc %>%  kable() %>%  kable_styling()
```

|  | LOOCV ACC | LOOCV SEN | LOOCV SPE | LOOCV AUC | External ACC | External SEN | External SPE | External AUC |
| --- | --- | --- | --- | --- | --- | --- | --- | --- |
| Lasso | 0.6829268 | 0.5161290 | 0.7843137 | 0.6963947 | 0.4883721 | 0.0000000 | 0.9767442 | 0.7133586 |
| Logit Boost | 0.6463415 | 0.9019608 | 0.2258065 | 0.6505376 | 0.5000000 | 0.9534884 | 0.0465116 | 0.4591671 |
| SVM | 0.7073171 | 0.7058824 | 0.7096774 | 0.6805819 | 0.4767442 | 0.9534884 | 0.0000000 | 0.7079502 |
| RF | 0.6463415 | 0.5686275 | 0.7741935 | 0.6166983 | 0.5465116 | 0.8604651 | 0.2325581 | 0.6427799 |
| PLS | 0.6951220 | 0.7843137 | 0.5483871 | 0.6837445 | 0.4767442 | 0.9302326 | 0.0232558 | 0.7133586 |
| xgbTree | 0.6951220 | 0.8627451 | 0.4193548 | 0.6344086 | 0.5232558 | 0.9534884 | 0.0930233 | 0.5381287 |

```
write.csv(all.acc, file = "plasma_clus2_met_acc.csv")
```

## 0.5 Cluster 3 metabolites

### 0.5.1 Lasso

```
all.acc <- matrix(nrow = 6, ncol = 8)
colnames(all.acc) <- c(rep("LOOCV", 4), rep("External", 4))
colnames(all.acc) <- paste(colnames(all.acc), rep(c("ACC", "SEN", "SPE", "AUC"), 2))
rownames(all.acc) <- c("Lasso", "Logit Boost", "SVM", "RF", "PLS", "xgbTree")

health_df_plasma_clus3 <- health_df_plasma_sig[, c(1, 8, 9)]
health_df_plasma_clus3.test <- health_df_plasma_sig.test[, c(1, 8, 9)]
head(health_df_plasma_clus3)
```

```
##                 Health_State   maltose maltotriose
## 130729dlvsa03_1            0 -14.15831   -14.67510
## 130729dlvsa05_1            0 -11.26158   -12.40367
## 130729dlvsa07_1            1 -11.61494   -12.76635
## 130729dlvsa09_1            1 -11.45940   -12.30120
## 130729dlvsa11_1            1 -10.23651   -11.22834
## 130729dlvsa13_1            1 -11.55660   -13.62189
```

```
all.acc <- FitLasso(health_df_plasma_clus3, health_df_plasma_clus3.test, all.acc)
```

```
## Warning: Option grouped=FALSE enforced in cv.glmnet, since < 3 observations
## per fold
```

### 0.5.2 Other ML

```
data <- health_df_plasma_clus3
data$Health_State <- as.factor(ifelse(data$Health_State == 0, "neg", "pos"))

data.test <- health_df_plasma_clus3.test
data.test$Health_State <- as.factor(ifelse(data.test$Health_State == 0, "neg", "pos"))
```

```
all.acc <- FitMlmodels(data, data.test, all.acc)
```

```
## note: only 1 unique complexity parameters in default grid. Truncating the grid to 1 .
```

```
all.acc %>%  kable() %>%  kable_styling()
```

|  | LOOCV ACC | LOOCV SEN | LOOCV SPE | LOOCV AUC | External ACC | External SEN | External SPE | External AUC |
| --- | --- | --- | --- | --- | --- | --- | --- | --- |
| Lasso | 0.7073171 | 0.4838710 | 0.8431373 | 0.6761543 | 0.5930233 | 0.9302326 | 0.2558140 | 0.7047052 |
| Logit Boost | 0.7073171 | 0.7450980 | 0.6451613 | 0.6723593 | 0.4883721 | 0.5116279 | 0.4651163 | 0.5146025 |
| SVM | 0.7804878 | 0.8823529 | 0.6129032 | 0.6356736 | 0.4302326 | 0.4883721 | 0.3720930 | 0.6311520 |
| RF | 0.7317073 | 0.9411765 | 0.3870968 | 0.6451613 | 0.4302326 | 0.7674419 | 0.0930233 | 0.5800433 |
| PLS | 0.7195122 | 0.8235294 | 0.5483871 | 0.6976597 | 0.5813953 | 0.2325581 | 0.9302326 | 0.6776636 |
| xgbTree | 0.7926829 | 0.9215686 | 0.5806452 | 0.6938646 | 0.3953488 | 0.6511628 | 0.1395349 | 0.5987020 |

```
write.csv(all.acc, file = "plasma_clus3_met_acc.csv")
```
