# Supplementary material for: Cheminformatics approach to exploring and modeling trait-associated metabolite profiles
Source: J Cheminform. 2019 Jun 24;11:43. doi: 10.1186/s13321-019-0366-3 (PMC6591908; doi:10.1186/s13321-019-0366-3)
Supplement: Supplementary file 2 — Additional file 2. The scripts and additional data necessary to recreate our analyses. [file 13321_2019_366_MOESM2_ESM.zip › metabochem-master/analyses/metab_classifier_serum.html]

Metabolomics ML results


# Metabolomics ML results

#### *Jeremy Ash*

#### *October 13, 2018*

## 0.1 All metabolites

```
load("ML_data.RDATA")
```

### 0.1.1 Lasso

```
all.acc <- matrix(nrow = 6, ncol = 8)
colnames(all.acc) <- c(rep("LOOCV", 4), rep("External", 4))
colnames(all.acc) <- paste(colnames(all.acc), rep(c("ACC", "SEN", "SPE", "AUC"), 2))
rownames(all.acc) <- c("Lasso", "Logit Boost", "SVM", "RF", "PLS", "xgbTree")

health_df_serum_full <- health_df_serum_full[, -c(2, 3)]
health_df_serum_full.test <- health_df_serum_full.test[, -c(2, 3)]

all.acc <- FitLasso(health_df_serum_full, health_df_serum_full.test, all.acc)
```

```
## Warning: Option grouped=FALSE enforced in cv.glmnet, since < 3 observations
## per fold
```

```
all.acc
```

```
##             LOOCV ACC LOOCV SEN LOOCV SPE LOOCV AUC External ACC
## Lasso          0.8125 0.7419355 0.8571429 0.8867676    0.6162791
## Logit Boost        NA        NA        NA        NA           NA
## SVM                NA        NA        NA        NA           NA
## RF                 NA        NA        NA        NA           NA
## PLS                NA        NA        NA        NA           NA
## xgbTree            NA        NA        NA        NA           NA
##             External SEN External SPE External AUC
## Lasso          0.9767442     0.255814    0.8020552
## Logit Boost           NA           NA           NA
## SVM                   NA           NA           NA
## RF                    NA           NA           NA
## PLS                   NA           NA           NA
## xgbTree               NA           NA           NA
```

### 0.1.2 Other ML

```
data <- health_df_serum_full
data$Health_State <- as.factor(ifelse(data$Health_State == 0, "neg", "pos"))

data.test <- health_df_serum_full.test
data.test$Health_State <- as.factor(ifelse(data.test$Health_State == 0, "neg", "pos"))
```

```
all.acc <- FitMlmodels(data, data.test, all.acc)
```

```
all.acc %>%  kable() %>%  kable_styling()
```

|  | LOOCV ACC | LOOCV SEN | LOOCV SPE | LOOCV AUC | External ACC | External SEN | External SPE | External AUC |
| --- | --- | --- | --- | --- | --- | --- | --- | --- |
| Lasso | 0.8125 | 0.7419355 | 0.8571429 | 0.8867676 | 0.6162791 | 0.9767442 | 0.2558140 | 0.8020552 |
| Logit Boost | 0.7000 | 0.8163265 | 0.5161290 | 0.6566820 | 0.6162791 | 0.7906977 | 0.4418605 | 0.7579773 |
| SVM | 0.8125 | 0.9183673 | 0.6451613 | 0.7952600 | 0.5000000 | 0.9302326 | 0.0697674 | 0.7176852 |
| RF | 0.6625 | 1.0000000 | 0.1290323 | 0.6379197 | 0.5116279 | 0.9767442 | 0.0465116 | 0.8345051 |
| PLS | 0.7875 | 0.8979592 | 0.6129032 | 0.7564187 | 0.6860465 | 0.5813953 | 0.7906977 | 0.7322877 |
| xgbTree | 0.7375 | 0.8775510 | 0.5161290 | 0.6998025 | 0.6162791 | 0.8372093 | 0.3953488 | 0.7836668 |

```
write.csv(all.acc, file = "serum_all_met_acc.csv")
```

## 0.2 Significant metabolites

### 0.2.1 Lasso

```
all.acc <- matrix(nrow = 6, ncol = 8)
colnames(all.acc) <- c(rep("LOOCV", 4), rep("External", 4))
colnames(all.acc) <- paste(colnames(all.acc), rep(c("ACC", "SEN", "SPE", "AUC"), 2))
rownames(all.acc) <- c("Lasso", "Logit Boost", "SVM", "RF", "PLS", "xgbTree")

health_df_serum_full <- health_df_serum_full[, c(1, serum.met.idx - 3)]
health_df_serum_full.test <- health_df_serum_full.test[, c(1, serum.met.idx - 3)]

all.acc <- FitLasso(health_df_serum_full, health_df_serum_full.test, all.acc)
```

```
## Warning: Option grouped=FALSE enforced in cv.glmnet, since < 3 observations
## per fold
```

### 0.2.2 Other ML

```
data <- health_df_serum_full
data$Health_State <- as.factor(ifelse(data$Health_State == 0, "neg", "pos"))

data.test <- health_df_serum_full.test
data.test$Health_State <- as.factor(ifelse(data.test$Health_State == 0, "neg", "pos"))
```

```
all.acc <- FitMlmodels(data, data.test, all.acc)
```

```
## note: only 4 unique complexity parameters in default grid. Truncating the grid to 4 .
```

```
all.acc %>%  kable() %>%  kable_styling()
```

|  | LOOCV ACC | LOOCV SEN | LOOCV SPE | LOOCV AUC | External ACC | External SEN | External SPE | External AUC |
| --- | --- | --- | --- | --- | --- | --- | --- | --- |
| Lasso | 0.7250 | 0.5483871 | 0.8367347 | 0.8044766 | 0.6627907 | 1.0000000 | 0.3255814 | 0.8626284 |
| Logit Boost | 0.8000 | 0.8571429 | 0.7096774 | 0.8311389 | 0.6395349 | 0.3255814 | 0.9534884 | 0.8015143 |
| SVM | 0.8125 | 0.8775510 | 0.7096774 | 0.8538512 | 0.6976744 | 0.3953488 | 1.0000000 | 0.8647918 |
| RF | 0.7625 | 0.8979592 | 0.5483871 | 0.8179724 | 0.7558140 | 0.5581395 | 0.9534884 | 0.8618172 |
| PLS | 0.7875 | 0.9387755 | 0.5483871 | 0.8400263 | 0.6627907 | 0.3720930 | 0.9534884 | 0.8464035 |
| xgbTree | 0.8375 | 0.8367347 | 0.8387097 | 0.8123766 | 0.5930233 | 0.1860465 | 1.0000000 | 0.8066522 |

```
write.csv(all.acc, file = "serum_sig_met_acc.csv")
```

## 0.3 Cluster metabolites

### 0.3.1 Lasso

```
all.acc <- matrix(nrow = 6, ncol = 8)
colnames(all.acc) <- c(rep("LOOCV", 4), rep("External", 4))
colnames(all.acc) <- paste(colnames(all.acc), rep(c("ACC", "SEN", "SPE", "AUC"), 2))
rownames(all.acc) <- c("Lasso", "Logit Boost", "SVM", "RF", "PLS", "xgbTree")

health_df_serum_full <- health_df_serum_full[, c(1, 2, 3, 4)]
health_df_serum_full.test <- health_df_serum_full.test[, c(1, 2, 3, 4)]

all.acc <- FitLasso(health_df_serum_full, health_df_serum_full.test, all.acc)
```

```
## Warning: Option grouped=FALSE enforced in cv.glmnet, since < 3 observations
## per fold
```

### 0.3.2 Other ML

```
data <- health_df_serum_full
data$Health_State <- as.factor(ifelse(data$Health_State == 0, "neg", "pos"))

data.test <- health_df_serum_full.test
data.test$Health_State <- as.factor(ifelse(data.test$Health_State == 0, "neg", "pos"))
```

```
all.acc <- FitMlmodels(data, data.test, all.acc)
```

```
## note: only 2 unique complexity parameters in default grid. Truncating the grid to 2 .
```

```
all.acc %>%  kable() %>%  kable_styling()
```

|  | LOOCV ACC | LOOCV SEN | LOOCV SPE | LOOCV AUC | External ACC | External SEN | External SPE | External AUC |
| --- | --- | --- | --- | --- | --- | --- | --- | --- |
| Lasso | 0.6250 | 0.4516129 | 0.7346939 | 0.6813693 | 0.8372093 | 0.9767442 | 0.6976744 | 0.8507301 |
| Logit Boost | 0.7000 | 0.9795918 | 0.2580645 | 0.6201448 | 0.6511628 | 0.8139535 | 0.4883721 | 0.7539210 |
| SVM | 0.7625 | 0.7755102 | 0.7419355 | 0.7511521 | 0.8488372 | 0.7209302 | 0.9767442 | 0.8555976 |
| RF | 0.6125 | 1.0000000 | 0.0000000 | 0.5941409 | 0.5000000 | 1.0000000 | 0.0000000 | 0.7658194 |
| PLS | 0.7000 | 0.6734694 | 0.7419355 | 0.7149440 | 0.8255814 | 0.6744186 | 0.9767442 | 0.8615468 |
| xgbTree | 0.7250 | 0.8367347 | 0.5483871 | 0.7294273 | 0.6279070 | 0.5581395 | 0.6976744 | 0.7133586 |

```
write.csv(all.acc, file = "serum_cluster_acc.csv")
```
