# Supplementary material for: Cheminformatics approach to exploring and modeling trait-associated metabolite profiles
Source: J Cheminform. 2019 Jun 24;11:43. doi: 10.1186/s13321-019-0366-3 (PMC6591908; doi:10.1186/s13321-019-0366-3)
Supplement: Supplementary file 2 — Additional file 2. The scripts and additional data necessary to recreate our analyses. [file 13321_2019_366_MOESM2_ESM.zip › metabochem-master/analyses/test.html]

Untitled


# Untitled

#### *Jeremy Ash*

#### *4/23/2019*

# Cheminformatics Based Approach to Exploring and Modeling Trait-Associated Metabolite Profiles

**Jeremy R. Ash, Melaine A. Kuenemann, Daniel Rotroff, Alison Motsinger-Reif, and Denis Fourches**

To run any of the r scripts provided, open the metabochem.Rproj file in Rstudio. Run the scripts within the project, so that they can find the relevant paths on your machine.

## Files

- The analyses folder contains three rmarkdown files and one r script which performs the majority of the analyses reported in the paper
  - *differential\_analyses.rmd*: performs the
- The outputs folder contains data for each participant and a summary data sheeet (bageldonutdata.txt)
- The participant list is in bageldonut\_participants.xls (password protected)
