# Supplementary figures and images for: Cheminformatics approach to exploring and modeling trait-associated metabolite profiles
Source: J Cheminform. 2019 Jun 24;11:43. doi: 10.1186/s13321-019-0366-3 (PMC6591908; doi:10.1186/s13321-019-0366-3)

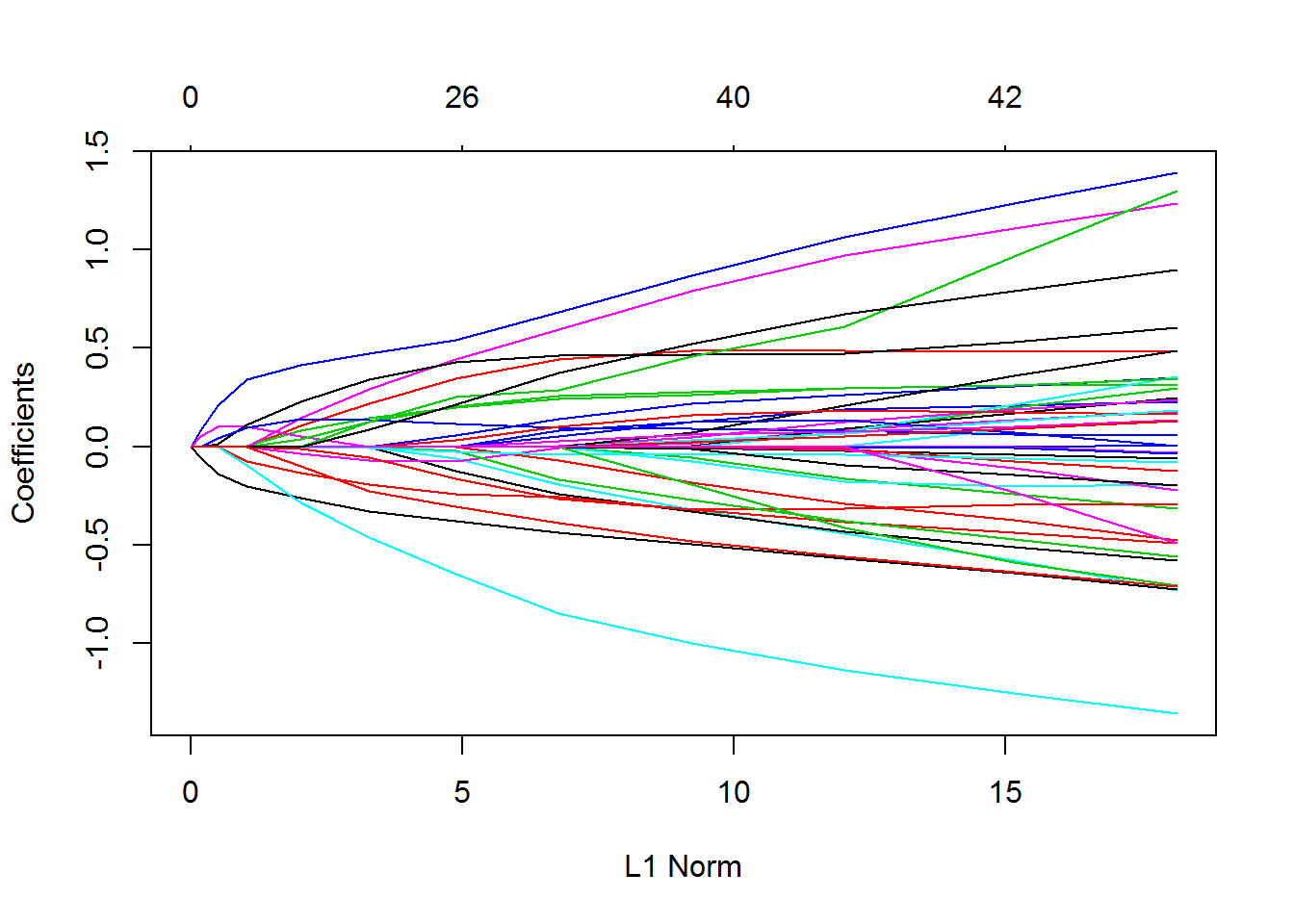

Supplement: Supplementary file 2 — Additional file 2. The scripts and additional data necessary to recreate our analyses. [file 13321_2019_366_MOESM2_ESM.zip › metabochem-master/analyses/metab_classifier_plasma_files/figure-html/unnamed-chunk-1-1.png]

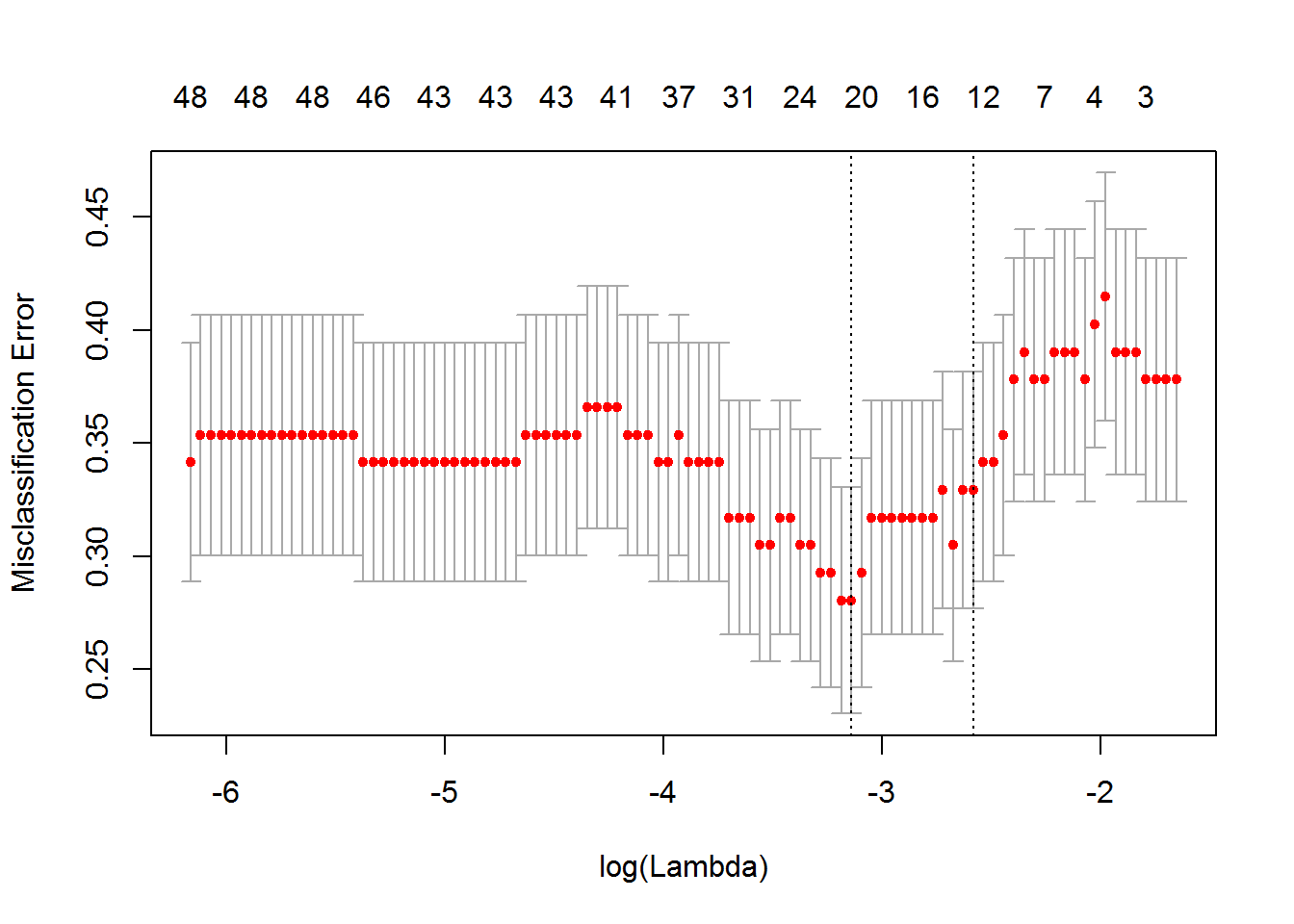

Supplement: Supplementary file 2 — Additional file 2. The scripts and additional data necessary to recreate our analyses. [file 13321_2019_366_MOESM2_ESM.zip › metabochem-master/analyses/metab_classifier_plasma_files/figure-html/unnamed-chunk-1-2.png]

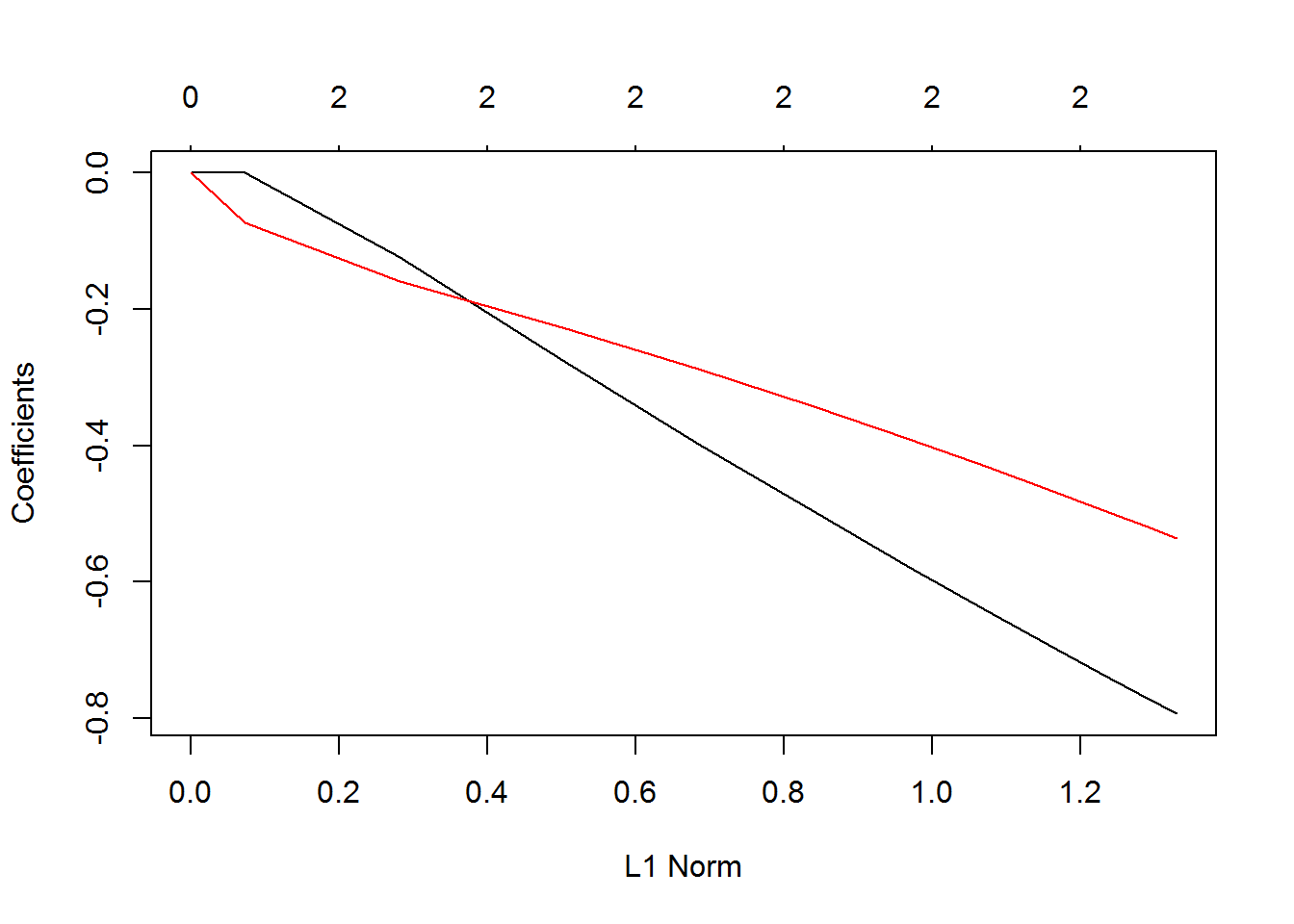

Supplement: Supplementary file 2 — Additional file 2. The scripts and additional data necessary to recreate our analyses. [file 13321_2019_366_MOESM2_ESM.zip › metabochem-master/analyses/metab_classifier_plasma_files/figure-html/unnamed-chunk-13-1.png]

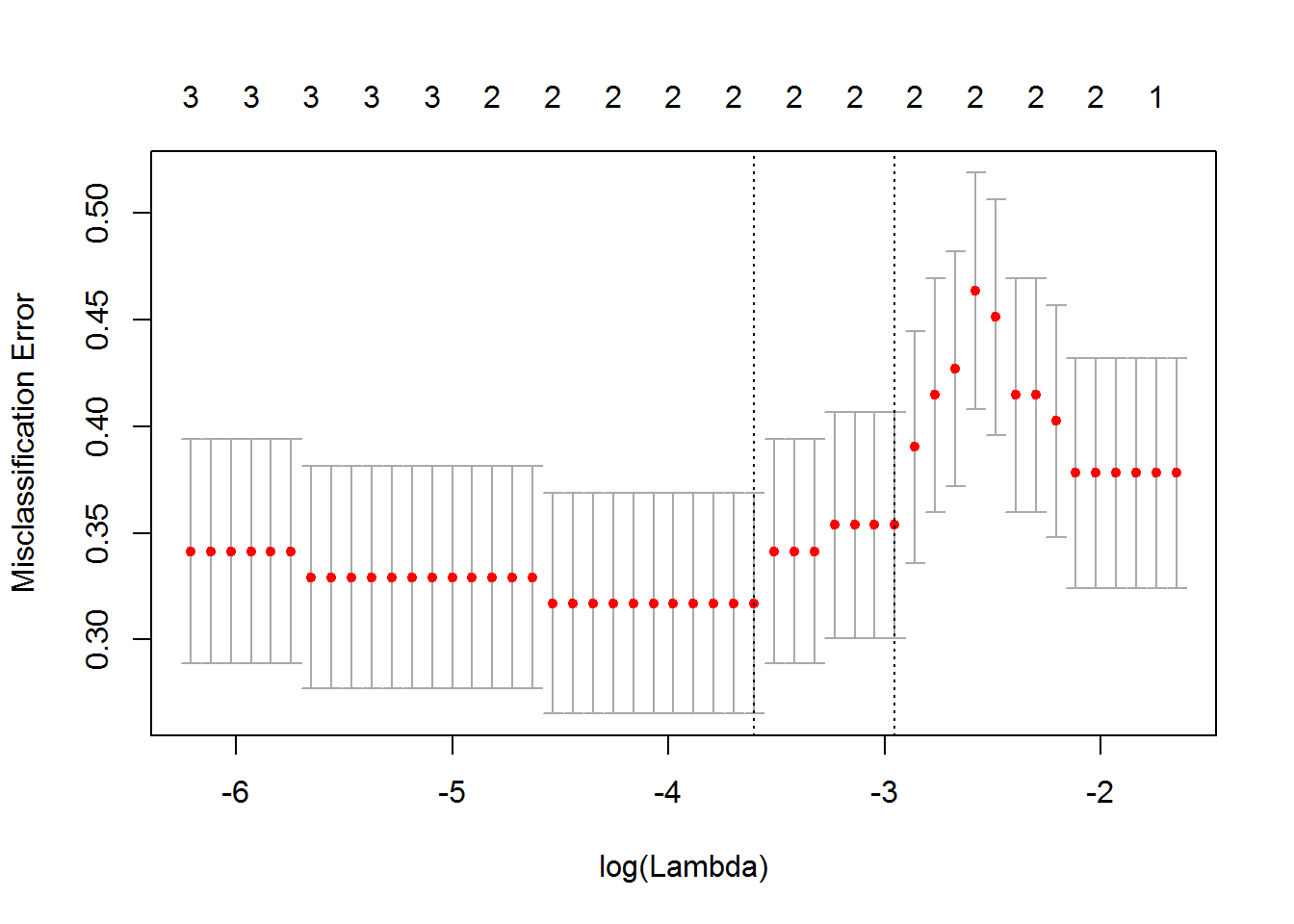

Supplement: Supplementary file 2 — Additional file 2. The scripts and additional data necessary to recreate our analyses. [file 13321_2019_366_MOESM2_ESM.zip › metabochem-master/analyses/metab_classifier_plasma_files/figure-html/unnamed-chunk-13-2.png]

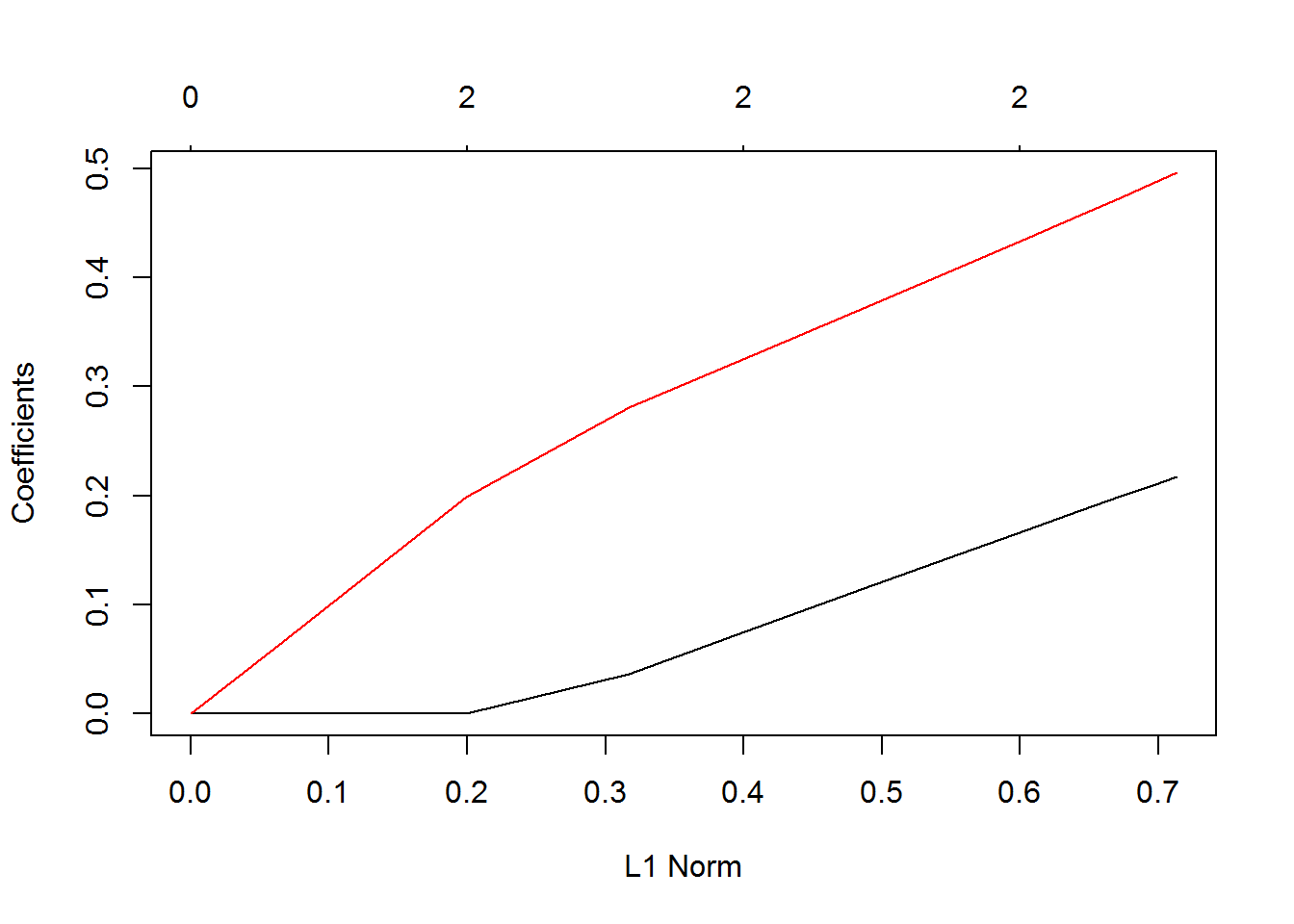

Supplement: Supplementary file 2 — Additional file 2. The scripts and additional data necessary to recreate our analyses. [file 13321_2019_366_MOESM2_ESM.zip › metabochem-master/analyses/metab_classifier_plasma_files/figure-html/unnamed-chunk-17-1.png]

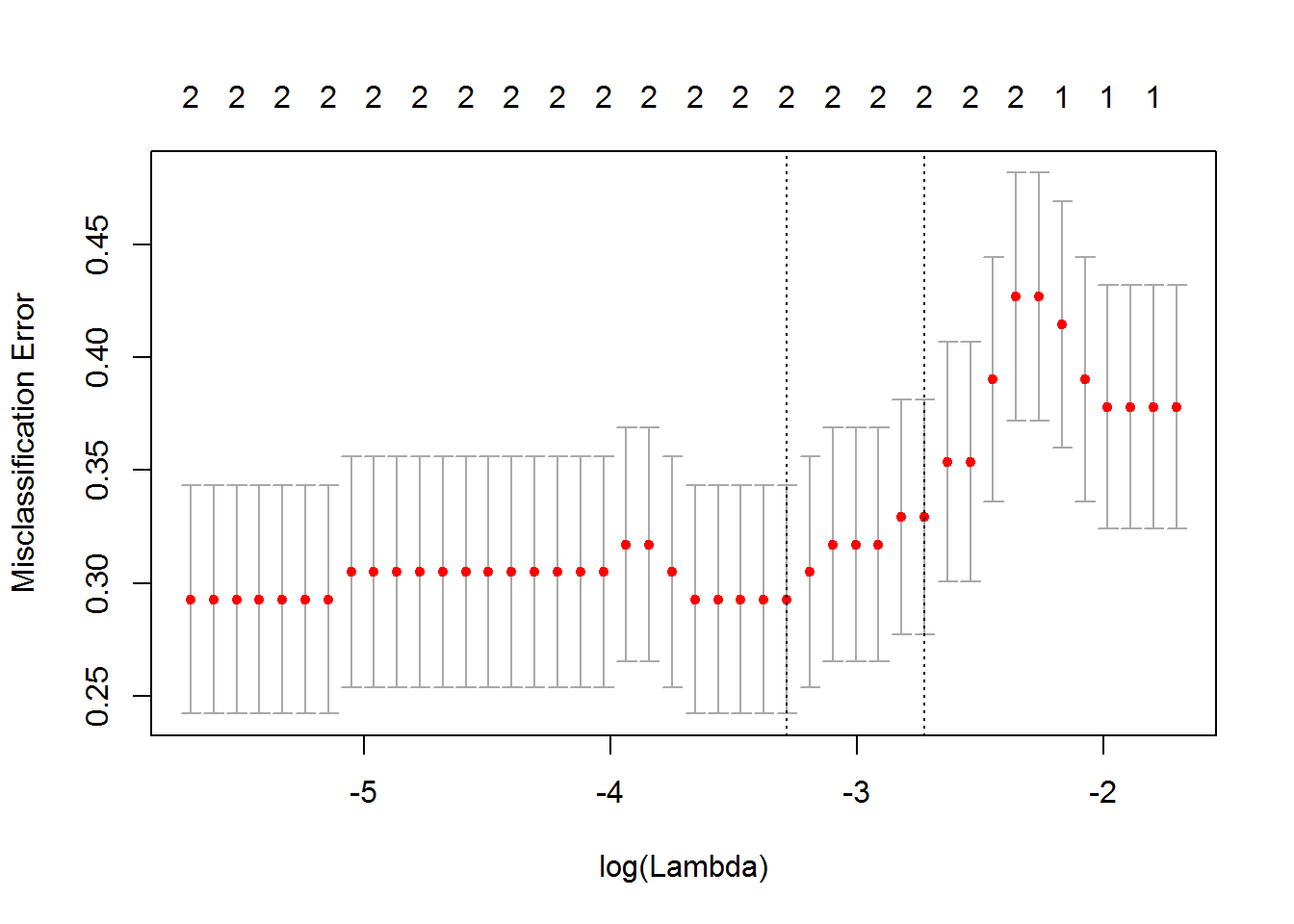

Supplement: Supplementary file 2 — Additional file 2. The scripts and additional data necessary to recreate our analyses. [file 13321_2019_366_MOESM2_ESM.zip › metabochem-master/analyses/metab_classifier_plasma_files/figure-html/unnamed-chunk-17-2.png]

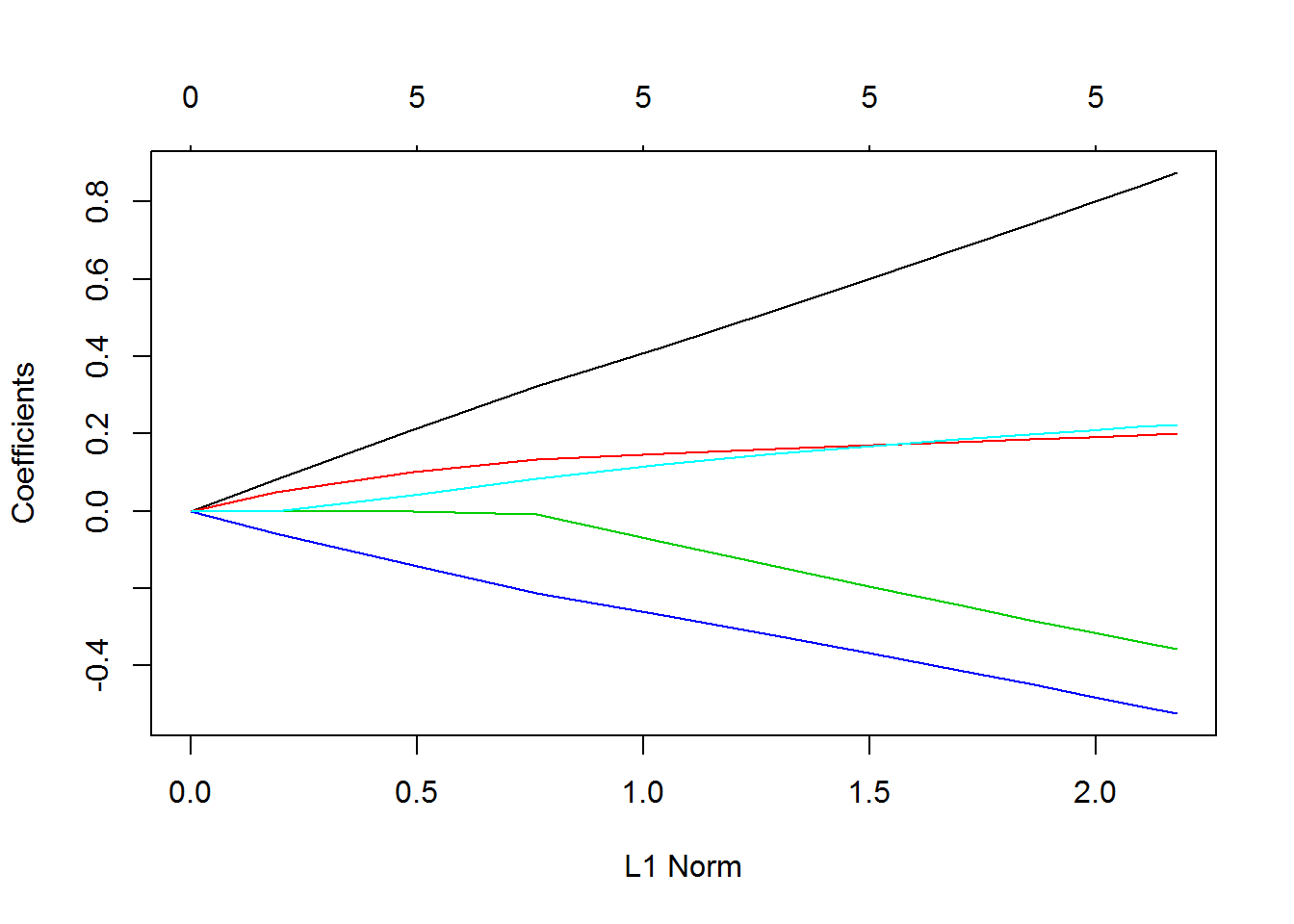

Supplement: Supplementary file 2 — Additional file 2. The scripts and additional data necessary to recreate our analyses. [file 13321_2019_366_MOESM2_ESM.zip › metabochem-master/analyses/metab_classifier_plasma_files/figure-html/unnamed-chunk-5-1.png]

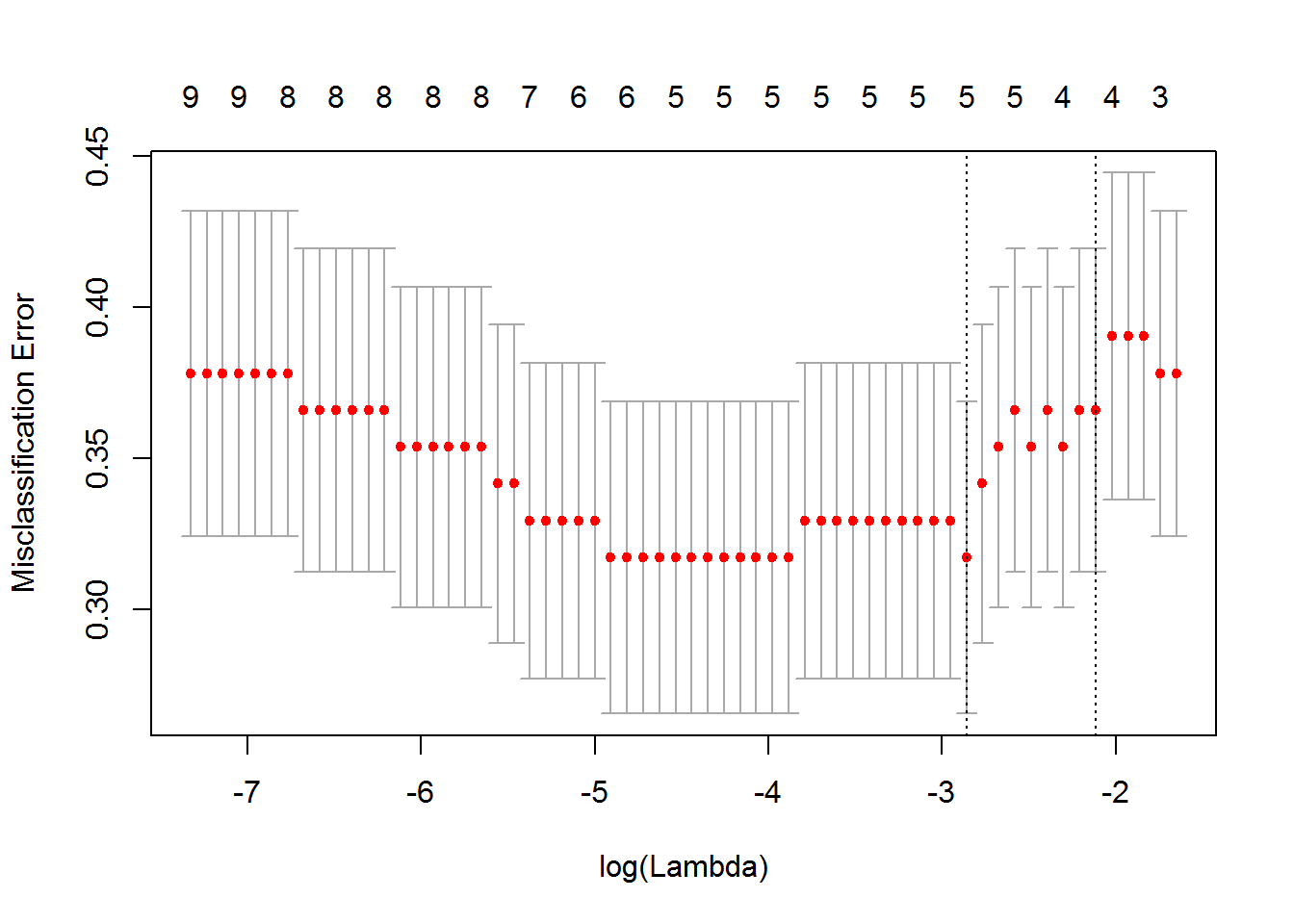

Supplement: Supplementary file 2 — Additional file 2. The scripts and additional data necessary to recreate our analyses. [file 13321_2019_366_MOESM2_ESM.zip › metabochem-master/analyses/metab_classifier_plasma_files/figure-html/unnamed-chunk-5-2.png]

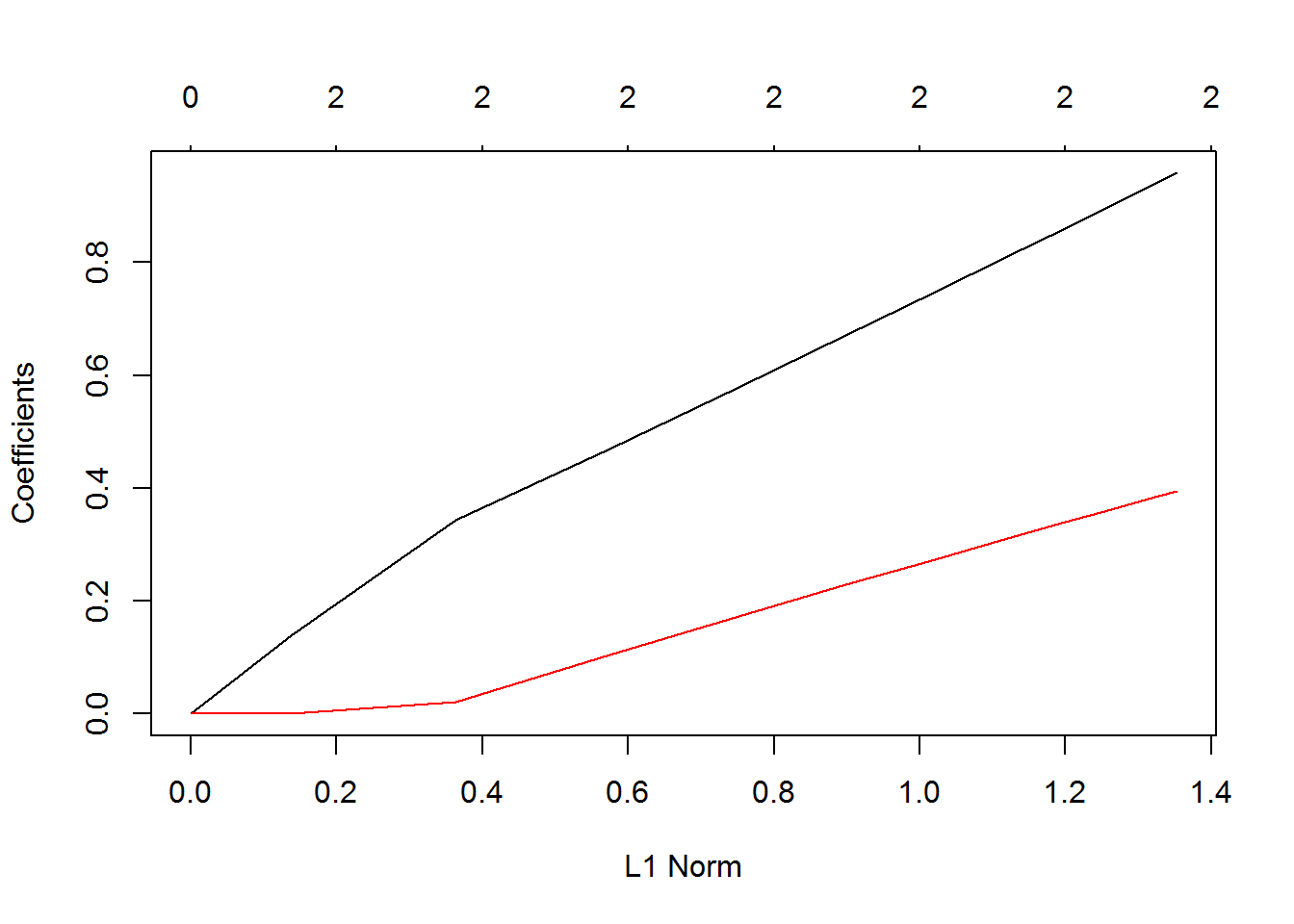

Supplement: Supplementary file 2 — Additional file 2. The scripts and additional data necessary to recreate our analyses. [file 13321_2019_366_MOESM2_ESM.zip › metabochem-master/analyses/metab_classifier_plasma_files/figure-html/unnamed-chunk-9-1.png]

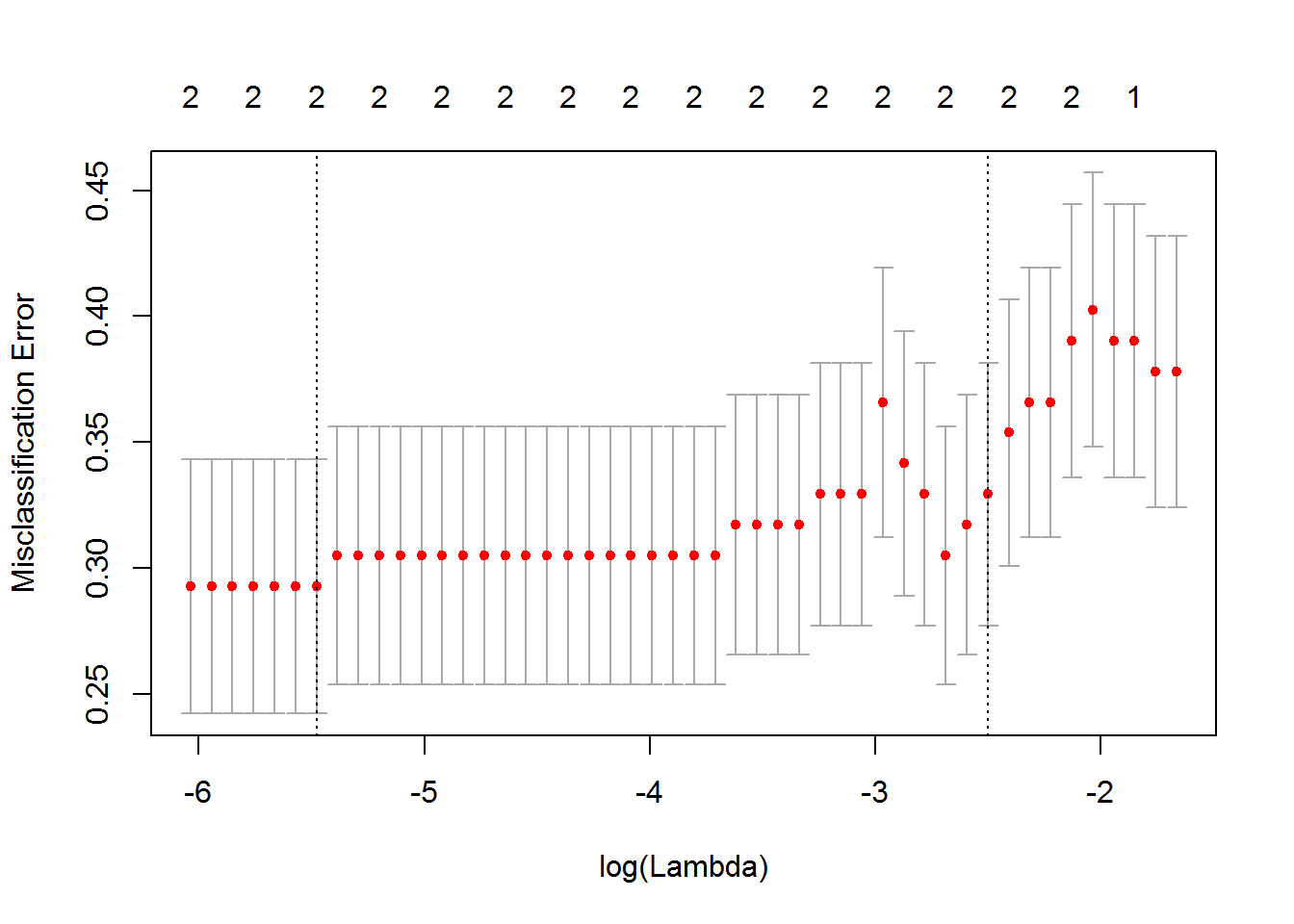

Supplement: Supplementary file 2 — Additional file 2. The scripts and additional data necessary to recreate our analyses. [file 13321_2019_366_MOESM2_ESM.zip › metabochem-master/analyses/metab_classifier_plasma_files/figure-html/unnamed-chunk-9-2.png]

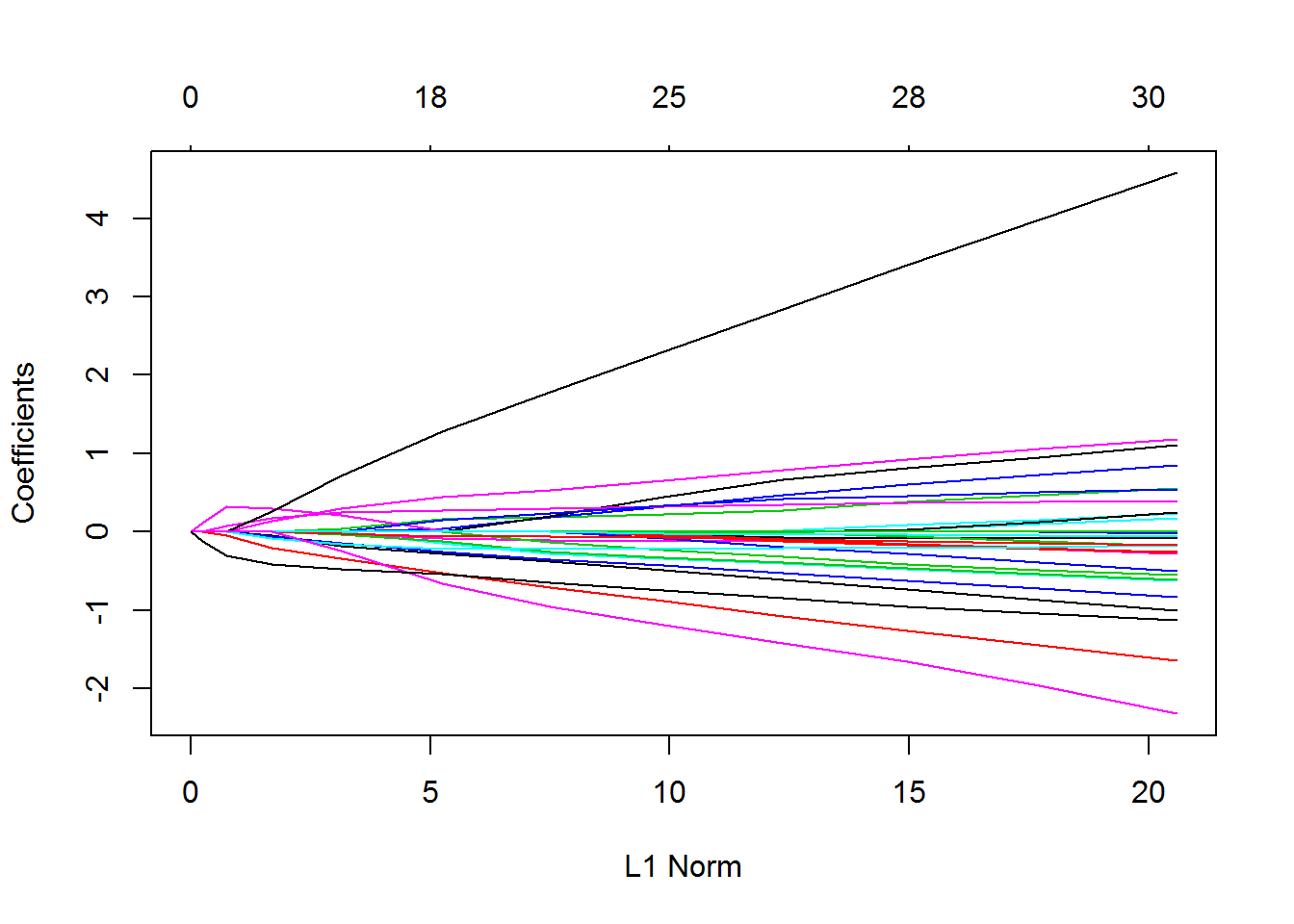

Supplement: Supplementary file 2 — Additional file 2. The scripts and additional data necessary to recreate our analyses. [file 13321_2019_366_MOESM2_ESM.zip › metabochem-master/analyses/metab_classifier_serum_files/figure-html/unnamed-chunk-1-1.png]

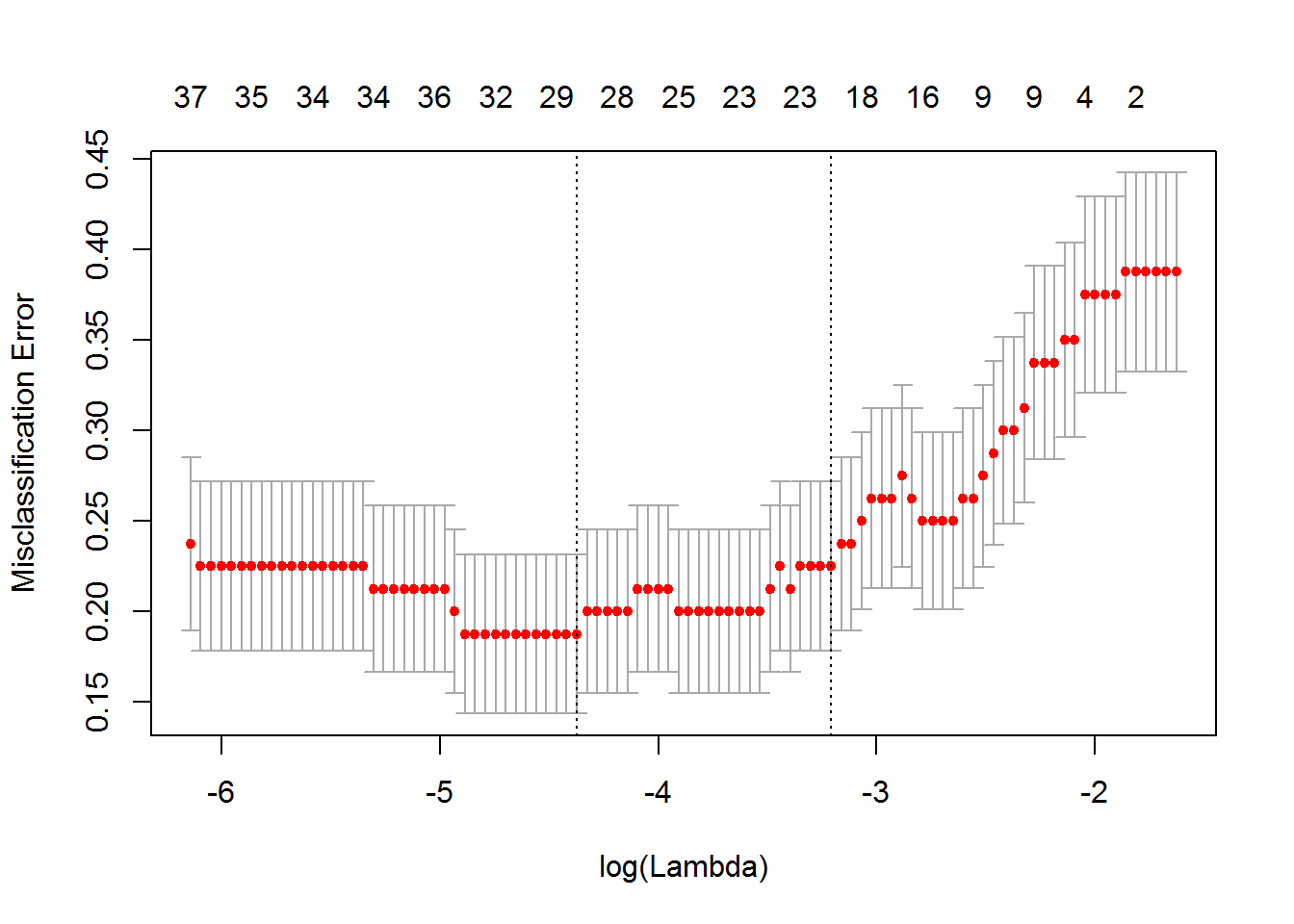

Supplement: Supplementary file 2 — Additional file 2. The scripts and additional data necessary to recreate our analyses. [file 13321_2019_366_MOESM2_ESM.zip › metabochem-master/analyses/metab_classifier_serum_files/figure-html/unnamed-chunk-1-2.png]

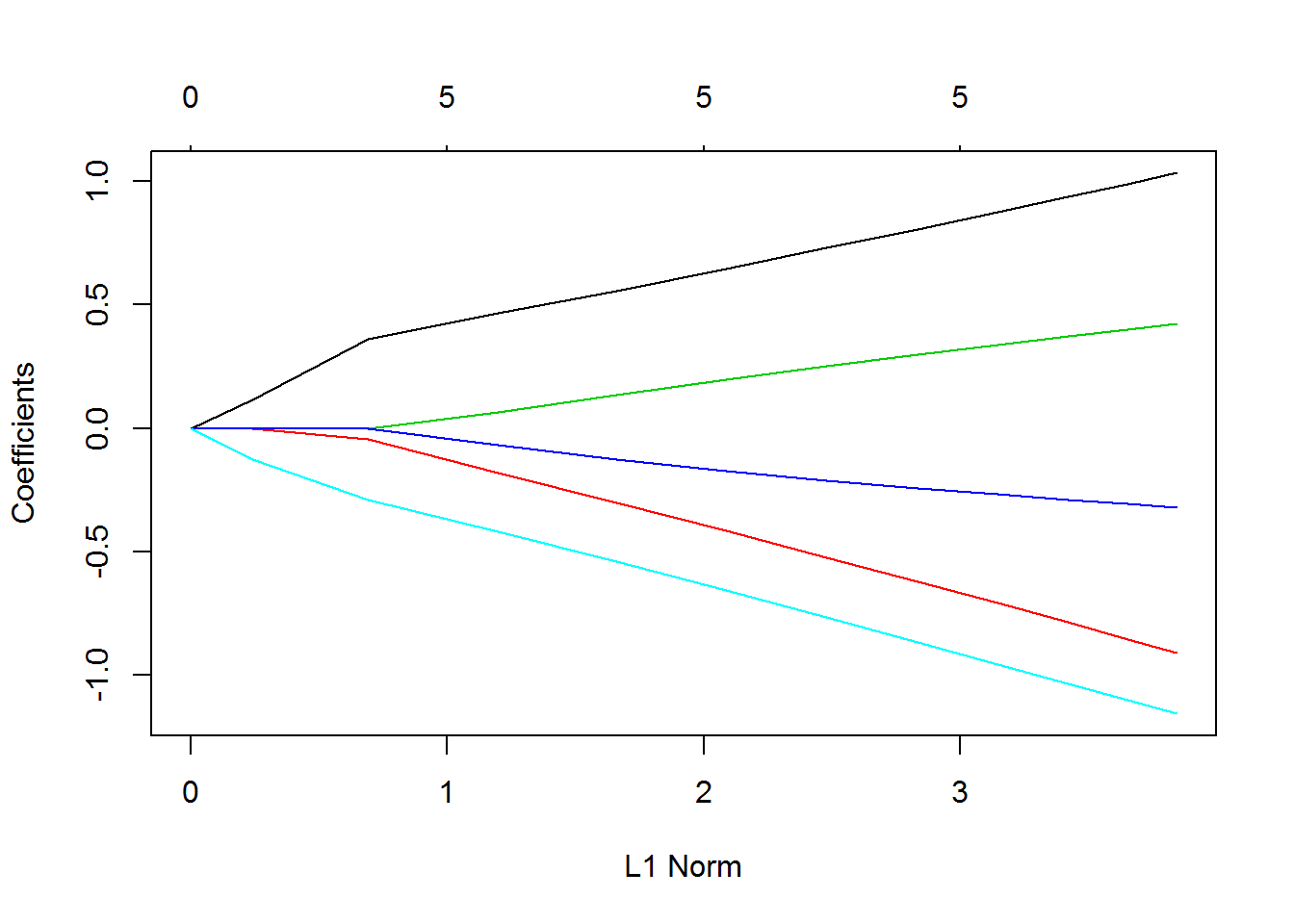

Supplement: Supplementary file 2 — Additional file 2. The scripts and additional data necessary to recreate our analyses. [file 13321_2019_366_MOESM2_ESM.zip › metabochem-master/analyses/metab_classifier_serum_files/figure-html/unnamed-chunk-5-1.png]

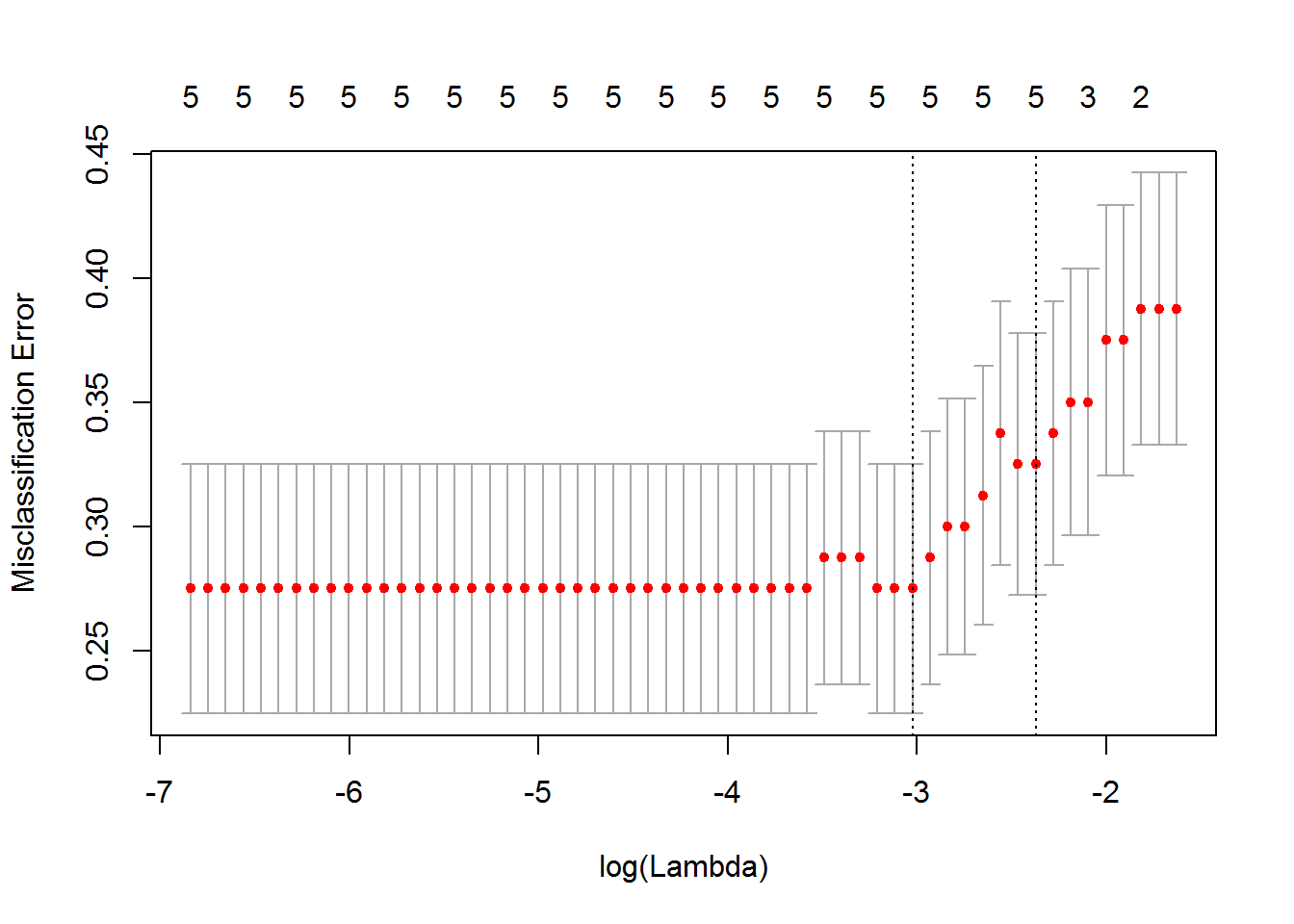

Supplement: Supplementary file 2 — Additional file 2. The scripts and additional data necessary to recreate our analyses. [file 13321_2019_366_MOESM2_ESM.zip › metabochem-master/analyses/metab_classifier_serum_files/figure-html/unnamed-chunk-5-2.png]

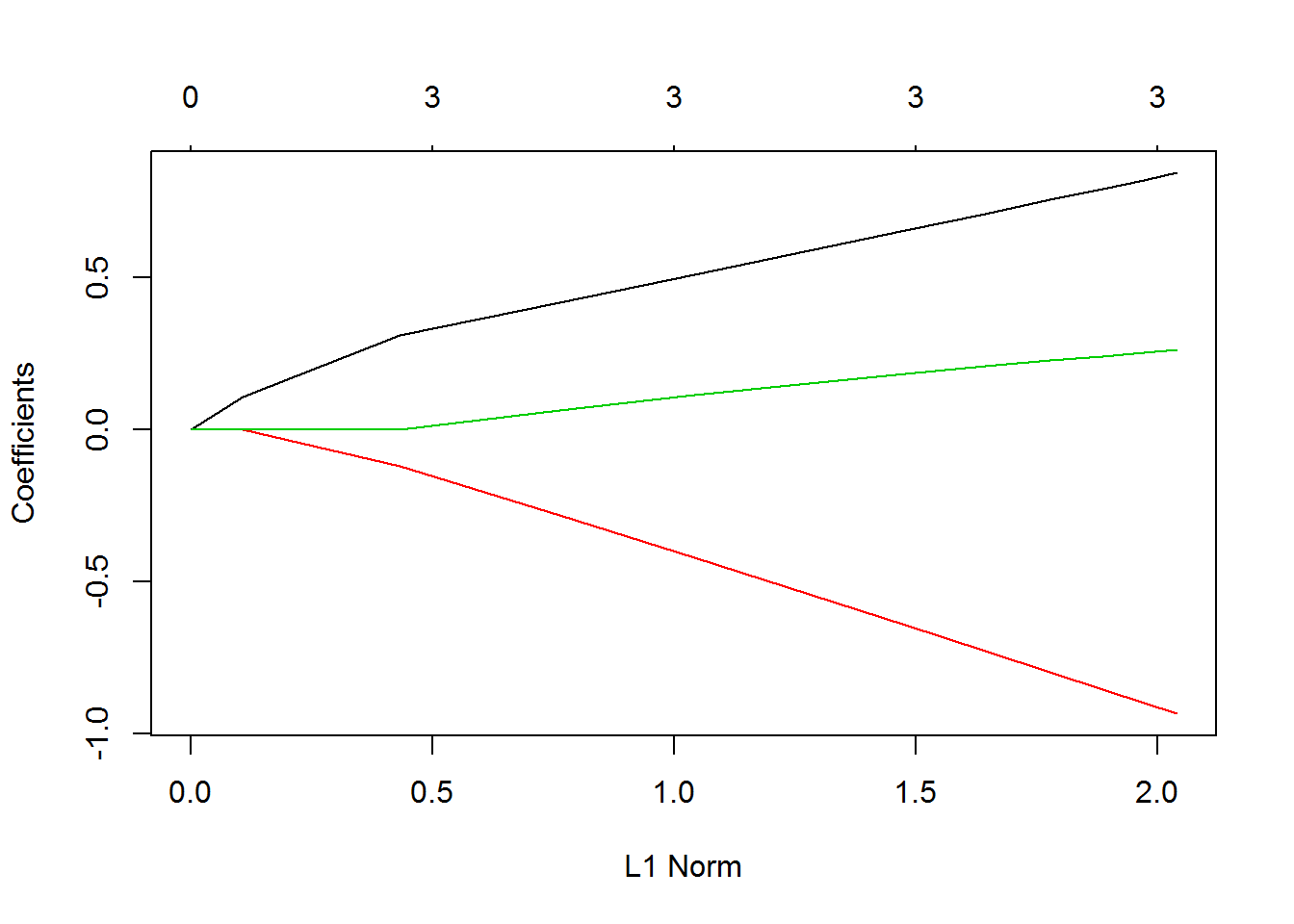

Supplement: Supplementary file 2 — Additional file 2. The scripts and additional data necessary to recreate our analyses. [file 13321_2019_366_MOESM2_ESM.zip › metabochem-master/analyses/metab_classifier_serum_files/figure-html/unnamed-chunk-9-1.png]

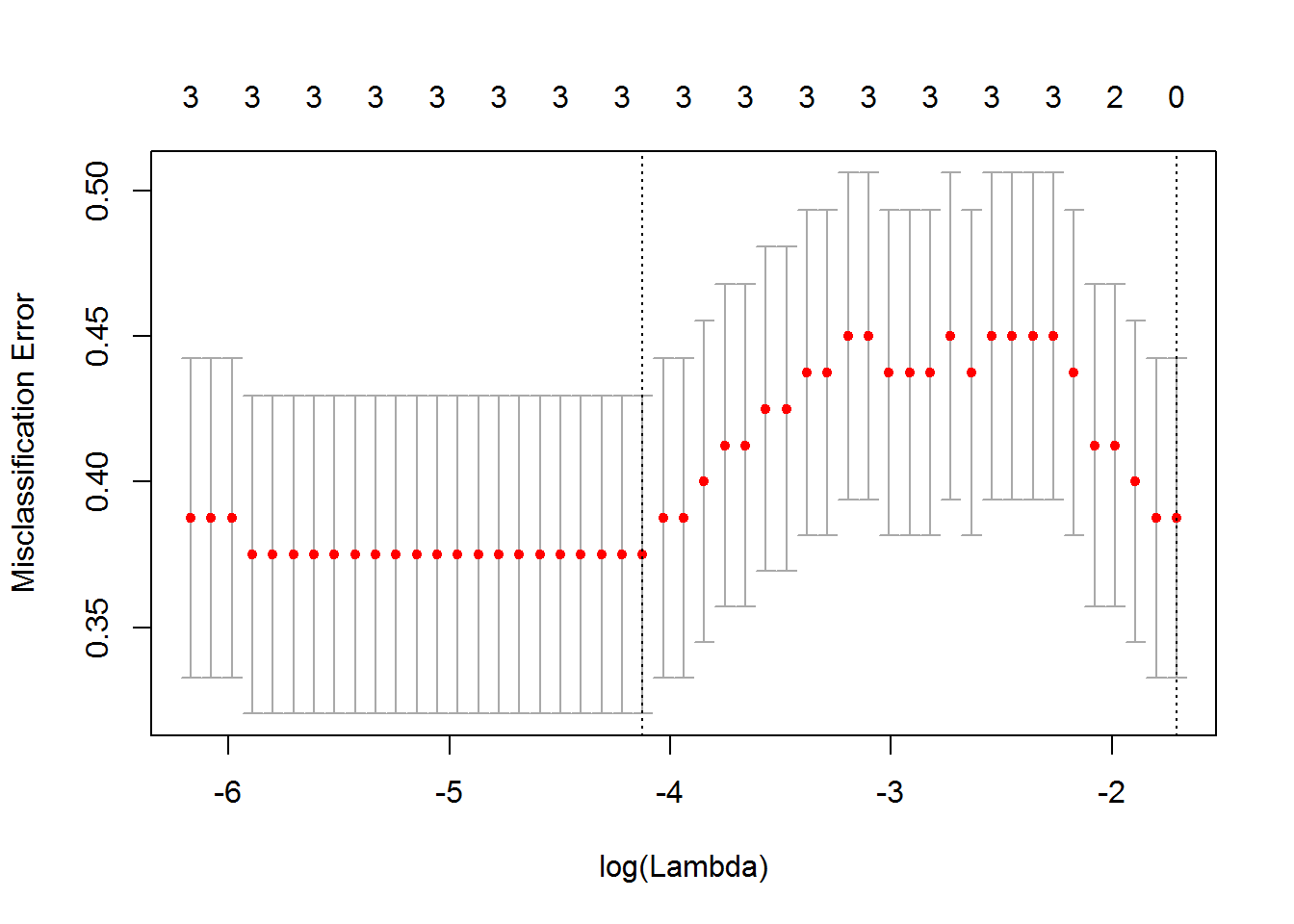

Supplement: Supplementary file 2 — Additional file 2. The scripts and additional data necessary to recreate our analyses. [file 13321_2019_366_MOESM2_ESM.zip › metabochem-master/analyses/metab_classifier_serum_files/figure-html/unnamed-chunk-9-2.png]
